# In the absence of mitochondrial fusion unequal segregation of mitochondria drives mtDNA loss

**DOI:** 10.1038/s44319-026-00794-5

**Published:** 2026-05-14

**Authors:** Lisa Dengler, Francesco Padovani, Bianca Lemke, Rebecca Brugger, Alissa Benedikt, Benedikt Westermann, Boris Maček, Kurt M Schmoller, Jennifer C Ewald

**Affiliations:** 1 Molecular Cell Biology, Institute of Cell Biology, University of Tübingen, Tübingen, Germany; 2 Institute of Functional Epigenetics, Molecular Targets and Therapeutics Center, Helmholtz Zentrum München, Munich, Germany; 3 Quantitative Proteomics, Institute of Cell Biology, University of Tübingen, Tübingen, Germany; 4 Cell Biology, University of Bayreuth, Bayreuth, Germany

**Keywords:** Metabolism, Organelles

## Abstract

Mitochondrial biogenesis and inheritance must be tightly coordinated with cell division to maintain mitochondrial function and cell survival. The dynamics of the mitochondrial network, including fusion and fission, are essential for mitochondrial inheritance and quality control. In budding yeast, simultaneous inhibition of both processes compromises mitochondrial DNA (mtDNA) integrity, increasing the frequency of petite cells. Loss of fusion alone completely eliminates mtDNA. Although this has been known for decades, why mtDNA is lost remained unclear. Here, we examine the effects of impaired mitochondrial fusion by depleting the mitofusin Fzo1. By analyzing over thirty thousand single cells across their cell cycles, we show that Fzo1-depletion induces rapid mitochondrial fragmentation and loss of membrane potential, followed by progressive declines in mtDNA content and growth rate. During division, Fzo1-depleted daughters inherit disproportionately large mitochondrial amounts, leaving mothers with too little. This imbalance, combined with an inability to upregulate compensatory mtDNA synthesis, drives rapid mtDNA loss. Our results reveal how fusion defects cause mtDNA loss and mitochondrial dysfunction, which might have implications for diseases linked to impaired fusion.

## Synopsis

Loss of yeast mitofusin Fzo1 alters mitochondrial morphology, causing unequal mitochondrial distribution between mother and daughter cells and impaired mtDNA synthesis. These combined defects cause a rapid, progressive depletion of mtDNA across the population, leading to the emergence of petite cells.

- Depletion of the mitofusin Fzo1 induces a rapid, but reversible change in mitochondrial morphology.
- Altered morphology leads to unequal segregation of mitochondria and mitochondrial DNA during division and failure to upregulate compensatory mtDNA synthesis.
- Unequal inheritance combined with impaired synthesis drives progressive mtDNA loss and leads to the petite phenotype.
- Increased cell-to-cell heterogeneity can lead to a decline in cell function on the population level.

## Abstract (web summary)

Loss of yeast mitofusin Fzo1 alters mitochondrial morphology, causing unequal mitochondrial distribution between mother and daughter cells and impaired mtDNA synthesis. These combined defects cause a rapid, progressive depletion of mtDNA across the population, leading to the emergence of petite cells.

## Graphical abstract

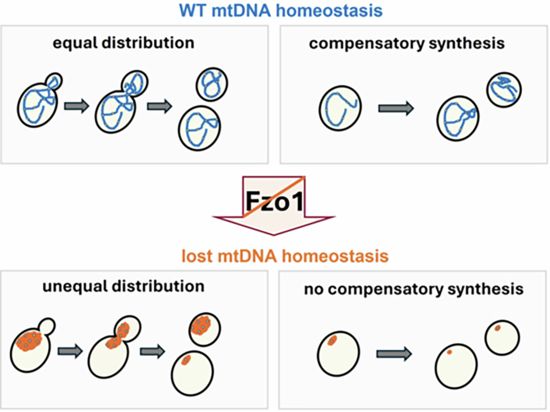

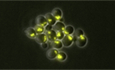

## Introduction

Mitochondrial biogenesis needs to be tightly coordinated with cell proliferation. Since mitochondria cannot be formed de novo, their proper inheritance during the cell division cycle is essential for cell survival. The accurate distribution and quality control during cell division are enabled by the dynamics of the mitochondrial network, including fusion and fission. Mitochondrial fusion was suggested to increase content mixing within the mitochondrial network, while fission helps to remove non-functional mitochondria through mitophagy (Abeliovich, 2023; Friedman and Nunnari, 2014; Khan et al, 2024; Quintana-Cabrera and Scorrano, 2023; Westermann, 2010). In humans, impaired mitochondrial fusion leads to a strong reduction of mitochondrial DNA (mtDNA) (Silva Ramos et al, 2019). Accordingly, fusion defects are associated with numerous diseases, such as peripheral neuropathy and optic atrophy, which are frequently connected to the depletion of mtDNA (Alexander et al, 2000; Delettre et al, 2000; Züchner et al, 2004). However, how and why the loss of fusion leads to a depletion of mtDNA is still not understood.

Unlike human cells, the budding yeast *Saccharomyces cerevisiae* can tolerate the complete loss of mtDNA, making it an ideal system to unravel the connection between fusion, fission, and mtDNA maintenance. The budding yeast mtDNA encodes four subunits of the respiratory chain, three subunits of the ATP synthase (complex V), and one subunit of the mitochondrial ribosome. Loss of mtDNA not only leads to the loss of mtDNA encoded subunits of the respiratory chain, but also a strong reduction of the nuclear encoded respiratory chain subunits (Dagsgaard et al, 2001; Vowinckel et al, 2021). Respiration is involved in maintaining the proton gradient across the inner membrane, which is a major part of the mitochondrial membrane potential (MMP). Maintaining a high MMP is required for mitochondrial import of nuclear-encoded mitochondrial proteins, which are crucial for mitochondrial biogenesis and cellular health. Thus, while loss of mtDNA is not lethal to yeast, its loss results in the inability to respire and reduces growth on media containing limiting amounts of non-fermentable carbon sources, resulting in the so-called “petite phenotype” (Contamine and Picard, 2000; Stenger et al, 2020).

An increased frequency of petite cells is observed when fusion and fission of mitochondria are simultaneously inhibited, which likely leads to defects in mitochondrial DNA (mtDNA) integrity (Osman et al, 2015; Wisniewski et al, 2024). Loss of mitochondrial fission alone only mildly increases petite frequency, whereas the absence of fusion has severe effects: Cells lacking the mitofusin Fzo1, a GTPase required for fusion of the mitochondrial outer membrane, show complete loss of mtDNA in the entire population. Even though this effect has been known for almost 30 years (Hermann et al, 1998; Rapaport et al, 1998), it is still unclear why the fragmentation of mitochondria lacking Fzo1 causes the loss of their genome. Exploring the cause has been hampered by the many defects of the deletion mutant, including fragmented mitochondria, a reduced MMP, and slow growth. To disentangle primary causes and secondary effects, it is necessary to monitor cells immediately as the phenotype emerges.

Here, we revealed the dynamics of mtDNA loss in fusion-deficient cells by controlled depletion of Fzo1. We analyzed mitochondrial morphology, distribution, and function in tens of thousands of dividing cells using live-cell imaging. While the primary phenotype, the fragmentation of mitochondria, is established within less than an hour, the loss of mtDNA and reduction of growth are manifested over 21 h (10 generations). We found that despite an initial delay of mitochondrial transport to the bud, at the end of the cell cycle, an unusually large amount of mitochondria is transported into the bud, resulting in mother cells with low mitochondrial concentration and mtDNA content. Cells with low mitochondrial content have a reduced ability to synthesize mtDNA-encoded proteins, resulting in the rapid development of the ∆*fzo1* phenotype.

## Results

### Loss of Fzo1 leads to rapid changes in mitochondrial morphology, followed by a gradual decline in growth rate

Complex phenotypes of mutants are difficult to study if they are characterized by multiple interdependent defects, as in the ∆*fzo1* mutant. The cause-and-effect relationships between the individual defects are often not clearly understood, and therefore the trigger of the phenotype is often unclear. To unravel the primary causes of such complex phenotypes, a system is required that removes the protein of interest specifically and rapidly. Here, we use the AID (auxin-induced degron) system, which enables induced protein degradation within a short time frame without additional perturbations to the cell (Yesbolatova et al, 2020). In the presence of auxin, the TIR protein, an adapter for E3 ubiquitin ligases, induces the degradation of AID-tagged proteins.

**Figure 1:**
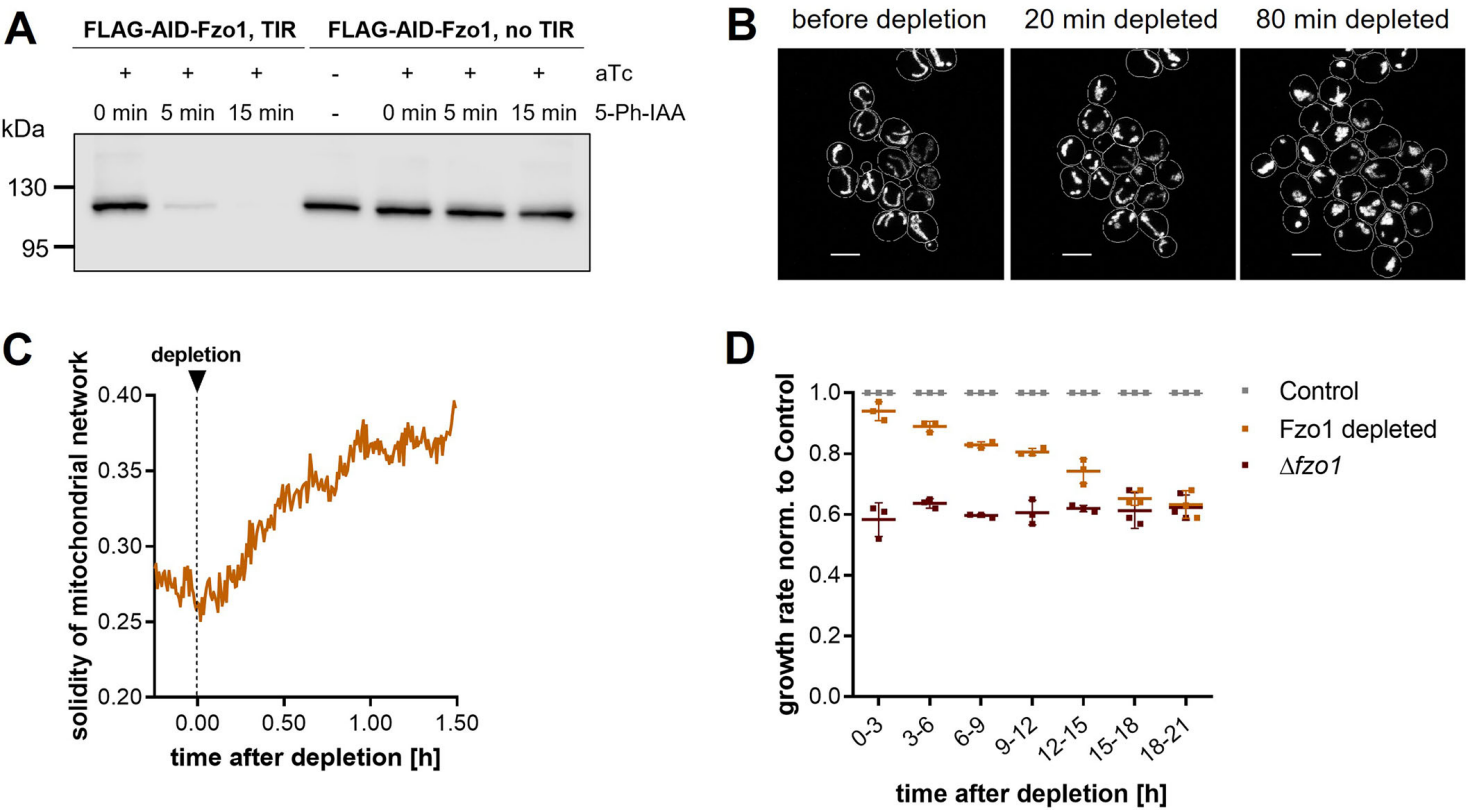
Loss of Fzo1 leads to rapid changes in mitochondrial morphology. Cells were grown in synthetic complete (SC) medium containing 1% glucose, and Fzo1 depletion was induced at the indicated times. (**A**) Western blot analysis of FLAG-AID-Fzo1. TIR expression was induced by the addition of anhydrotetracycline (aTC) 2 h before depletion. Then, Fzo1 depletion was induced by the addition of 5-Ph-IAA at *t* = 0 min. A representative blot from two biological replicates is shown. (**B**) Example pictures of confocal microscopy of mitochondria. Maximum z-projections of mitochondria visualized by preSu9-mCardinal before and after depletion of Fzo1 are shown. Scale bar = 5 µm. (**C**) Solidity of the mitochondrial network after Fzo1 depletion. Mean of two biological replicates with a total of 200 cells. (**D**) Growth rates were determined in 3h intervals, normalized to the Control at each time interval. Dots indicate data from three biological replicates, bars indicate mean and standard deviation. Source data are available online for this figure.

Since we observed background degradation of Fzo1 using constitutively expressed TIR protein, we expressed TIR under an inducible promoter (using either anhydrotetracycline (aTC) or β-estradiol, see Appendix Table S1 for strains) shortly before depletion. Using this system, depletion of FLAG-AID-Fzo1 is achieved within 5–15 min, and no background degradation is observed before depletion (Fig. 1A).

**Figure EV1:**
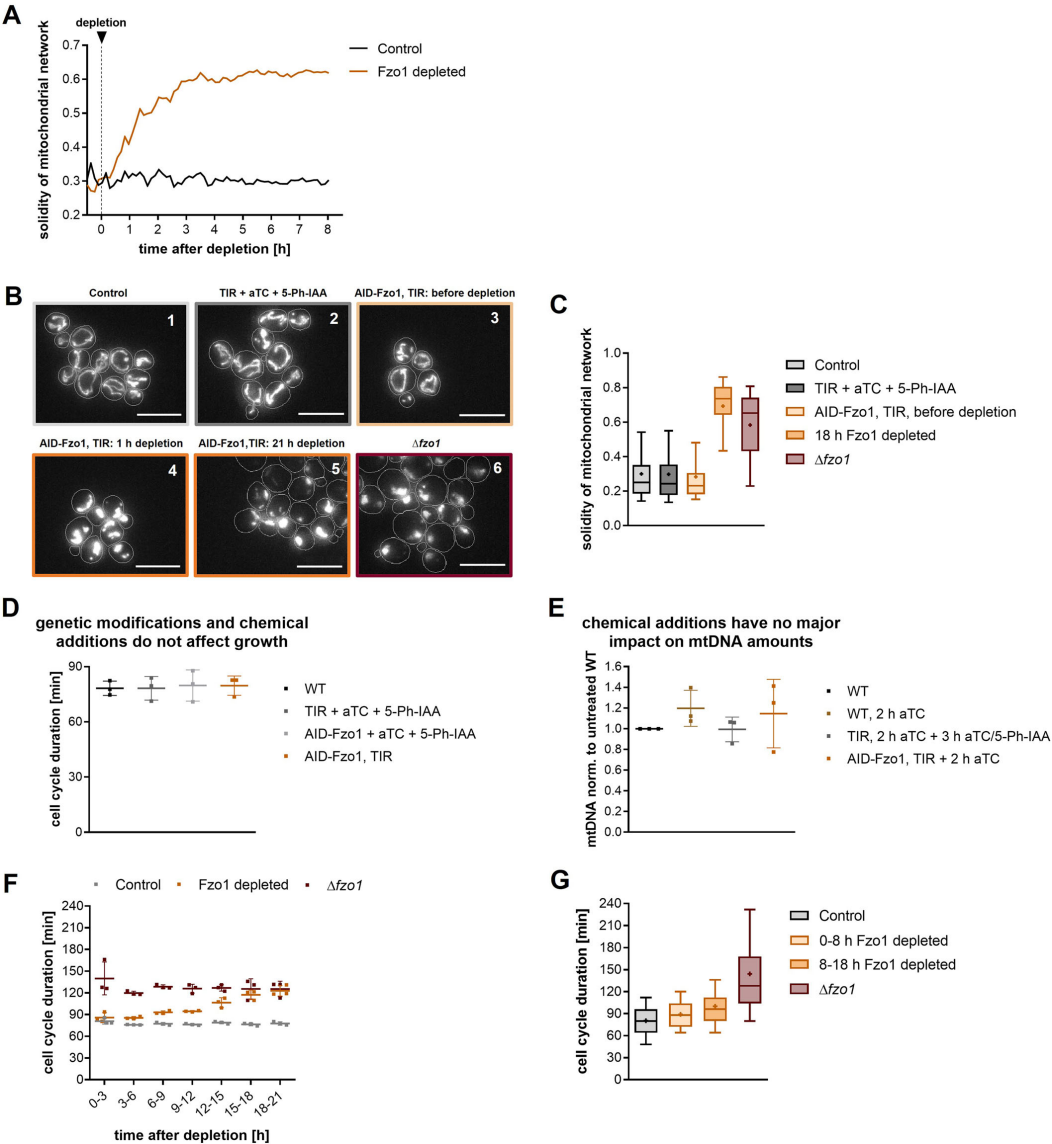
Mitochondrial morphology, mtDNA levels, and growth are not affected by genetic constructs or the addition of chemicals. Cells were grown in synthetic complete (SC) medium containing 1% glucose, and Fzo1 depletion was induced at the indicated times. (**A**) Solidity of the mitochondrial network from epifluorescence microscopy. Mean of three biological replicates with a total of 1694 Control and 1694 Fzo1-depleted cells. (**B**) Example images of maximum z-projections of mitochondria visualized by pre-Su9-mCardinal. Scale bar = 10 µm. 1: Control = AID-Fzo1, TIR, untreated; 2: TIR + anhydrotetracycline (aTC) + 5-Ph-IAA, no AID-Flag; 3: Fzo1-AID, TIR + aTC before addition of 5-Ph-IAA; 4: AID-Fzo1, TIR + aTC + 5-Ph-IAA (1 h 5-Ph-IAA = Fzo1 depletion); 5: Fzo1-AID, TIR + aTC + 5-Ph-IAA (21 h 5-Ph-IAA = Fzo1 depletion); 6: ∆*fzo1*. (**C**) Solidity of different control strains and treatments from three biological replicates. Whiskers indicate 10th and 90th percentiles, + indicates mean, line indicates median, boxes show 25th and 75th percentiles. Control (all 8.5 h of imaging) 26850 datapoints of 1694 cells; TIR + aTC + 5-Ph-IAA (after addition of the chemicals) 17170 datapoints of 1078 cells; Fzo1-AID, TIR, before depletion (0.5 h before addition of 5-Ph-IAA, aTC is added already) 207 datapoints of 57 cells; 18 h Fzo1 depleted 1434 datapoints of 1434 cells; ∆*fzo1* (all of 10 h imaging) 43414 datapoints of 1419 cells. (**D**) Cell cycle duration of WT cells, cells with TIR treated with aTC and 5-Ph-IAA, cells with FLAG-AID-Fzo1 treated with aTC and 5-Ph-IAA, and cells with TIR and FLAG-AID-Fzo1 without treatment. Mean with SD from three biological replicates is shown. (**E**) DNA-qPCR of WT, 2 h aTC-treated WT, TIR-expressing cells treated with aTC and 5-Ph-IAA for 3 h, and cells expressing AID-Fzo1 and TIR treated with aTC for 2 h. mtDNA per nuclear DNA values were normalized to 0 h WT samples. Mean with SD from three biological replicates is shown. (**F**) Unnormalized cell cycle durations corresponding to growth rates shown in Fig. 1D. Mean with SD from three biological replicates is shown. (**G**) Cell cycle durations from live-cell microscopy. Data from three biological replicates is shown. Control: *n* = 1250, 0–8 h Fzo1 depleted: *n* = 1409, 8–18 h Fzo1 depleted: *n* = 1755, ∆*fzo1*: *n* = 1277. Whiskers indicate 10th and 90th percentiles, + indicates mean, line indicates median, boxes show 25th and 75th percentiles. Cell cycle durations from live-cell microscopy are comparable to shake flask cell cycle durations.

Since depletion is achieved rapidly and completely, we can combine this system with microfluidics-based live-cell imaging to precisely determine how rapidly loss of Fzo1 causes fragmentation of the mitochondrial network. To avoid selecting against petite cells, we used synthetic growth medium containing amino acids and 1% glucose (SC-glucose), where cells do not rely on respiration. Mitochondrial morphology was visualized using mCardinal targeted by preSu9, a well-established marker for mitochondria in yeast (Di Bartolomeo et al, 2020; Vowinckel et al, 2015). To quantify alterations of mitochondrial morphology, we segmented mitochondria in 3D and determined their solidity, a measure to describe the compactness of an object (Vőfély et al, 2019). Solidity is high for compact objects like fragmented or clumped mitochondria, while solidity is lower for the more tubular mitochondria of wild-type cells. To determine the timing of mitochondrial morphology changes precisely, we performed confocal microscopy with a frame rate of 30 s. Before depletion, the solidity of the mitochondrial network is very low (~0.2) in agreement with the very tubular morphology of yeast mitochondria (Friedman and Nunnari, 2014). Alterations in mitochondrial morphology are detectable within 20 min after Fzo1 depletion, and fragmentation is almost complete 60 min after Fzo1 depletion (Fig. 1B,C; Movie EV1). In control cells, mitochondrial morphology is maintained throughout the imaged time course (Fig. EV1A). This observation is in line with studies showing that mitochondrial fusion and fission events occur every few minutes (Jakobs et al, 2003; Nunnari et al, 1997; Wisniewski et al, 2024). Disturbing this equilibrium by inhibition of one of the processes consequently results in a fast change of mitochondrial morphology.

We further confirmed that mitochondrial morphology (Fig. EV1B,C), growth (Fig. EV1D), and mtDNA content (Fig. EV1E) are unaffected by TIR expression or the addition of the inducers. Subsequently, we therefore only compared cells bearing TIR and FLAG-AID-Fzo1, which are either depleted or untreated (in the following termed Control).

After showing that mitochondrial morphology changes quickly upon Fzo1 loss, we sought to determine how long it takes wild-type (WT)-like cells to reach the full phenotype of ∆*fzo1* cells, which exhibit a growth defect on glucose media of ~40% compared to Control cells, consistent with previous reports (Shirozu et al, 2016). We found that loss of the Fzo1 protein results in a mildly decreased growth rate already within the first hours after onset of depletion. The decrease continues gradually reaching a growth rate comparable to ∆*fzo1* cells between 18 and 21 h, which corresponds to 10-12 doublings (Figs. 1D and EV1F,G). Thus, while the primary phenotype of Fzo1 loss, fragmentation of mitochondria, is achieved within less than an hour, full reduction in growth is reached only after 21 h.

### Loss of mtDNA and the respiratory chain occur together with alterations of mitochondrial ultrastructure

**Figure 2:**
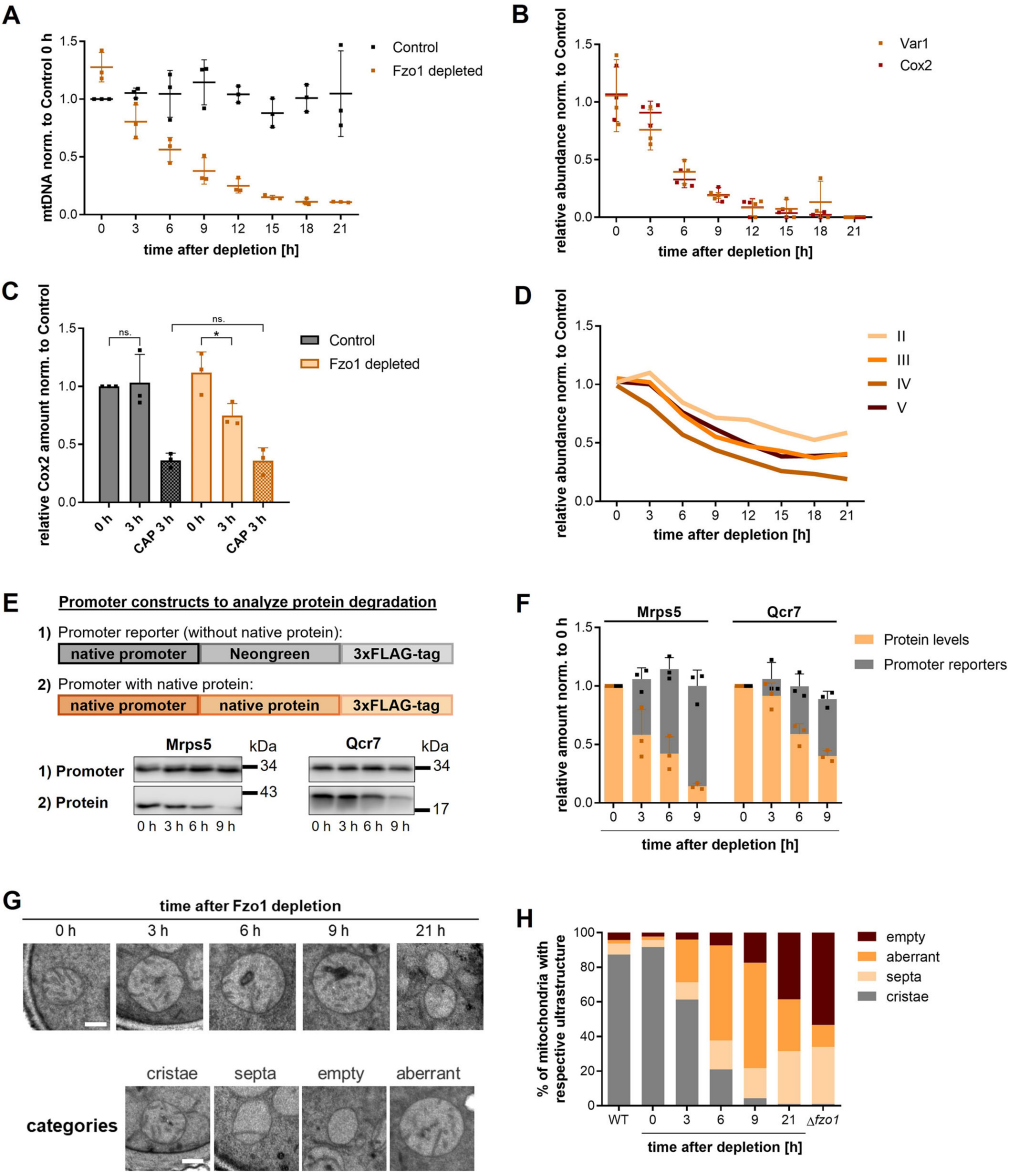
mtDNA is lost progressively after Fzo1 depletion. (**A**) DNA-qPCR of Fzo1-depleted and Control cells. mtDNA per nuclear DNA values were normalized to 0 h Control samples. (**B**) Proteomics measurement of mtDNA encoded Var1 and Cox2. Peptide counts were normalized to 0 h Control samples. (**C**) Quantification of Western blot analysis of Cox2 (see also Fig. EV2). Control and Fzo1-depleted cells were treated with 2 mg/mL chloramphenicol (CAP) to inhibit mitochondrial translation. Statistical significance was determined using an unpaired two-tailed *t* test. 0 h vs 3 h Control: *P* = 0.839, 0 h vs 3 h Fzo1 depleted: *P* = 0.036, 3 h CAP Control vs 3 h CAP Fzo1 depleted: *P* = 0.956. (**D**) Proteomics measurement of nuclear encoded respiratory chain proteins. Lines represent the average of each complex. Abundances were normalized to 0 h Control samples. (**E**) Western blot analysis of 3xFLAG-tagged protein and promoter reporter protein for two nuclear-encoded proteins required for mitochondrial respiration and translation. (**F**) Quantification of western blots shown in (**E**). (**G**) Example images of electron microscopy, see Fig. EV2J for wild-type and ∆*fzo1* images. Scale bar = 200 nm. (**H**) Quantification of electron microscopy images shown in (**G**). At least 150 mitochondria were analyzed for each time point. (**A**–**H**) All measurements are mean values from three biological replicates. (**A**–**C**,**F**) Error bars indicate SD. Source data are available online for this figure.

**Figure EV2:**
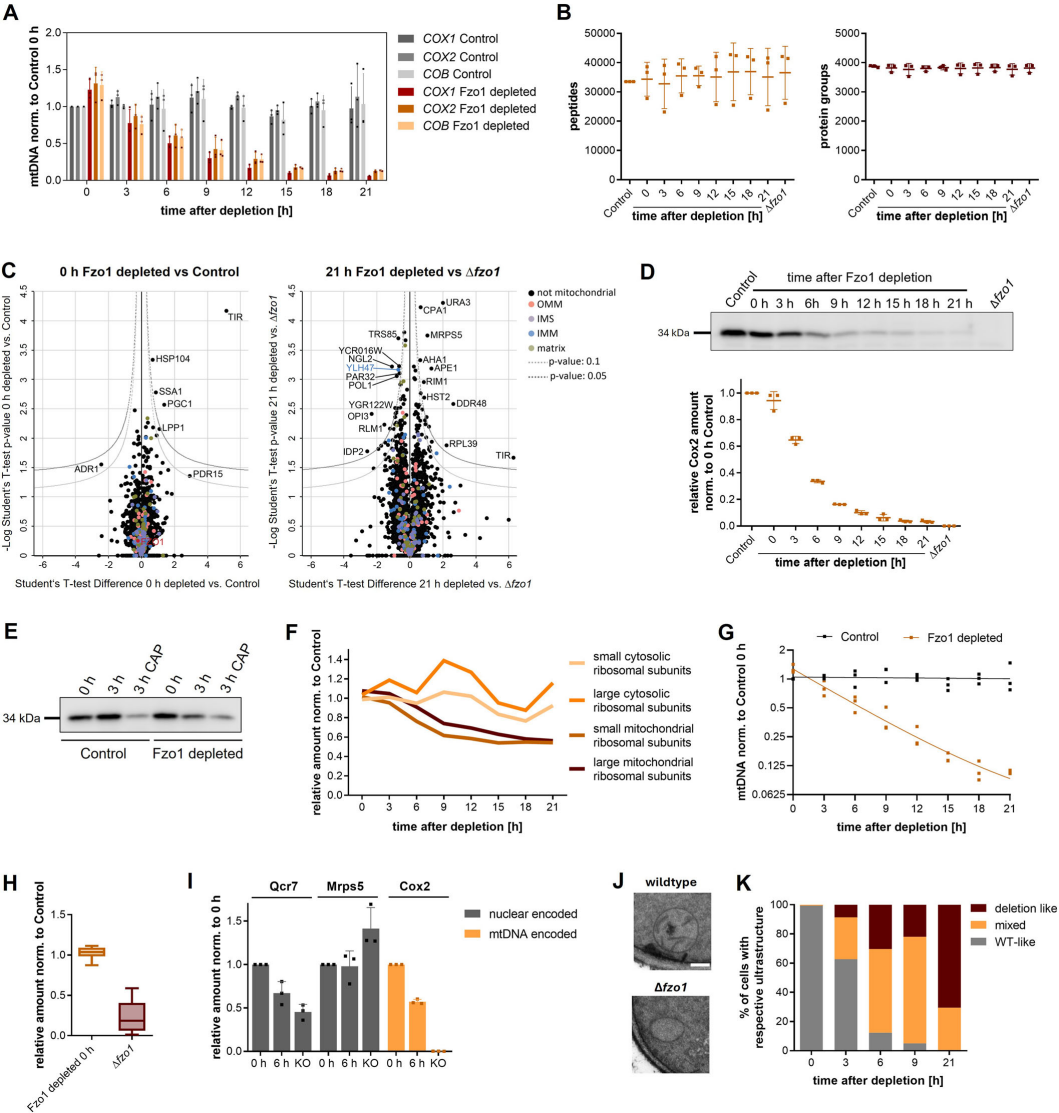
Progressive loss of mtDNA results in the full establishment of the Fzo1 deletion phenotype. (**A**) DNA-qPCR of individual mitochondrial genes. The level of each gene was normalized to the amount of the nuclear gene *ACT1*. Mean and SD from three biological replicates are shown. (**B**) The number of peptides and protein groups detected in the mass spectrometry analysis. Mean with SD from three biological replicates is shown. (**C**) Volcano plots of proteomics measurement of 0 h depleted vs Control cells (left) and 21 h depleted vs ∆*fzo1* cells. 0 h Fzo1 depleted (treated with aTC for 2 h) shows a comparable proteome as Control cells (no aTC). As expected, the strongest and most significant difference is the TIR protein. Data from three biological replicates is shown. (**D**) Example Western blot and quantification of Cox2 levels by. Mean and SD from three biological replicates. (**E**) Example Western blot of Chloramphenicol (CAP) treatment quantified in Fig. 2C. (**F**) Abundance of mitochondrial ribosomal subunits and cytosolic ribosomal subunits measured by mass spectrometry-based proteomics in three replicates, normalized to *t* = 0. The average of all measured protein groups of the respective subunits is shown. (**G**) mtDNA levels shown in Fig. 2A from three biological replicates on log2 scale. Solid lines show a fit to a one-phase exponential decay with a half-life of 4.7 h for Fzo1-depleted cells. (**H**) Abundance of 30 nuclear encoded respiratory chain proteins of Control and ∆*fzo1* cells (related to Fig. 2D) as determined by mass spectrometry from three biological replicates. Whiskers indicate 10th and 90th percentiles, + indicates mean, line indicates median, boxes show 25th and 75th percentiles. (**I**) Expression of nuclear encoded Qcr7 and Mrps5, and mtDNA encoded Cox2 determined by RT-qPCR. Mean and SD from three biological replicates with three technical replicates each are shown. (**J**) Example pictures of electron microscopy related to Fig. 2H), scale bar = 200 nm. (**K**) Categorization of cells based on their ultrastructure in electron microscopy of three biological replicates, related to Fig. 2H.

To understand the relationship between growth reduction and mtDNA loss after Fzo1 depletion, we determined mtDNA levels relative to nuclear DNA using quantitative DNA-PCR (qPCR). The depletion of Fzo1 leads to a severe loss of mtDNA within a few generations (Fig. 2A), following an exponential decline with a half-life of approximately 4.5 h (Fig. EV2G). We compared the levels of the different coding regions of the mitochondrial genome that had been described to be differentially lost upon ROS stress (Stenberg et al, 2022). However, we could not see differences between them (Fig. EV2A), suggesting that there is no loss of mtDNA segments prior to the complete loss of mtDNA.

We next wondered how rapidly mtDNA-encoded proteins are lost. mtDNA in yeast encodes four subunits of the respiratory chain, three subunits of the ATP synthase (complex V), and one subunit of the mitochondrial ribosome. To analyze how fast the levels of these and other mitochondrial proteins decline, we performed mass-spectrometry-based proteomics of the same time course as described above (Dataset EV1). Numbers of detected peptides and protein groups were comparable for all time points (Fig. EV2B). We first confirmed that upon start of the Fzo1 depletion (0 h), cells have a comparable proteome as Control cells in which TIR is not expressed, and that cells after 21 h of Fzo1 depletion have a proteome that is comparable to ∆*fzo1* cells (Fig. EV2C).

Of the mtDNA-encoded proteins, Cox2 and Var1 were measured with high reproducibility across time points. Interestingly, the dynamics with which the abundance of Cox2 and Var1 proteins decreases was similar to the rate of mtDNA loss (Fig. 2B), which we also confirmed by Western blot analysis of Cox2 (Fig. EV2D). Since there was no delay between the decline of mtDNA and mtDNA encoded proteins, we investigated if mtDNA encoded proteins are actively degraded. We inhibited mitochondrial translation using chloramphenicol (CAP) in Control and Fzo1-depleted cells, in a Δ*pdr5* deletion strain which is defective in CAP export from the cell, thereby allowing efficient translational inhibition (Leonard et al, 1994; Roussou et al, 2024). As in previous experiments, we detected a decrease in Cox2 abundance 3 h after Fzo1 depletion (Fig. 2C). Inhibition of mitochondrial translation resulted in a strong decrease of Cox2 in Control cells, which correlates with dilution through growth. Fzo1-depleted cells treated with CAP showed a similar decrease in Cox2 abundance (Figs. 2C and EV2E). This indicates that active degradation of mtDNA encoded proteins does not play a major role in the loss of mtDNA encoded proteins, but instead their synthesis is strongly reduced. Consistent with this, Cox2 mRNA is also substantially decreased 6 h after Fzo1 depletion (Fig. EV2I). A reduction of synthesis of mitochondrial encoded proteins is further supported by the observation that mitochondrial ribosomal proteins are also reduced, while cytosolic ribosomal proteins were not reduced (Fig. EV2F).

The levels of nuclear-encoded respiratory chain proteins are known to be coupled to the presence of mtDNA-encoded respiratory chain proteins (Dagsgaard et al, 2001; Vowinckel et al, 2021). In line with this, most nuclear encoded subunits of the respiratory chain complexes also decrease after loss of Fzo1 (Fig. 2D), although with a slight delay compared to mtDNA encoded subunits (Fig. 2B). Our proteomics analysis confirms that most of the nuclear encoded respiratory chain proteins are strongly reduced in ∆*fzo1* cells (Fig. EV2H).

We next sought to investigate whether the reduction in nuclear-encoded respiratory chain and mitochondrial ribosomal proteins is due to decreased synthesis through retrograde signaling or whether they are synthesized at a constant rate and subsequently degraded. We first confirmed by Western blot that two representative endogenously Flag-tagged proteins, Mrps5 and Qcr7, decrease. We then constructed reporters consisting of 800 bp of the respective promoter regions of these proteins driving the expression of Neongreen-FLAG (Fig. 2E). Since these reporters only consist of the fluorophore and FLAG-tag but lack a mitochondria targeting sequence they are only present in the cytosol, thus reporting only on transcriptional, not on posttranslational regulation. While the abundance of the proteins (Fig. 2E,F) is reduced by 60–80% within the first 9 h after depletion, the abundance of the promoter-Neongreen constructs is stable (Fig. 2E,F). To substantiate these results, we also measured mRNA levels of Mrps5 and Qcr7 by RT-qPCR. In line with our fluorescent reporter, Mrps5 RNA concentrations remained stable 6 h after Fzo1-depletion (Fig. EV2I), even though the protein strongly declined, confirming that protein levels of Mrsp5 are controlled posttranscriptionally, likely through degradation. Surprisingly, the RNA of Qcr7 behaved differently. This RNA decreased in concentration to a similar extent as the protein already at 6 h. This suggests complex regulation on transcriptional or RNA-stability level that is not captured by the promoter sequence alone. This warrants further investigation in future studies to understand the complex coordination of nuclear- and mitochondrially encoded OXPHOS subunits.

The ATP synthase is composed of nuclear- and mitochondria-encoded subunits and is strongly reduced upon Fzo1 depletion (complex V, Fig. 2D). The formation of ATP synthase dimers is required for normal cristae structure (Klecker and Westermann, 2021; Paumard et al, 2002). In line with this, we observe changes in mitochondrial ultrastructure after Fzo1 depletion (Fig. 2G,H). In the first 3 h following depletion, most mitochondria still have cristae. However, 6 and 9 h after Fzo1 depletion, most mitochondria have aberrant ultrastructure and 21 h after depletion most mitochondria resemble the Δ*fzo1* deletion phenotype, where mitochondria show a complete loss of normal cristae (Fig. 2G,H, see Fig. EV2J for wild-type and ∆*fzo1* examples). During the first 9 h of depletion, many cells have a mixture of WT and deletion-like mitochondria (Fig. EV2K), suggesting that loss of functionality and likely mtDNA is not homogenous, not even within individual cells.

### Fzo1-depleted cells exhibit a large variation in mitochondrial mass and functionality

**Figure EV3:**
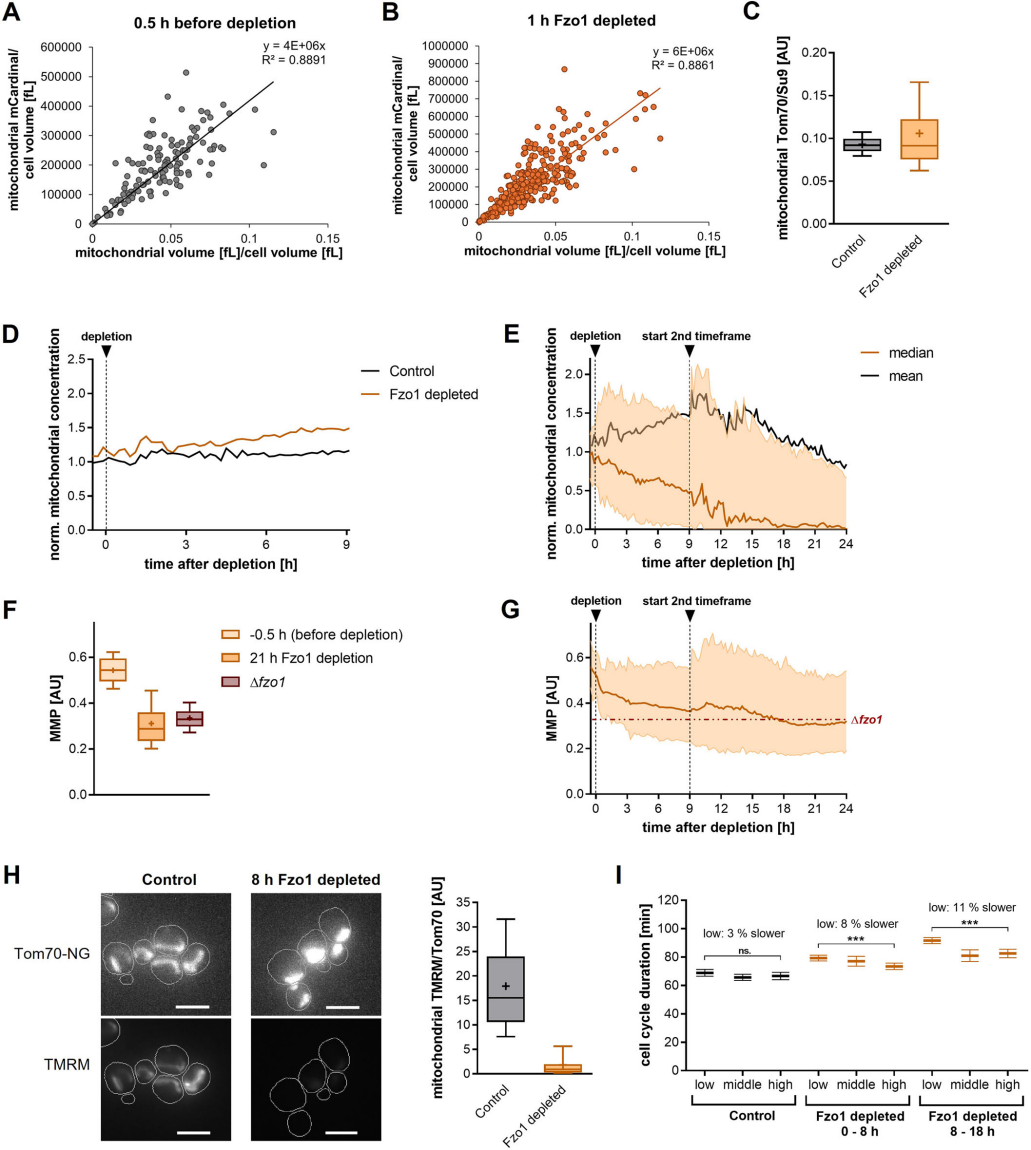
Quantification of mitochondrial concentration and mitochondrial membrane potential by microscopy. (**A**, **B**) Mitochondrial volume estimated from a 3D volume reconstruction was compared to mitochondrial volume estimated from total pre-Su9-mCardinal fluorescence intensity. mCardinal signal correlates with mitochondrial volume/cell volume. However, due to the morphology change the slope of the correlation changes, likely to bias in the 3D reconstruction. Therefore, we consider the mitochondrial mCardinal as the better proxy for mitochondrial concentration. (**A**) mitochondrial volume [fL]/ cell volume [fL] vs mitochondrial mCardinal/cell volume [fL] before depletion. *n* = 129. (**B**) mitochondrial volume [fL]/cell volume [fL] vs mitochondrial mCardinal/cell volume [fL] 1 h after Fzo1 depletion. *N* = 286. (**C**) Mitochondrial Tom70-Neongreen/preSu9-mCardinal for Control and Fzo1-depleted cells 8 h after depletion. n (Control) = 127, *n* (Fzo1 depletion) = 165. Whiskers indicate 10th and 90th percentiles, + indicates mean, line indicates median, boxes show 25th and 75th percentiles. (**D**) Mean of the normalized mitochondrial concentrations after Fzo1 depletion. Same data as in Fig. 3A. (**E**) Normalized mitochondrial concentrations after Fzo1 depletion. Shaded areas show 25th and 75th percentiles. Cells were imaged in two time intervals: −0.5–9 h and 9–24 h after Fzo1 depletion. *n* (24 h Fzo1 depletion) = 2019. (**F**) MMP of controls as described in Fig. 3. −0.5 h before depletion: 184 datapoints of 67 cells, 21 h Fzo1 depletion: 718 datapoints of 718 cells, ∆*fzo1:* 25,197 datapoints of 1376 cells over 10 h of imaging. Whiskers indicate 10th and 90th percentiles, + indicates mean, line indicates median, boxes show 25th and 75th percentiles. (**G**) MMP of Fzo1-depleted cells shown in (**E**). Shaded areas show 5th and 95th percentiles. *n* (24 h Fzo1 depletion) = 2019. The dashed line depicts the median MMP of ∆*fzo1* cells. (**H**) TMRM staining of Control and 8 h Fzo1-depleted cells. Left: example images of maximum z-projections, scale bar = 5 µm. Right: Quantification of the TMRM staining: mitochondria were segmented using Tom70-Neongreen, and the TMRM signal was normalized to the Tom70 signal. *n* (Control) = 290, *n* (Fzo1 depletion) = 189. Whiskers indicate 10th and 90th percentiles, + indicates mean, line indicates median, boxes show 25th and 75th percentiles. (**I**) Cell cycle duration of generation >1 with different mitochondrial concentrations. Categorization was performed based on Control cells gen >1. Images were taken every 8 min to determine cell cycle durations. Mean with 95% Confidence interval is shown. Statistical significance was determined using a paired two-tailed *t* test. Control low vs high: *P* = 0.219, 0–8 h Fzo1 depleted: *P* = 0.0005, 8–18 h Fzo1 depleted: *P* < 0.0001. Control: *n* (low) = 233, *n* (middle) = 233, *n* (high) = 240; Fzo1 depleted 0–8 h: *n* (low) = 460, *n* (middle) = 98, *n* (high) = 232; Fzo1 depleted 8–18 h: *n* (low) = 714, *n* (middle) = 82, *n* (high) =  232.

Since respiratory chain proteins were strongly reduced, we next investigated whether mitochondrial content in the cell is generally affected after Fzo1 depletion. Mitochondrial biosynthesis in WT cells is regulated with cell size, which leads to a roughly constant concentration of mitochondria (total volume of mitochondria per cell volume) in the population (Rafelski et al, 2012; Seel et al, 2023). Three-dimensional volume reconstruction of mitochondria may be biased by the different shapes of WT and fragmented mitochondria. Therefore, we instead used the total fluorescence signal intensity derived from mitochondrial-imported preSu9-mCardinal and normalized it to the cell volume as a robust approximation of mitochondrial concentration (in the following termed mitochondrial concentration, see also “Methods” and Fig. EV3A,B for controls). We confirmed that the total fluorescence intensity of mitochondrially imported preSu9-mCardinal correlates with the total fluorescence intensity derived from the outer membrane protein Tom70-Neongreen, a marker also often used for quantifying mitochondrial content (Fig. EV3C, (Higuchi-Sanabria et al, 2016; Hughes and Gottschling, 2012; Pringle et al, 1989)).

**Figure 3:**
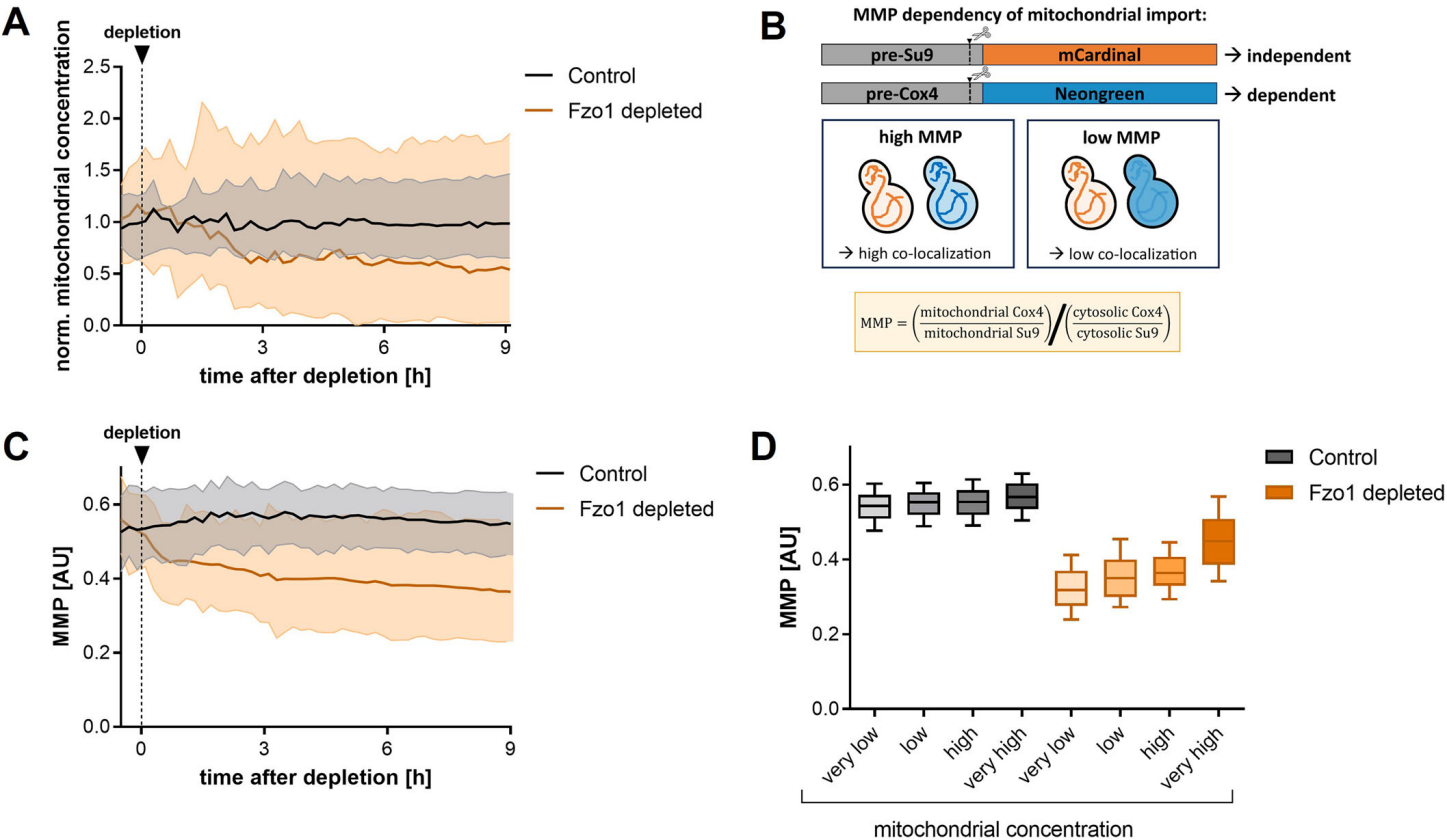
Fzo1-depleted cells exhibit large variation in mitochondrial concentration and functionality. (**A**) Normalized mitochondrial concentration (total mitochondrial signal intensity per cell volume). Depletion is induced at *t* = 0. Median with 25th and 75th percentiles from two biological replicates with a total of 1785 Control and 2075 Fzo1-depleted cells is shown. The variability of the spreads at 9 h differs significantly between groups (Brown–Forsythe test, *P* = 6.78 × 10^−46^; IQR: Control = 0.81, Fzo1 depletion = 1.82), see also Appendix Fig. S2A. (**B**) Schematic of the reporter for estimating mitochondrial membrane potential (MMP). Mitochondrial import of pre-Su9 is mostly independent of the MMP, while import of pre-Cox4 is dependent on the MMP. (**C**) Estimation of the MMP after Fzo1 depletion. Average with 5th and 95th percentiles from two biological replicates with a total of 1723 Control and 1595 Fzo1-depleted cells is shown. The variability of the spreads at 9 h differs significantly between groups (Brown–Forsythe test, *P* = 7.80 × 10^−93^; IQR: Control = 0.066, Fzo1 depletion = 0.129). (**D**) MMP of cells with different mitochondrial concentrations of all G1 cells 0–9 h after Fzo1 depletion. Categories were determined by the quartiles of the mitochondrial concentrations of Control cells. Boxes indicate 25th percentile, median, and 75th percentile. Whiskers indicate 10th and 90th percentiles. Control cells: 407 cells per category; Fzo1 depleted cells: *n* (very low) = 821, *n* (low) =  184, *n* (high) = 183, *n* (very high) = 581. Distributions differ significantly across the mitochondrial concentrations under a Gamma likelihood-ratio test for both Control (*P* = 3.513 × 10^−12^) and Fzo1-depleted cells (*P* < 2.225 × 10^−^³⁰⁸). Medians increase significantly from the very low to very high mitochondrial concentrations (Wilcoxon rank-sum tests, Control: *P* = 2.152 × 10^−13^, Fzo1-depleted cells: *P* = 1.935 × 10^−67^), with the very high group being 1.04-fold and 1.35-fold higher than the very low group in Control and Fzo1-depleted cells, respectively. Source data are available online for this figure.

We show that the mean of the mitochondrial concentration of the population does not decrease within the first 9 h after Fzo1 depletion (Fig. EV3D; Appendix Fig. S1). However, the single-cell distribution of mitochondrial concentration broadens substantially (Fig. 3A; Appendix Fig. S2), and at the same time becomes less normally distributed. This results in a decrease of the median mitochondrial concentration approximately 1 h after depletion (Fig. 3A), leading to a large fraction of the population with an extremely low mitochondrial concentration as depicted by the 25th percentile of the population (Fig. 3A). This becomes even more pronounced if Fzo1 is depleted for a prolonged period of time (Fig. EV3E).

Since WT cells maintain relatively constant mitochondrial concentrations (Rafelski et al, 2012; Seel et al, 2023), we investigated if this large variability of mitochondrial concentrations after Fzo1 depletion affects mitochondrial function, such as the mitochondrial membrane potential (MMP). To estimate MMP, we used the Mitoloc system (Vowinckel et al, 2015), which consists of two fluorescent reporters: one containing the Su9 presequence whose import into mitochondria is largely independent of the MMP, and one containing the Cox4 presequence whose import is strongly dependent on the MMP (Fig. 3B). The ratio of the two reporters in the mitochondria thus allows an approximation of the MMP. We improved the signal-to-noise of the original construct by integrating the reporter into the nuclear genome and expressing both fluorophores from the same strong constitutive promoter. Before Fzo1 depletion, the MMP is comparable to that of control cells. Following the MMP over time revealed that the MMP shows an immediate drop after Fzo1 depletion, simultaneously with the mitochondrial network fragmentation. The MMP then decreases further within the first 9 h of depletion and reaches a deletion-like level between 21 and 24 h (Fig. EV3F,G), while Control cells maintain a stable MMP. We further confirmed the strong decrease of the MMP within the first 8–9 h with TMRM, a MMP-sensitive dye, which shows minimal accumulation in mitochondria 8 h after Fzo1 depletion (Fig. EV3H). Interestingly, the MMP of Fzo1-depleted cells also shows an increased variability between individual cells, indicating a large variation in mitochondrial function (Fig. 3C) that is in line with the observed heterogeneity in ultrastructure.

Since we detected a broadened distribution for both mitochondrial concentration and MMP, we checked whether there is a correlation between mitochondrial concentration and MMP. We grouped G1 Control cells based on their mitochondrial concentration into quartiles. Control cells show very similar MMP values independent of their mitochondrial concentration. In contrast, using the same categories determined from Control cells, we find that Fzo1-depleted cells show large differences in the MMP depending on their mitochondrial concentration. Cells with low mitochondrial concentrations exhibit a much lower MMP than those with high mitochondrial concentrations (Fig. 3D). Mitochondrial health is known to be important for cellular health. In line with this, we found that cells with less mitochondrial content exhibit extended division times compared to those with higher mitochondrial concentrations (Fig. EV3H). This is most strongly pronounced during later times of depletion (8–18 h).

### Fzo1-depleted cells exhibit mitochondrial inheritance defects

**Figure 4:**
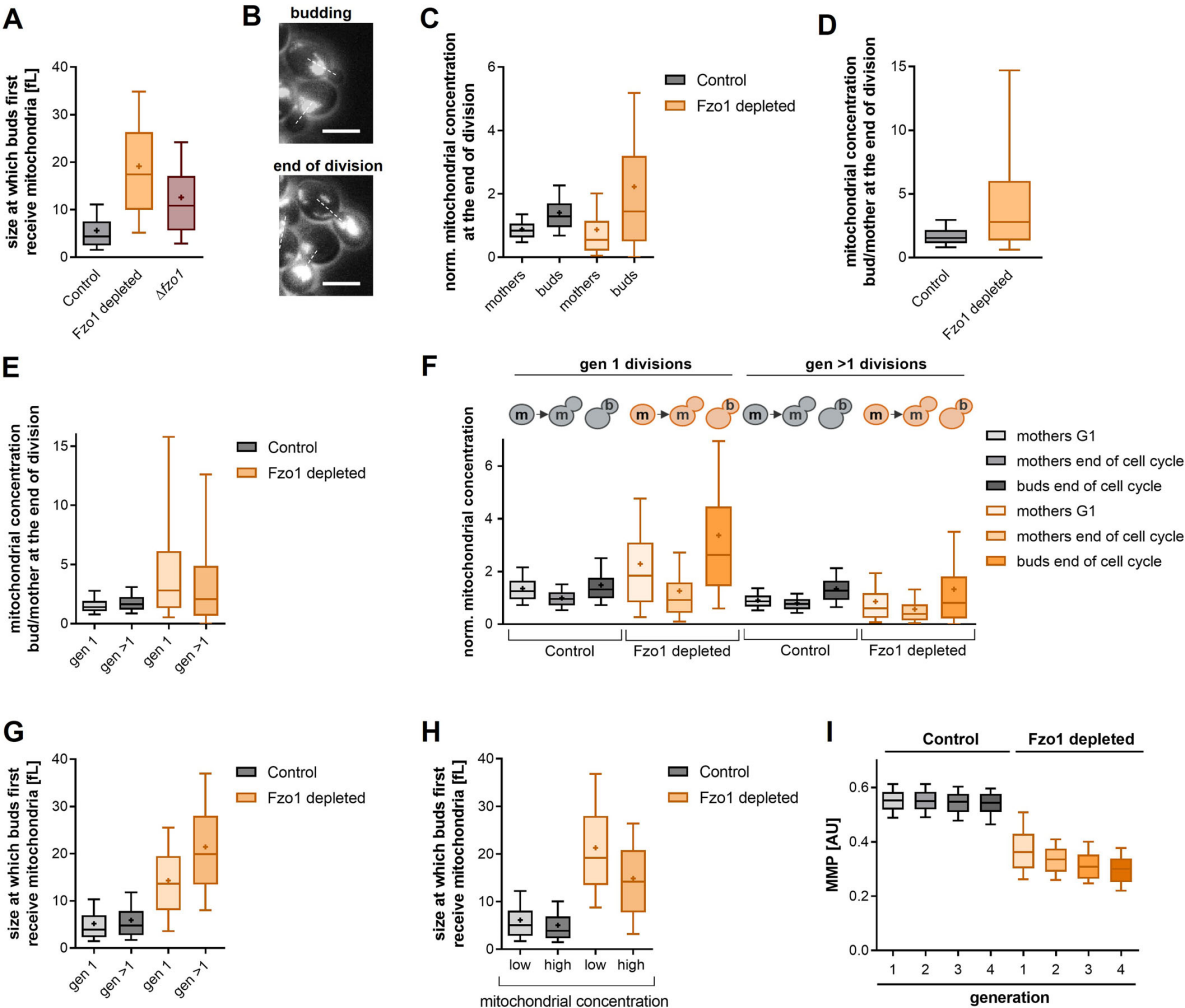
Fzo1-depleted cells exhibit mitochondrial inheritance defects. (**A**) Cell size at which buds first receive mitochondria. Images were taken every 8 min and the time preSu9-mCardinal was first detected in the bud was determined. Control = 1249, Fzo1 depleted = 1275, ∆*fzo1* = 1237. (**B**) Example images as maximum z-projections of mitochondria after Fzo1 depletion during budding and at the end of the cell cycle. Scale bar = 5 µm. (**C**) Normalized mitochondrial concentration (total mitochondrial signal intensity per cell volume) at the last recorded timepoint before division. *n* (Control) = 1250, *n* (Fzo1 depleted) = 1409. The variability of the spreads differs significantly between Control and Fzo1 depletion mothers (Brown–Forsythe test, *P* = 3.516 × 10^−40^; IQR: Control = 0.44, Fzo1 depletion = 0.94) and Control and Fzo1 depletion buds (Brown–Forsythe test, *P* = 1.228 × 10^−75^; IQR: Control = 0.75, Fzo1 depletion = 2.69). (**D**) Ratio of the mitochondrial concentration of buds to mothers at the end of the cell cycle of cells shown in (**C**). The variability of the spreads differs significantly between Control and Fzo1 depletion (Brown–Forsythe test, *P* = 6.802 × 10^−16^; IQR: Control = 1.02, Fzo1 depletion = 4.67). (**E**) Ratio as in (**D**) for gen 1 and gen >1. Control: *n* (gen 1) = 544, *n* (gen >1) = 706; Fzo1 depleted: *n* (gen 1) = 586, *n* (gen >1) = 735. (**F**) Mitochondrial concentrations of mothers and buds through the cell cycle of gen 1 and gen >1 divisions. Number of cells as in (**E**). (**G**) The size at which buds of gen 1 or gen >1 mothers first receive mitochondria. Number of cells as in (**E**). (**H**) The size at which buds of mothers with low or high mitochondrial concentrations first receive mitochondria. The median mitochondrial concentration of Control cells at G1 was used to determine low and high categories. Control: *n* (low) = 625, *n* (high) = 624; Fzo1 depleted: *n* (low) = 638, *n* (high) = 637. (**I**) Estimation of MMP as described in Fig. 3 of generation 1–4 mothers at the beginning of the cell cycle. Control: *n* (gen 1) = 1478, *n* (gen 2) = 383, *n* (gen 3) = 215, *n* (gen 4) = 114; Fzo1 depleted: *n* (gen 1) = 1550, *n* (gen 2) = 406, *n* (gen 3) = 220, *n* (gen 4) = 102. See Fig. EV4E for mitochondrial concentrations. Distributions differ significantly across generations under a Gamma likelihood-ratio test (*P* = 0.0160 for Control, *P* < 2.225 × 10^−^³⁰⁸ for Fzo1-depleted cells). Medians decrease significantly from the first to fourth generation (Wilcoxon rank-sum tests, *P* = 0.0191 for Control and *P* = 1.708 × 10^−15^ for Fzo1 depleted) with the first generation being 1.02-fold and 1.21-fold higher than the fourth generation in Control and Fzo1 depleted cells, respectively. (**A**–**I**) Whiskers of box plots indicate 10th and 90th percentiles, line indicates median, boxes show 25th and 75th percentiles. Where shown, + indicate means. Source data are available online for this figure.

**Figure EV4:**
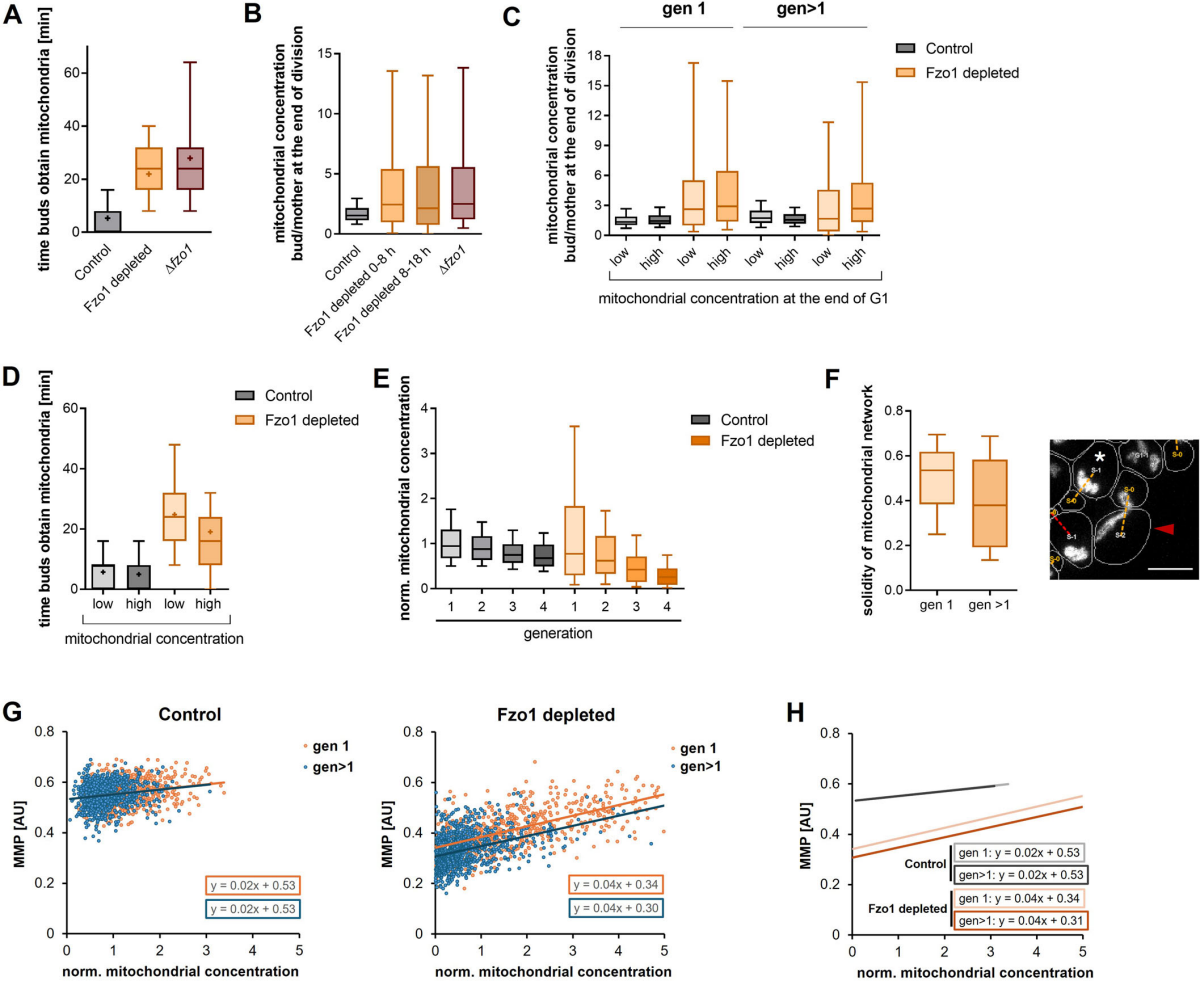
First-generation cells with higher mitochondrial concentrations exhibit a higher mitochondrial membrane potential and earlier mitochondrial inheritance. (**A**) Time when mitochondria are first detected in the bud. Control = 1249, Fzo1 depleted = 1275, ∆*fzo1* = 1237. (**B**) Ratio of the mitochondrial concentration of buds to mothers at the end of the cell cycle. *n* (Control) = 1250, *n* (Fzo1 depleted 0–8 h) = 1324, *n* (Fzo1 depleted 8–18 h) = 1410, *n* (∆*fzo1*) = 1226. (**C**) Ratio of the mitochondrial concentration of buds to mothers at the end of the cell cycle, sorted by the mitochondrial concentration at the end of G1. Grouping is based on the quartiles of the Control. gen 1 (Control): *n* (low) =  272, *n* (high) = 272; gen 1 (Fzo1 depleted): *n* (low) = 164, *n* (high) = 425; gen > 1 (Control): *n* (low) = 353, *n* (high) = 353; gen > 1 (Fzo1 depleted): *n* (low) = 473, *n* (high) = 262. (**D**) The time when mitochondria are first detected in the bud of mothers with a low or high mitochondrial concentration. The median mitochondrial concentration of Control cells at G1 was used to determine low and high categories. Control: *n* (low) = 625, *n* (high) = 624; Fzo1 depleted: *n* (low) = 638, *n* (high) = 637. (**E**) Mitochondrial concentrations of cells shown in Fig. 4I. Distributions differ significantly across generations under a Gamma likelihood-ratio test (*P* < 2.225 × 10^−^³⁰⁸). Medians decrease strongly and significantly for Control and Fzo1-depleted cells from the first to fourth generation (Wilcoxon rank-sum tests, *P* = 8.456 × 10^−11^ for Control and *P* = 3.231 × 10^−20^ for Fzo1-depleted cells) with the median in the first generation being 1.4-fold and 3.1-fold higher than the fourth generation in Control and Fzo1-depleted cells, respectively. (**F**) Solidity of gen 1 and gen >1 mothers throughout their cell cycle is shown. *n* > 11,000 datapoints of at least 257 cells each. Red arrow on the example image shows a gen > 1 mother, white asterisk shows a first-generation mother. Scale bar = 5 µm. The difference in mitochondrial morphology is likely one factor that contributes to the lower inheritance of mitochondria to buds in higher generations. In higher generation cell cycles the mitochondrial network of the mothers more likely exhibits strings as observed by a lower solidity than in first-generation divisions. Of note, these are only transient morphological changes and are not a sign of incomplete Fzo1 depletion in these mitochondria. (**G**) MMP vs mitochondrial concentration of mothers of gen 1 or gen > 1 for Control and Fzo1-depleted cells (0–9 h following Fzo1 depletion). Control: *n* (gen 1) = 687, *n* (gen >1) = 943. Fzo1 depleted: *n* (gen 1) = 745, *n* (gen >1) = 1024. (**H**) Slopes of the data shown in (**G**). (**A**–**F**) Whiskers of box plots indicate 10th and 90th percentiles, line indicates median, boxes show 25th and 75th percentiles. Where shown, + indicate means.

Having shown that Fzo1 depletion leads to a large variability in mitochondrial concentration (total mitochondrial signal intensity per cell volume), which correlates with mitochondrial MMP, we investigated which cells obtain a low mitochondrial concentration. Since it was known that ∆*fzo1* deletion cells exhibit delayed mitochondrial inheritance (Böckler et al, 2017), we analyzed if buds inherit fewer mitochondria in Fzo1-depleted cells. We determined the size at which buds first receive mitochondria in ∆*fzo1* and Fzo1-depleted cells. ∆*fzo1* and Fzo1-depleted buds receive mitochondria on average at a larger size and at later timepoints than Control cells (Figs. 4A,B and EV4A), confirming observations from Böckler et al.

However, when following mitochondria to the end of the cell cycle, a different picture emerged. Immediately before division, a much higher mitochondrial concentration was detected in buds compared to mothers (Fig. 4B,C). We determined the ratio of mitochondrial concentration of each bud in relation to its mother at the end of the cell cycle. Fzo1-depleted cells show a 60% higher median ratio and a higher variability of this ratio compared to Control cells. (Fig. 4D). This was also observed when Fzo1 was depleted for prolonged periods of time and in ∆*fzo1* cells (Fig. EV4B). Thus, even though buds of Fzo1-depleted cells receive mitochondria later than buds of Control cells, they often receive a much higher mitochondrial concentration at the end of the cycle.

Given that at division most buds have a higher concentration of mitochondria than their mothers, we wondered how this would affect mitochondria during the following cell cycles. After division, the bud becomes a ‘first-generation mother’, while its mother continues with its next cell cycle. In first-generation mothers, Fzo1-depleted cells start off with a higher mitochondrial concentration than first-generation Control cells (Fig. 4F; Appendix Fig. S2). However, these first-generation mothers then mostly transfer more mitochondria to their bud than they retain, resulting in a higher mitochondrial concentration ratio between the bud and mother at the end of the cell cycle and a strong reduction of the mitochondrial concentration of the mother compared to Control cells (Fig. 4E,F).

In higher-generation divisions, where the mother typically already starts off with a lower mitochondrial concentration, the median ratio of the mitochondrial concentration between bud and mother is lower than in first-generation divisions but still higher than in Control cells. However, in these higher-generation divisions, the distribution of the ratios shifts, so that in some divisions, the buds obtain a very low mitochondrial concentration (Fig. 4E,F). We observed that for both first and higher generation divisions, the ratio of the mitochondrial concentration between bud and mother is higher if the mother has a high mitochondrial concentration at the end of G1 (Fig. EV4C). We therefore asked if buds of higher generation divisions obtain little mitochondria due to the lower mitochondrial concentration of their mothers. Indeed, buds of higher generation mothers, which often have a low mitochondrial concentration compared to first-generation mothers, obtain mitochondria at a larger size (Fig. 4G,H) and at a later time (Fig. EV4D).

In line with a higher mitochondrial concentration in first-generation cells compared to higher-generation cells (Fig. EV4E), we measured a higher MMP in first-generation cells than in higher-generation cells (Fig. 4I). Even at the same mitochondrial concentration, first-generation cells have a higher MMP. While this effect can be clearly seen in Fzo1-depleted cells, Control cells have very similar MMP values throughout generations (Fig. EV4G,H). This indicates that not only a large amount, but also high-quality mitochondria are preferentially transferred into buds which will then become first-generation mothers, and that mothers are left with a low amount of low-quality mitochondria.

Taken together, our results show that despite the delayed inheritance of mitochondria, buds obtain a large proportion of mitochondria at division. This causes a strongly unequal distribution of mitochondrial mass within the population, which in turn affects MMP and likely functionality.

### mtDNA-encoded proteins are also segregated unequally during division

**Figure EV5:**
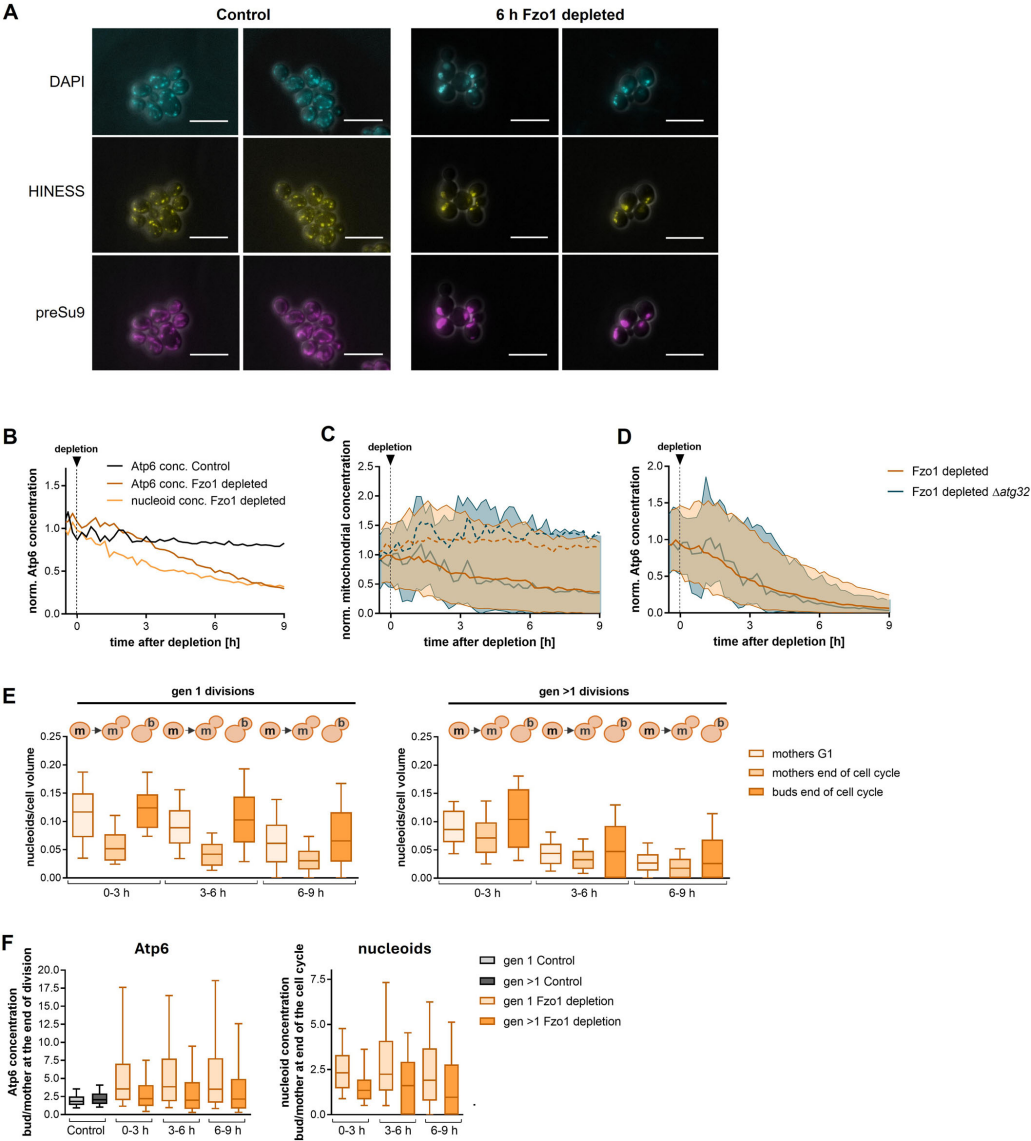
Atp6 concentration is a good proxy for nucleoid counts. (**A**) Example images of DAPI staining compared to mt-Kaede-HI-NESS of Control and Fzo1-depleted cells 6 h after depletion. Scale bar = 10 µm. (**B**) Atp6 and nucleoid concentration after Fzo1 depletion. Same data as in main Fig. 5A,B. (**C**) Mitochondrial concentration after Fzo1 depletion in ∆*atg32* cells which are unable to perform mitophagy. Mean (dashed lines) and median (solid lines) with 25th and 75th percentiles are shown for Fzo1-depleted cells without additional mutation (orange) and for Fzo1-depleted cells with Atg32 deletion (turquoise). *n* (Fzo1 depletion at 9 h) = 3686 cells from three biological replicates, *n* (Fzo1 depletion with ∆*atg32* at 9 h) = 1909 cells from two biological replicates. (**D**) Atp6-Neongreen concentration of data shown in (**C**). (**E**) Nucleoid concentrations of mothers at the beginning of the cell cycle (G1), and of mothers and buds at the end of the cell cycle. *n* (gen 1): 0–3 h = 48, 3–6 h = 225, 6–9 h = 848; *n* (gen >1): 0–3 h = 71, 3–6 h = 303, 6–9 h = 1111. Whiskers indicate 10th and 90th percentiles, line indicates median, boxes show 25th and 75th percentiles. (**F**) Ratio of the Atp6 and nucleoid concentrations between buds and mothers at the end of division. Atp6 (corresponding to data shown in Fig. 5C) *n* (gen 1): Control = 1095, 0–3 h = 569, 3–6 h = 2122, 6–9 h = 728; *n* (gen >1): Control = 1389, 0–3 h = 777, 3–6 h = 2293, 6–9 h = 789. Nucleoid concentration (corresponding to data shown in (**E**) *n* (gen 1): 0–3 h = 47, 3–6 h = 210, 6–9 h = 682; *n* (gen >1): 0–3 h = 70, 3–6 h = 274, 6–9 h = 801. Whiskers indicate 10th and 90th percentiles, line indicates median, boxes show 25th and 75th percentiles.

Having shown that mitochondrial mass is strongly unequally distributed through cell division with effects on mitochondrial functionality, we investigated how this affects mtDNA and mtDNA-encoded proteins. To monitor mtDNA in live cells, we used the recently established mt-Kaede-HI-NESS system, which fluorescently labels nucleoids based on a bacterial DNA-binding protein (preprint: Deng et al, 2025), see Appendix Table S3 for plasmid details, see Fig. EV5A for comparison with DAPI staining. In another strain, we integrated the degradable Fzo1 into a previously established strain in which Atp6, a mtDNA-encoded subunit of the ATP synthase, is tagged with mNeongreen in the mitochondrial genome (Jakubke et al, 2021). This was shown previously to be a good proxy for functional mtDNA (Roussou et al, 2024).

**Figure 5:**
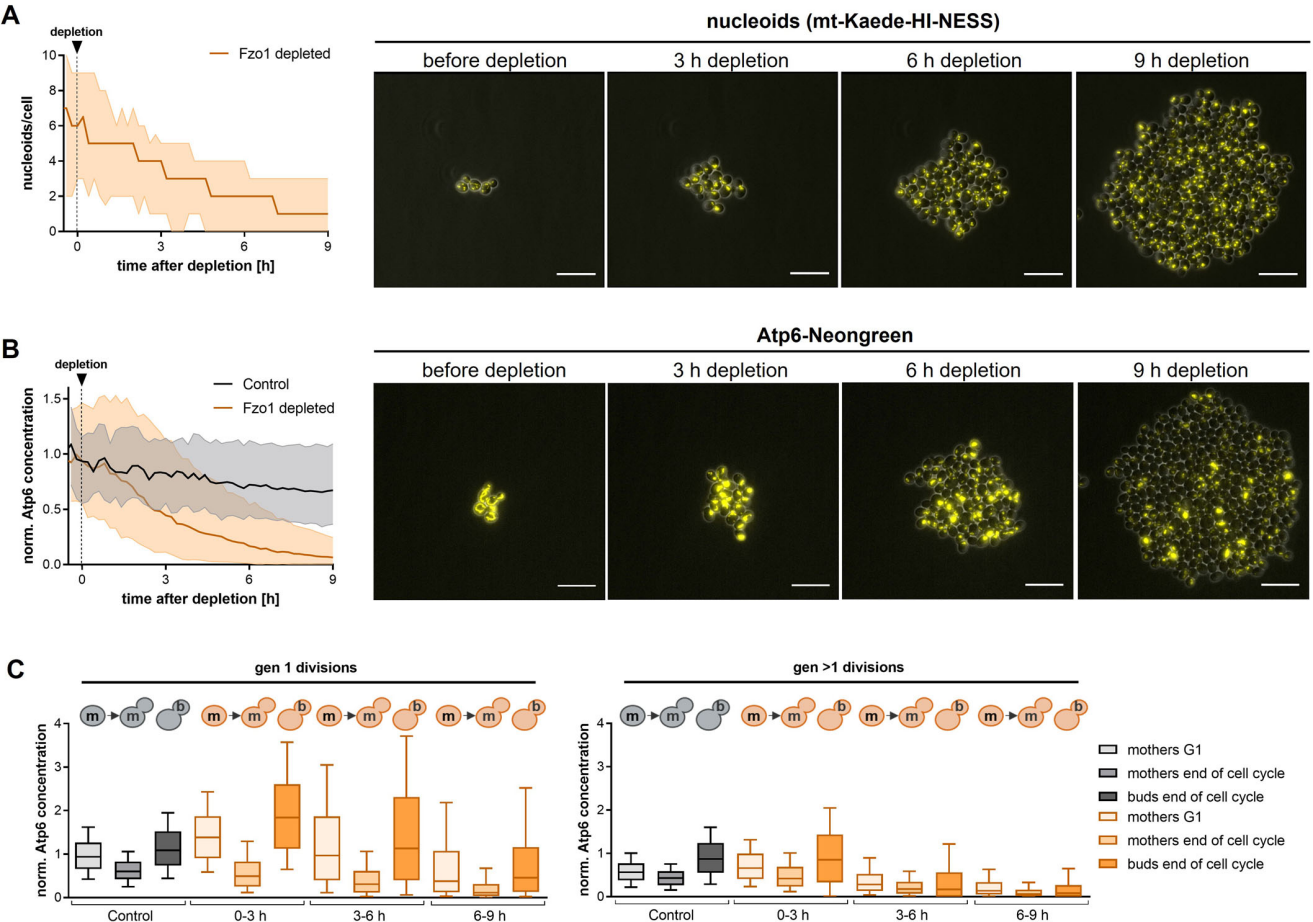
mtDNA-encoded proteins are segregated unequally during division. (**A**) Left: nucleoids per cell after Fzo1 depletion determined using the mt-Kaede-HI-NESS system, median with 25th and 75th percentiles. Number of cells at 9 h = 3451 Right: example images of mt-Kaede-HI-NESS. Scale bar = 20 µm. (**B**) Left: Atp6-mNeongreen concentration (total mitochondrial mNeongreen intensity per cell volume) after Fzo1 depletion. Median with 25th and 75th percentiles. Number of cells at 9 h: Control =  3818, Fzo1 depleted = 3686. Right: example images of Atp6-mNeongreen. Scale bar = 20 µm. (**C**) Atp6 concentration of mothers at the beginning of the cell cycle (G1), and mothers and buds at the end of the cell cycle. *n* (gen 1): Control = 1169, 0–3 h = 592, 3–6 h = 2384, 6–9 h = 844; *n* (gen >1): Control = 1486, 0–3 h = 865, 3–6 h = 3236, 6–9 h = 1092. Whiskers indicate 10th and 90th percentiles, line indicates median, boxes show 25th and 75th percentiles. Source data are available online for this figure.

In these strains, the median nucleoid count and the median Atp6 concentration rapidly dropped after Fzo1 depletion, reaching ~50% after 3 h and continuously dropping until 9 h. At 5 h, the 25th percentile of both nucleoids and Atp6 was already zero, indicating a rapid increase of petite cells in the population (Fig. 5A,B; Movies EV2,  EV3, and  EV4). Not just the median, but also the mean Atp6 concentration already starts to decrease around 3 h after depletion and reaches about 30% of initial levels at 9 h (Fig. EV5B), which is different from the mean mitochondrial concentration which stays constant within the first 9 h. Rapid mean Atp6 reduction is in agreement with the changes of the other mtDNA encoded proteins measured by Western blot and proteomics (Figs. 2B and EV2D). This rapid drop is not caused by mitophagy (Fig. EV5C,D), as it is also seen in Δ*atg32* cells, which are defective in mitophagy. We therefore hypothesized that it is a direct consequence of the unequal distribution of mitochondria and mtDNA-encoded proteins/nucleoids.

We thus examined if the Atp6 concentrations are also unequally distributed through division. We analyzed the Atp6 and nucleoid concentration of mothers and their buds at the end of the cell cycle. In first-generation divisions, the Atp6 concentration drops drastically in mother cells while their buds obtain as much as the mothers had at the beginning of the cell cycle (Figs. 5C, left and EV5E). In higher generation divisions, the Atp6 concentration of the mother also drops through cell division. While the median Atp6 concentration at the end of the cell cycle is also slightly higher in buds than in mothers, many buds do not receive any Atp6 (Figs. 5C, right and EV5E). This results in a lower ratio between buds and mothers at the end of division in higher generations compared to first generations (Fig. EV5F). Often, the Atp6 content present at the beginning of the cell cycle is distributed between mother and bud, resulting in a much lower concentration in both the mother and the bud at the end of division (Figs. 5C, right and EV5E). This implies that higher generation cells do not synthesize a sufficient amount of Atp6 during the cell cycle to supply both mothers and buds with an appropriate concentration of Atp6. This is likely also the case for the other mtDNA-encoded proteins.

### Compensatory synthesis of mtDNA-encoded protein fails after Fzo1 depletion

**Figure 6:**
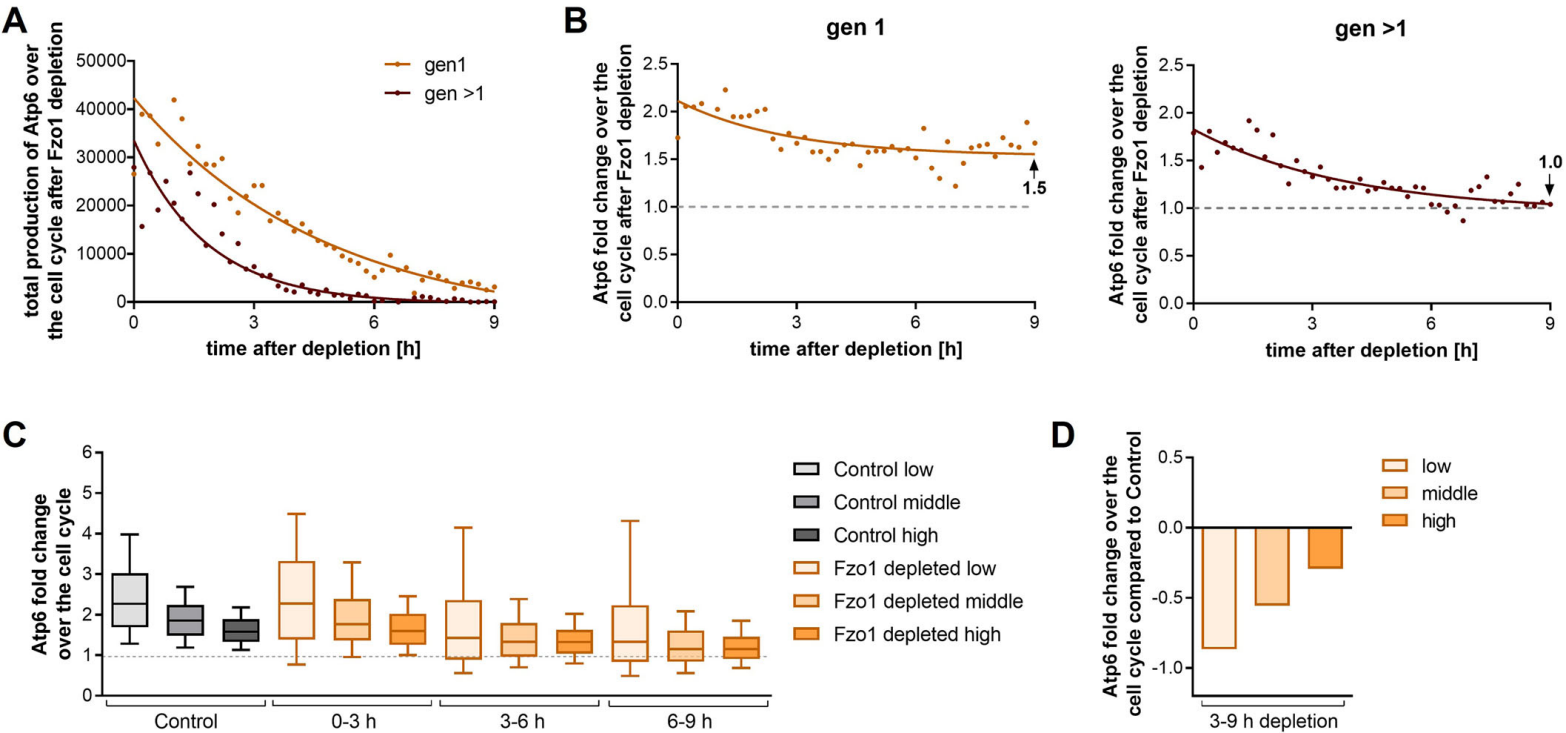
Synthesis of mtDNA-encoded proteins is reduced in Fzo1-depleted cells. (**A**) Total change of Atp6-mNeongreen (arbitrary fluorescence intensity units) through the cell cycle of first and higher generation divisions. 3820 divisions for gen 1 and 5193 divisions for gen >1 divisions were analyzed. The median of each timepoint is shown. (**B**) Fold change of Atp6-mNeongreen through the cell cycle of first and higher generation divisions based on data in (**A**). (**C**) Fold change of Atp6 per cell cycle of cells with low, middle, and high Atp6 content of cells shown in (**A**, **B**). Control (whole timeframe): each category: *n* = 831. Fzo1 depleted 0–3 h: *n* (low) = 276, *n* (middle) = 212, *n* (high) = 964. Fzo1 depleted 3–6 h: *n* (low) = 3006, *n* (middle) = 774, *n* (high) =  1672. Fzo1 depleted 6–9 h: *n* (low) = 1375, *n* (middle) = 172, *n* (high) = 296. Whiskers of box plots indicate 10th and 90th percentiles, line indicates median, boxes show 25th and 75th percentiles. (**D**) Difference of the median relative synthesis of Atp6 per cell cycle of Fzo1-depleted cells (3–9 h after Fzo1 depletion) compared to Control cells from data shown in (**C**). Source data are available online for this figure.

To determine if first or higher generation mothers exhibit differences in the amount of Atp6 they are able to synthesize, we calculated the change of Atp6 over a full cell division cycle. We subtracted the amount of Atp6 from the mother at the beginning of the cell cycle from the summed Atp6 amount of the mother and daughter at the beginning of the next G1. First-generation cells are able to synthesize Atp6 after Fzo1 depletion, but exhibit a continuous reduction in the amount of Atp6 produced within one cell cycle within the first 9 h after Fzo1 depletion. Higher-generation cells produce almost no Atp6 already 5 h after Fzo1 depletion (Fig. 6A; Movie EV2), indicating that they reach a deletion-like state faster than first-generation cells.

**Figure EV6:**
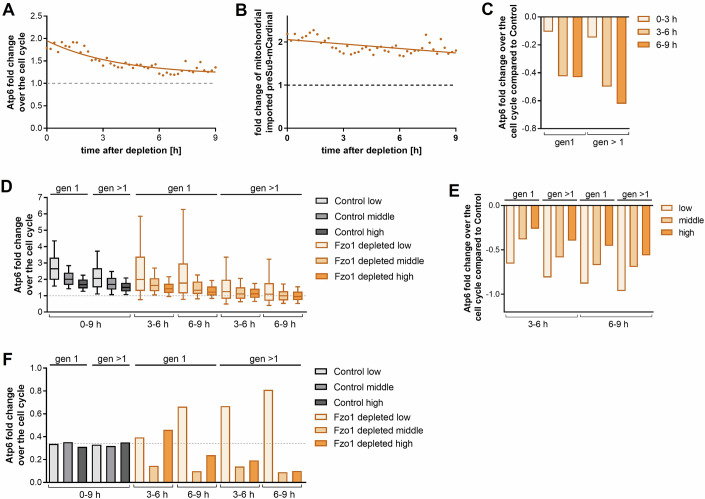
Synthesis of mtDNA-encoded proteins is most strongly reduced in higher generations. (**A**) Fold change of Atp6 per cell cycle of all generations after Fzo1 depletion, related to Fig. 6B. In all, 9013 cell divisions were analyzed. (**B**) Fold change of mitochondrial imported preSu9-mCardinal per cell cycle of all generations after Fzo1 depletion as in (**A**). (**C**) Difference of the median fold change of Atp6 over the cell cycle in Fzo1-depleted versus Control cells of gen 1 and gen >1 divisions, related to Fig. 6C. (**D**) Fold change of Atp6 over the cell cycle of first and higher generation divisions of mothers with low, middle, or high Atp6 content at the beginning of the cell cycle. At least 79 cell cycles were analyzed for each group. Whiskers indicate 10th and 90th percentiles, line indicates median, boxes show 25th and 75th percentiles. (**E**) Difference of the median Atp6 fold change of Fzo1-depleted cells compared to the respective Control group of cells shown in (**D**). (**F**) Fraction of the population of cells shown in (**D**).

We next determined the fold change of Atp6 over the cell cycle over time. If a cell produces exactly enough new mtDNA to maintain mtDNA concentrations over the cell cycle, then the expected fold-change value would be close to two (a little more in first-generation mothers since the total volume of the mother plus bud is more than twice the volume of a newborn cell). Before depletion, the fold change of Atp6 is close to this expected value but decreases gradually over time after Fzo1 depletion, reaching 1.3, averaged across generations at 9 h (Fig. EV6A), while the fold change of imported preSu9-mCardinal over the cell cycle stays almost constant (Fig. EV6B). However, first-generation cells at this time are still able to produce Atp6 with a fold change of 1.5, while higher generations are at 1.0, indicating that Atp6 is not synthesized and only diluted in these divisions (Fig. 6B). This again indicates that higher-generation cells likely become petite earlier than first-generation cells.

Since we observed a strongly unequal distribution of Atp6 within the population (Fig. 5A,B), we wanted to investigate whether the amount of Atp6 produced in a given cell is dependent on the initial amount of Atp6 at the beginning of its cell cycle. We therefore determined categories by dividing the Control population at the beginning of G1 into three equally large groups: low, middle, and high Atp6 amount. In Control cells, the cells with low Atp6 amount have a higher relative production of Atp6 over the cell cycle, while those with high Atp6 amount have a much lower relative synthesis (Figs. 6C and  EV6D). This indicates that in WT cells, a feedback mechanism exists, ensuring homeostasis through reduced or increased synthesis, similar to the mechanism observed in fission yeast (Jajoo et al, 2016). Fzo1-depleted cells lose this compensatory mechanism. Between 3 and 9 h after depletion, a reduction in the Atp6 fold change over the cell cycle compared to Control cells can be detected for all three categories (Fig. 6D). However, the difference compared to Control cells is most strongly pronounced in cells with low Atp6 content. While Control cells with low Atp6 content exhibit a ~2.5-fold change of Atp6 over the cell cycle to compensate for the low starting amount, Fzo1-depleted cells only exhibit a 1.4-fold change between 3 and 9 h after depletion. This suggests that the compensatory mechanism that ensures maintenance of mtDNA fails in Fzo1-depleted cells. Through the shift of the fraction of the population with extremely low and high Atp6 amount, the unequal distribution further enhances a reduction in the Atp6 fold change (Fig. EV6D–F). In conclusion, these results suggest that the combined effect of the unequal distribution and reduction in synthesis results in a reduction of Atp6 over time.

### Re-addition of Fzo1 enables partial recovery

**Figure 7:**
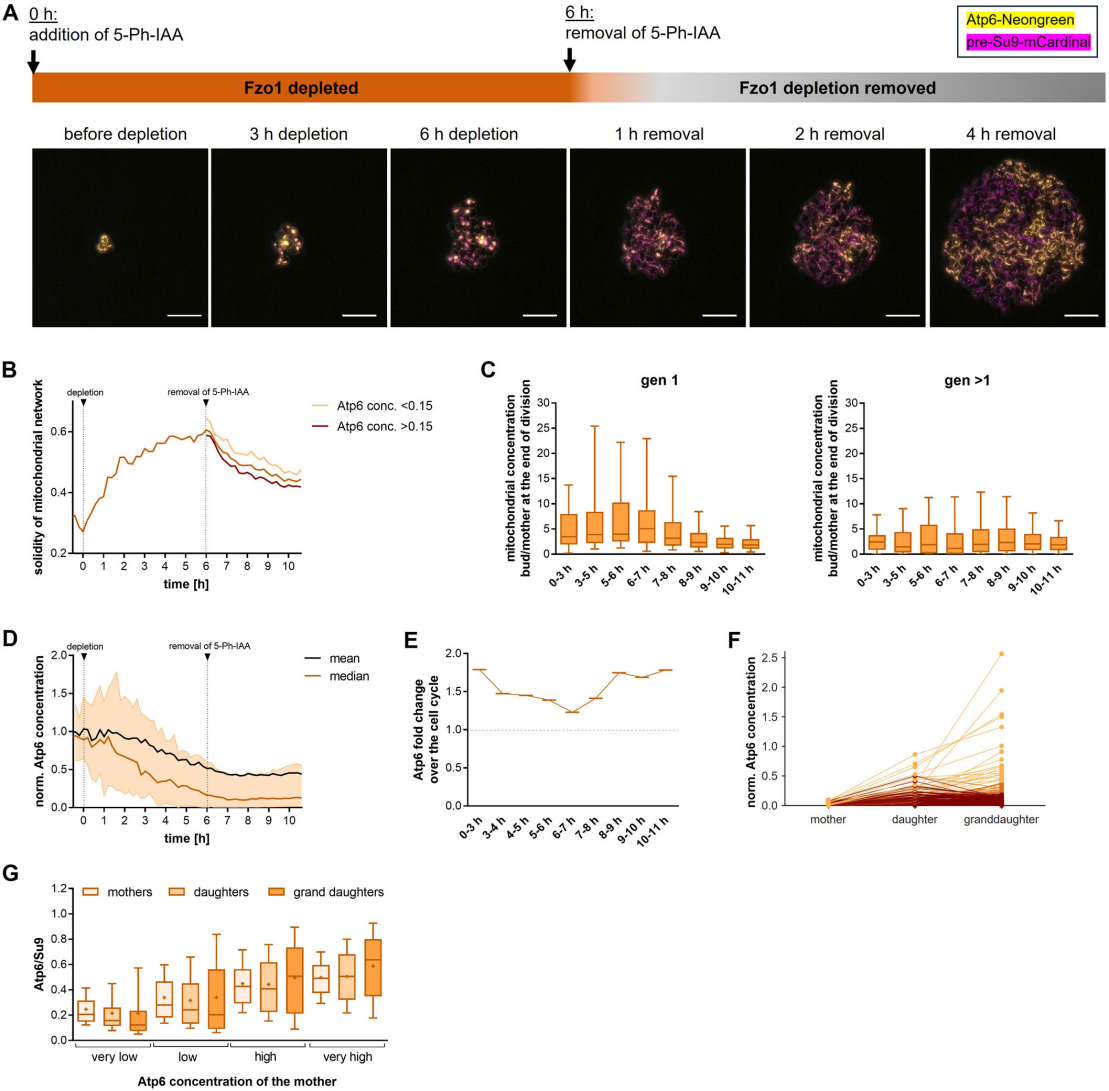
Re-addition of Fzo1 enables partial recovery. (**A**) Example pictures of Atp6-Neongreen (yellow) and pre-Su9-mCardinal (purple) after Fzo1 depletion and subsequent removal of 5-Ph-IAA. Scale bar = 20 µm. (**B**) Solidity of the mitochondrial network during depletion and after re-addition of Fzo1 (4179 cells from two biological replicates at 10.6 h). (**C**) Ratio of the mitochondrial concentration of the bud/mother at the end of division. Whiskers indicate 10th and 90th percentiles, line indicates median, boxes show 25th and 75th percentiles. Data from two biological replicates. Number of cells: gen1 (0–3 h) = 36, gen1 (3–5 h) = 65, gen1 (5–6 h) = 65, gen1 (6–7 h) = 104, gen1 (7–8 h) = 163, gen1 (8–9 h) = 301, gen1 (9–10 h) = 453, gen1 (10–11 h) = 405. Gen > 1 (0–3 h) =  42, gen > 1 (3–5 h) = 87, gen > 1 (5–6 h) = 91, gen > 1 (6–7 h) = 121, gen > 1 (7–8 h) = 165, gen > 1 (8–9 h) = 341, gen > 1 (9–10 h) = 506, gen > 1 (10–11 h) = 491. Brown–Forsythe tests comparing depletion at 5–6 h vs 10–11 h (corresponding to 4–5 h of removal of depletion) show no significant difference in spread for gen > 1 (*P* = 0.051; IQR 5–6 h = 5.63, IQR 10–11 h = 2.66), but a significant reduction in variability for generation 1 (*P* = 2.125 × 10^−6^, IQR 5–6 h = 7.44, IQR 10–11 h = 1.93). (**D**) Atp6 concentration of 4972 cells at 10.6 h cells from two biological replicates. Mean and median with 25th and 75th percentiles is shown. (**E**) Median Atp6 fold change over the cell cycle indicates recovery of mtDNA encoded protein synthesis. Data from two biological replicates. Number of cell cycles analyzed: 0–3 h: 78, 3–4 h: 59, 4–5 h: 95, 5–6 h: 158, 6–7 h: 252, 7–8 h: 354, 8–9 h: 638, 9–10 h: 963, 10–11 h: 881. (**F**) Atp6 concentration of mothers, daughters, and granddaughters of mothers with very low Atp6 concentrations. (**G**) Atp6/Su9 ratio for mother–daughter–granddaughter lineages starting with mothers with very low, low, high, and very high Atp6 concentrations at the beginning of their cell cycle (see Fig. EV7C for further information). Very low: *n* (mothers) = 106, *n* (daughters) = 139, *n* (granddaughters) = 155; low: *n* (mothers) =  164, *n* (daughters) = 162, *n* (granddaughters) = 161; high: *n* (mothers) = 163, *n* (daughters) = 163, *n* (granddaughters) = 162; very high: *n* (mothers) = 164, *n* (daughters) = 164, *n* (granddaughters) = 164; Whiskers indicate 10th and 90th percentiles. + indicate means, line indicates median, boxes show 25th and 75th percentiles. Source data are available online for this figure.

To further understand the causality between the different aspects of the phenotype, we analyzed their reversibility. We thus depleted Fzo1 for 6 h in our microfluidic setup, which results in a ~50% reduction of mtDNA and the proteins encoded by it (Figs. 7D and EV5A; Movie EV5). Following this depletion period, β-estradiol and 5-Ph-IAA were flushed out with growth medium without inducers, enabling a recovery of Fzo1 protein concentrations.

As described above (Figs. 1C and EV1A), mitochondrial morphology changes within the first 30 min after Fzo1 is depleted by 5-Ph-IAA addition. After removal of 5-Ph-IAA, mitochondrial morphology starts to become more tubular and wild-type-like within an hour, indicating that Fzo1 protein concentrations rapidly recover (Fig. 7A,B). Notably, almost all cells start regaining a more wild-type–like mitochondrial morphology (Fig. 7A), regardless of their expression of mtDNA-encoded Atp6-NeonGreen, even though cells with low Atp6 start off with a more “clumped” morphology at the time of Fzo1 re-addition. This demonstrates that reestablishment of mitochondrial morphology is largely independent of the presence of mtDNA, and that mitochondrial morphology changes are indeed the primary, but reversible phenotype of Fzo1 depletion.

**Figure EV7:**
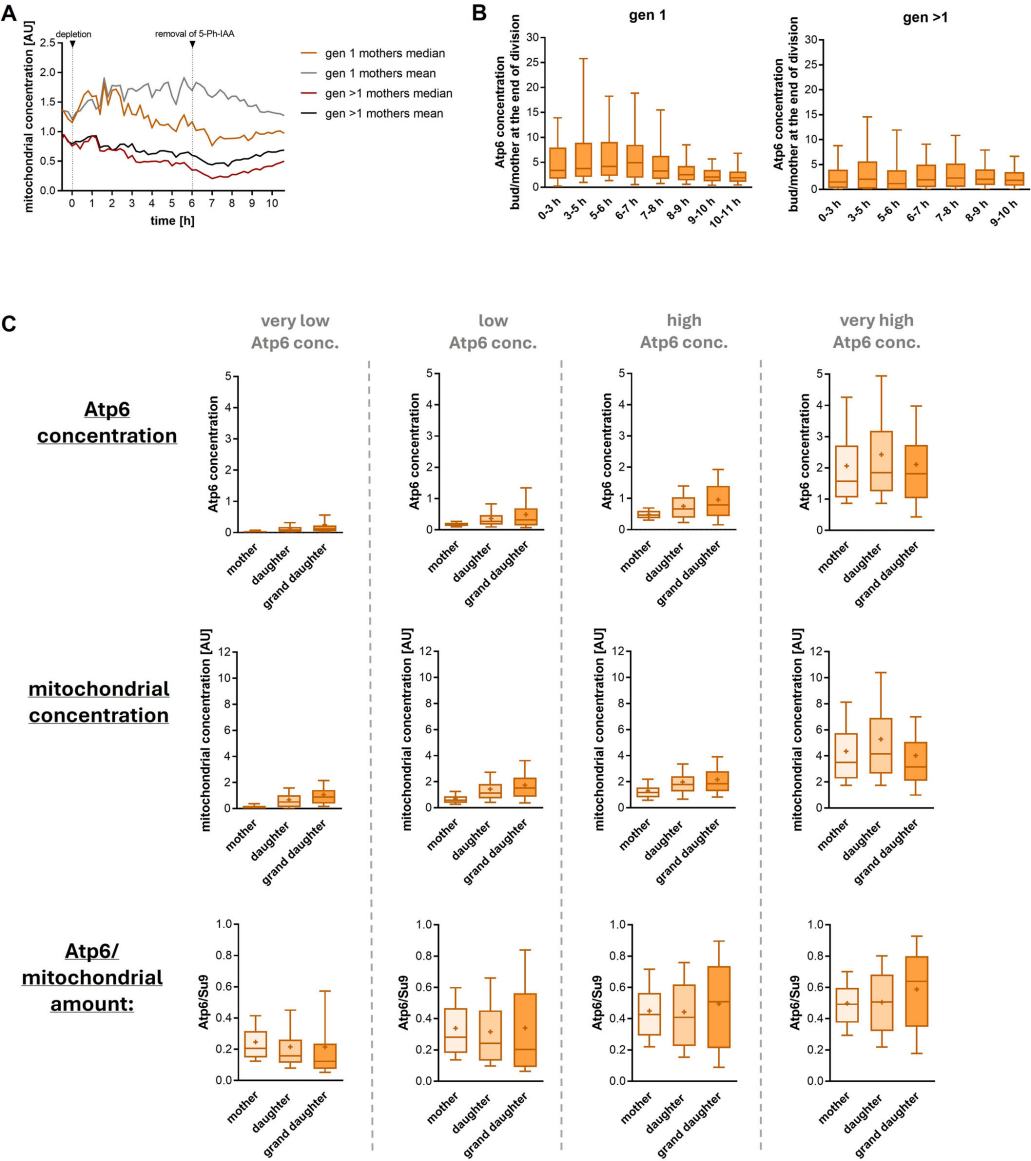
Cells with low amounts of mtDNA-encoded proteins are unable to recover after Fzo1 re-addition. (**A**) Mean and median mitochondrial concentration of first and higher generation mothers. 176 mothers at 10.6 h for gen 1 and 210 for gen >1. (**B**) Bud/mother Atp6 concentration ratios at the end of division as shown for the mitochondrial concentration in Fig. 7C. Whiskers indicate 10th and 90th percentiles, line indicates median, boxes show 25th and 75th percentiles. (**C**) Atp6 concentrations of mothers, daughters, and granddaughters after re-addition of Fzo1. Selection is based on daughters that were born at least 1 h after removal of 5-Ph-IAA. Grouping is based on the Atp6 concentration of mothers, which were divided into four equal groups. Each group contains 163 mother–daughter–granddaughter lineages. Whiskers indicate 10th and 90th percentiles, + indicates mean, line indicates median, boxes show 25th and 75th percentiles.

Since we had identified unequal mitochondrial partitioning during the cell cycle as a major factor contributing to mtDNA loss upon Fzo1 depletion, we next asked whether restoration of mitochondrial morphology is sufficient to reestablish a wild-type–like distribution of mitochondria between mother and bud. Indeed, after removal of 5-Ph-IAA, the bud/mother distribution gradually becomes more equal and wild-type-like. In first-generation divisions, the median and the spread of the bud-to-mother ratio are reduced. In higher-generation divisions, the spread is reduced, and the proportion of cells with a very low ratio is reduced (Fig. 7C). This results in an increase in the mean mitochondrial concentration in higher-generation cells (Fig. EV7A). Similar to mitochondrial mass, Atp6-Neongreen exhibited a more homogeneous distribution at the end of the cell cycle upon release from Fzo1 depletion compared to continuously Fzo1-depleted cells (Fig. EV7B).

We next analyzed whether this recovery in mitochondrial morphology and inheritance would lead to a recovery in Atp6 concentrations. After removal of the depletion (Fig. 7D), the mean and median Atp6 concentration remain stable, contrary to continued depletion (Figs. 5B and EV5A), where it further drops. In support of this, the median relative Atp6 synthesis, i.e., Atp6 fold change across one cell cycle, recovers to almost wild-type-like levels, showing that this phenotype is also reversible.

However, in the observed time frame, the Atp6 concentration does not increase on the population level. To understand this, we analyzed the Atp6 and mitochondrial concentrations of mothers, daughters, and granddaughters after removal of Fzo1 depletion. We selected mother–daughter-granddaughter pairings for which the daughters were born at least 1 h after removal of the depletion. We then grouped mothers into four equally large groups based on their Atp6 concentration at the beginning of their cell cycle (Fig. EV7C).

Except for the group starting off with very high Atp6 concentrations, the median Atp6 concentration increases from mothers to daughters to granddaughters. However, in the “very low” group, many cells do not recover at all or only very slowly (Fig. 7F). Thus, even after two generations, the median Atp6 concentration in this group is still much lower than in the wild-type. On the contrary, the mitochondrial concentration in these cells recovers almost completely in the same time frame. This discrepancy can be visualized by the Atp6 to preSu9-Cardinal ratio (Figs. 7G and EV7C, lower panel), which is a proxy for the relationship between mtDNA encoded and nuclear encoded mitochondrial biosynthesis.

In summary, the Fzo1 re-addition showed that the mitochondrial morphology, mitochondrial distribution, and mitochondrial biosynthesis are fully reversible. On the other hand, once cells reach a critically low concentration of Atp6 (mtDNA), this is not reversible.

### Mathematical modeling shows that unequal distribution of mitochondria in combination with the reduction of synthesis leads to a fast and complete establishment of the ∆*fzo1* phenotype

Our experiments showed that depletion of Fzo1 affects the inheritance of mitochondria to the bud at cell division. At the same time, it leads to decreased relative synthesis rate of mtDNA encoded Atp6 during the cell cycle, in particular for cells with low starting concentrations of Atp6. Since we consider Atp6 to be a good proxy for the amount of functional mtDNA, this decreased rate is likely due to a decreased synthesis of mtDNA. To dissect how these two effects contribute to loss of mtDNA, we built a simple model simulating mtDNA inheritance and synthesis over many cell cycles.

**Figure EV8:**
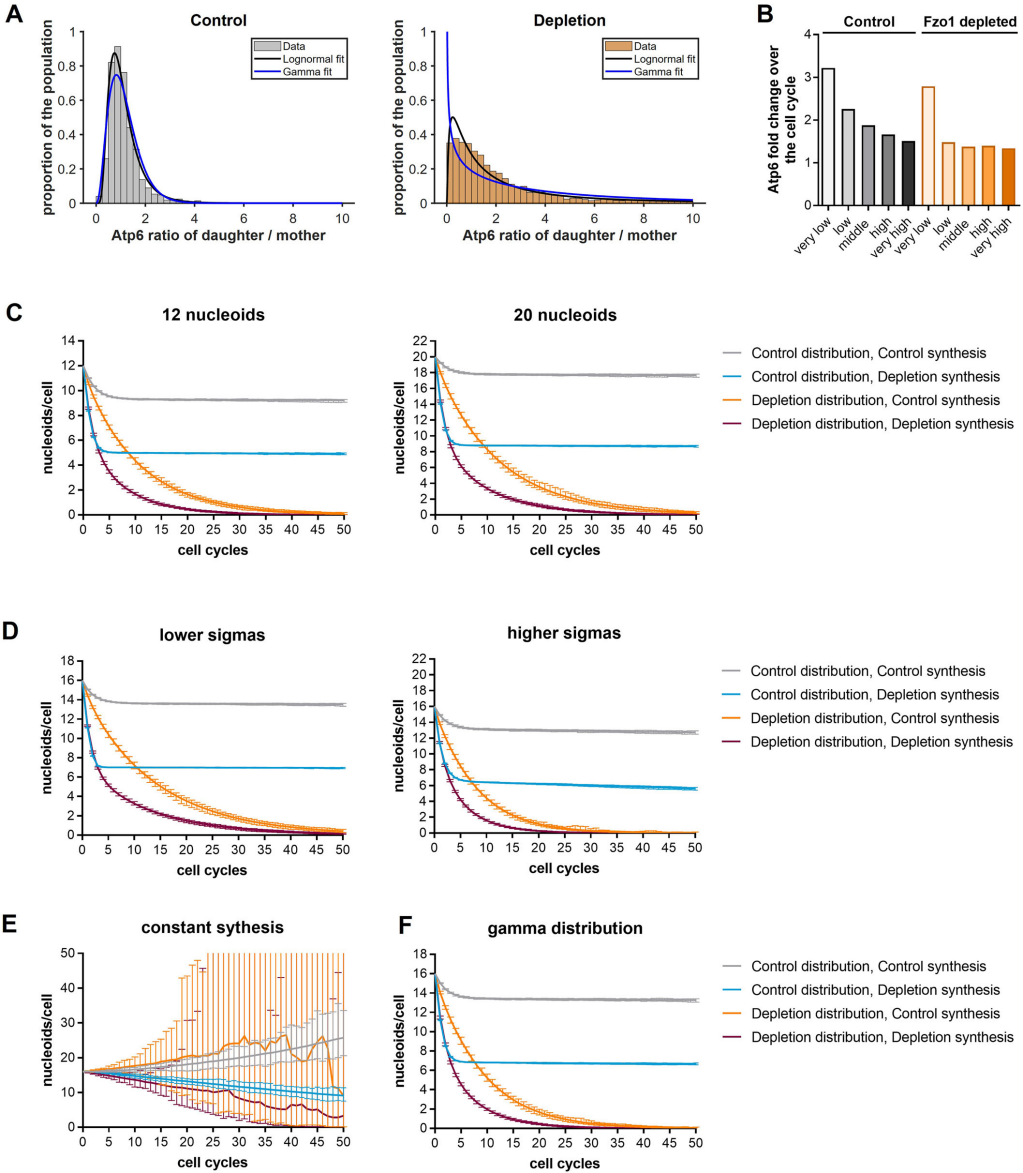
Sensitivity analysis of model parameters. (**A**) Histogram with lognormal (black line) and gamma (blue line) distribution fits of the ratio of Atp6 between daughters and their mothers immediately after division is completed. (**B**) Average fold change of Atp6 over the cell cycle of Control and Fzo1-depleted cells with different Atp6 content of the mother at the beginning of the cell cycle. mtDNA synthesis was determined based on the Atp6 content at the beginning of G1. (**C**–**E**) Nucleoid levels modeled with Atp6 daughter/mother ratios fitted with lognormal distributions. Mean and range of 100 simulations are shown. (**C**) Nucleoid levels modeled with lower (12 nucleoids) or higher (20 nucleoids) starting amounts compared to 16 nucleoids used for the simulation in Fig. 8. (**D**) Nucleoid levels modeled with lower and higher sigmas compared to the sigmas obtained from the measured data. (**E**) Nucleoid levels modeled with a constant synthesis rate instead of the category-dependent synthesis rates shown in (**B**). (**F**) Nucleoid levels modeled with a gamma distribution instead of the lognormal distribution. See “Methods” for further details.

For each simulation, we initiated a population of 10,000 cells, each starting with 16 nucleoids, the structures that contain mtDNA (Roussou et al, 2024), and each was about to divide. We then assumed that each cell would distribute all its nucleoids between bud and mother cell with a ratio randomly drawn from a log-normal distribution. The parameters for the log-normal distribution were experimentally determined for both Control cells (during the entire time period) and Fzo1-depleted cells (3–9 h after onset of depletion) (Fig. EV8A). After cell division, the number of total cells was randomly reduced by 50% to again obtain 10,000. Next, we simulated mtDNA synthesis during the cell cycle, where the amount of synthesis depends on the initial nucleoid number, which was sorted into five groups from very low to very high (Fig. EV8B, see “Methods” and Figure Caption for further details). The values for this relative synthesis rate were determined from experiments and were different for Control and Fzo1-depleted cells (Fig. EV8B). Continuing with the simulation of the next cell division, this procedure was then repeated for a total of 50 cell cycles.

**Figure 8:**
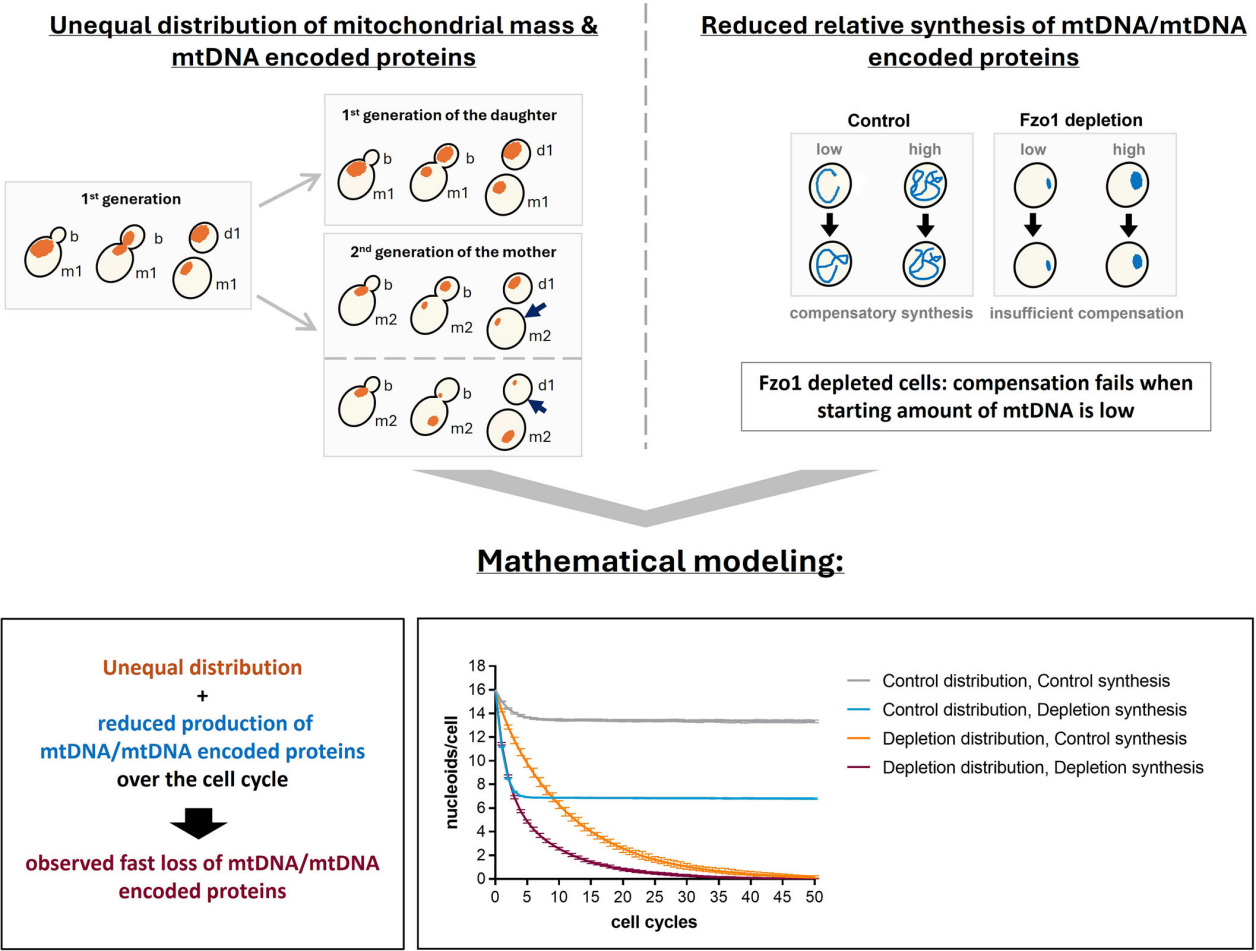
The combination of unequal segregation and reduced synthesis is sufficient to explain the ∆fzo1 petite phenotype. Schematic depiction of observed phenotypes. Left: unequal distribution, right: reduced synthesis of mtDNA/mtDNA encoded proteins. Bottom: mtDNA level modeled with Control or Fzo1 distribution and synthesis of mtDNA encoded Atp6. See Fig EV8 and methods for model assumptions and parameters. Source data are available online for this figure.

We show that the segregation of mtDNA between bud and mother as approximated by Atp6 measurements, together with the five categories of mtDNA/Atp6 fold change over the cell cycle are sufficient to recapitulate mtDNA homeostasis in WT-like cells, as the simulated levels for Control cells are stable over time (Fig. 8). Contrary to this, Fzo1 depletion-like segregation of nucleoids between bud and mother combined with Control cell Atp6 synthesis, results in a continuous but slow decrease of nucleoid number over time. On the other hand, WT-like segregation combined with Fzo1 depletion-like synthesis leads to a rapid decrease in nucleoid numbers within the first divisions, followed by a very slow decrease during the following divisions. Only the combined effect of both, the unequal distribution and reduced synthesis, leads to loss of nucleoids with dynamics that match those we observed experimentally.

Taken together, our mathematical model shows that both factors, the unequal distribution of mtDNA and a reduction in relative synthesis, are required and also sufficient to explain the development of the Fzo1 phenotype.

## Discussion

Here, we unravel how mtDNA is lost in cells lacking an active mitochondrial fusion machinery. We used the AID-system to rapidly deplete the mitochondrial outer membrane mitofusin Fzo1 in combination with microfluidics-based, quantitative single-cell time-lapse imaging of tens of thousands of cells across their cell cycles. With these tools, we could resolve the timing of the individual defects that lead to loss of mtDNA: altered mitochondrial morphology, reduction of the mitochondrial membrane potential (MMP), and reduced synthesis of mtDNA and mtDNA-encoded proteins.

In line with a previous study (Hermann et al, 1998), we show that fragmentation of the mitochondrial network is the primary phenotype of Fzo1 depletion and occurs in less than one hour. Simultaneously with mitochondrial fragmentation, we observed an initial drop in mitochondrial membrane potential (MMP), which decreased further until 21 h of Fzo1 depletion. Approximately at the time MMP reaches an ∆*fzo1* deletion-like state, also deletion-like growth is reached.

Surprisingly, most of the mtDNA (Fig. 2A) and mitochondrially encoded proteins (Fig. 2B) were lost within the first 9 h of Fzo1 depletion. Directly following the loss of mtDNA and the proteins encoded by it, we observed a reduction of nuclear-encoded respiratory chain proteins. This results in a strong decrease in ATP synthase, which is required for normal mitochondrial ultrastructure. Consequently, we detected a stepwise change in mitochondrial ultrastructure from mostly cristae to a mixture of cristae and aberrant mitochondria to an ultrastructure that resembles petite cells. As observed for other parameters, mitochondrial ultrastructure resembles that of ∆*fzo1* cells after 21 h of depletion.

After 21 h, the Fzo1 depletion shows changes comparable to ∆*fzo1* cells in the proteome, such as downregulation of respiratory chain proteins and upregulation of glycolytic enzymes (Appendix Fig. S1). These larger-scale changes in the proteome only start approximately 9 h after Fzo1 depletion, when most of the mtDNA has already been lost (Appendix Fig. S1). These proteome changes are most likely a generic response to the metabolic deficiencies caused by the absence of mtDNA, rather than specifically to Fzo1 depletion, as petite cells obtained by ethidium bromide treatment showed comparable proteome changes (Vowinckel et al, 2021). Supporting this, it was postulated that mitochondria-to-nucleus communication is mostly mediated by the cytosolic signaling machinery as a response to metabolic changes (Knorre et al, 2016). In line with an absence of signaling to the nucleus, we detected no changes in the promoter activity of nuclear genes encoding mitochondrial proteins involved in respiration within the first 9 h of depletion, even though the protein levels strongly declined.

While the growth limitations and metabolic stress reactions of the final phenotype of petites are similar regardless of how petites emerged, the mechanisms leading to the loss of mtDNA are likely to differ. The loss of mtDNA and the proteins encoded by it may be expected to be caused by mitophagy of low-functioning mitochondria, but we could exclude a major role of mitophagy (Fig. EV5C,D). Also, mtDNA integrity does not seem to be affected since different loci on the mtDNA decreased with a similar rate, as also seen in mouse embryonic fibroblasts with a mitofusin deletion (Silva Ramos et al, 2019).

By analyzing the dynamics of mitochondria, nucleoids, and mtDNA-encoded proteins on a single cell level using live-cell time-lapse imaging, we were able to identify the underlying cause of mtDNA loss: the combination of unequal inheritance of mitochondria and decreased synthesis of mtDNA. Despite the mitochondrial inheritance delay of Fzo1-depleted cells as described for ∆*fzo1* cells (Böckler et al, 2017), at the end of the cell cycle buds obtain a high concentration (total mitochondrial signal intensity per cell volume) of mitochondria, whereas mothers often only retain a low concentration compared to their buds (Fig. 4F). While the overall mitochondrial mass within the population is maintained within the first 9 h of depletion, this unequal distribution results in a progressive decline of mitochondrial health within the population. This is in line with a recent preprint (preprint: Ray et al, 2025) showing that cells that inherit very low concentrations of mitochondria are more likely to become petite. This unequal distribution also impacts mtDNA maintenance. Control cells maintain stable mean mtDNA levels across the population, even if starting levels in individual cells fluctuate (Fig. 6C), suggesting a compensatory mechanism. In contrast, in Fzo1-depleted cells, this compensatory mechanism fails. Cells with low amounts of mitochondrial encoded Atp6 show a strong reduction in synthesis rates compared to wild-type-like cells with similar starting amounts, and can thus not recover if they do not inherit a sufficient amount of mtDNA and the proteins encoded by it. This combination of unbalanced inheritance and reduced synthesis is sufficient to explain the observed fast and complete loss of mtDNA in Fzo1-depleted cells in a simple mathematical model (Fig. 8). While the loss of mtDNA becomes irreversible, mitochondrial morphology, content, and inheritance do recover when Fzo1 is re-expressed (Figs. 7 and EV7), indicating that these are largely independent of mtDNA.

Why are Fzo1-depleted cells unable to compensate for the low levels of mitochondrial-encoded proteins through increased synthesis, unlike wildtype cells? We hypothesize that several factors contribute to this phenomenon: In WT cells, mtDNA-containing nucleoids are regularly spaced, but in Fzo1-depleted cells, they may not be distributed across all mitochondrial fragments. Clustering of nucleoids was shown to impact mtDNA replication in mouse embryonic fibroblasts lacking mitofusins through an imbalance in replisome components (Silva Ramos et al, 2019).

Mitochondrial fragmentation may not only lead to unequally distributed nucleoids, but also unequally distributed import complexes within individual mitochondrial fragments. Khan and colleagues (Khan et al, 2024) postulate that this unequal distribution of import complexes might contribute to the reduction of MMP in Fzo1 deletion cells. Supporting this hypothesis, we detected an immediate reduction of MMP concurrently with fragmentation of the mitochondrial network.

The MMP serves as the driving force for the import of nuclear-encoded mitochondrial proteins into mitochondria. A reduction in MMP, therefore, leads to impaired mitochondrial import. Deficiency in the import of mitochondrial proteins eventually results in their degradation in the cytosol (Liu et al, 2021). Since nearly all mitochondrial proteins are nuclear-encoded, this disruption compromises mitochondrial functionality. Coordinated and efficient import is particularly important for components of the respiratory chain, as these proteins are highly hydrophobic and require the simultaneous provision of mtDNA-encoded subunits for the assembly of functional complexes. The stability of mtDNA-encoded proteins is affected by nuclear-encoded ones (Contamine and Picard, 2000). Therefore, impaired mitochondrial import of nuclear-encoded proteins through MMP reduction likely affects the stability of mtDNA-encoded proteins in Fzo1-depleted mitochondria. Complete loss of mtDNA leads to a significant reduction in the levels of nuclear-encoded respiratory chain proteins (Dagsgaard et al, 2001; Vowinckel et al, 2021). This highlights the complex interdependence between the stability and biogenesis of mtDNA and nuclear-encoded components, which likely drives a progressive decline in mitochondrial function if not carefully balanced. As a result, both mitochondrial membrane potential (MMP) and mitochondrial content are diminished, ultimately contributing to a decline in cellular health and a slowing of cell cycle progression in line with recent observations by others in budding yeast, fission yeast, and HeLa cells (Chacko et al, 2025; Gorospe et al, 2023; Jajoo et al, 2016; Johnston et al, 2012; Vowinckel et al, 2021).

Taken together, we show here that mtDNA loss in the absence of mitochondrial fusion is a consequence of complex, intertwined mitochondrial defects, with stochastic inheritance playing a key role. The use of single-cell time-lapse microscopy analysis across multiple generations proved essential to uncover the causality behind the emergence of these phenotypes. This approach allowed us to show how mitochondria with altered morphology and functionality are segregated during asymmetric division. Asymmetric division in metazoans allows distinct fates for their progeny, which is also reflected in mitochondrial inheritance (Hinge et al, 2020). In human stem-like cells, for example, younger mitochondria are preferentially retained in the stem cell to preserve its properties (Hinge et al, 2020; Katajisto et al, 2015). Similarly, undifferentiated lymphocytes preferentially inherit the youngest mitochondria, while senescent and differentiated lymphocytes receive the oldest mitochondria (Adams et al, 2016). By revealing how mitochondrial defects affect inheritance, our study enhances the understanding of mitochondrial segregation during asymmetric division with relevance to all types of asymmetric divisions where mitochondrial morphology or function is altered.

## Methods

**Table:** Reagents and tools table

Reagent/resource	Reference or source	Identifier or catalog number
**Experimental models**		
*S. cerevisiae W303*		See Appendix Table S1
**Recombinant DNA**		
Plasmids	This study and (Azizoğlu et al, 2023)	See Appendix Table S3
**Antibodies**		
Anti-FLAG M2 antibody	Sigma	Product #:F1804
Anti-mouse IgG (H + L), HRP conjugate	Promega	W4021
Polyclonal rabbit anti-Cox2 antibody	Rapaport Lab	Singhal et al, 2017
Anti-rabbit IgG (H + L), HRP conjugate	Promega	W4011
**Oligonucleotides and other sequence-based reagents**		
PCR primers	This study	See Appendix Tables S6 and S7
**Chemicals, enzymes, and other reagents**		
FastStart Universal SYBR Green Master	Roche	Cat #4913850001
YeaStar RNA Kit	Zymo Research	Cat #R1002
high-capacity cDNA reverse-transcription kit with RNase inhibitors	Thermo Fisher Scientific	Cat #4374966
**Software**		
Image Studio Lite Ver 5.2.5	Li-COR	https://licor.app.box.com/s/4hrk823vov7vittqjg3onj51tb0wbo6w
QuantStudio 7 Flex Real-Time PCR system	Applied Biosystems, Thermo Fisher Scientific	
Cell-ACDC	Padovani et al, 2022	
Spotmax	Padovani et al, 2024	
Perseus version 2.0.5.0	Tyanova et al, 2016	
Spectronaut version 18	Bruderer et al, 2017	
Matlab R2017a	MathWorks	
**Other**		
Ti2-E inverted epifluorescence microscope	Nikon	
LSM 800 confocal microscope	Zeiss	
CellASIC ONIX2 system	Merck	
Optical filters for epifluorescence microscopy	AHF	See Appendix Table S4
Orbitrap Exploris 480 online coupled to an EASY nLC-1200 system	Thermo Fisher Scientific	
Licor Odyssey FC	Li-COR	

### Strain construction

All strains were haploid W303 derivatives, see genotype in Appendix Table S1. The N-terminal FLAG-AID-tag on Fzo1 was integrated using CRISPR Cas9 as described previously (Azizoğlu et al, 2023). Construction of the tetracycline-inducible TIR plasmid is based on (Azizoğlu et al, 2023), and estradiol-inducible TIR plasmid construction is based on (Ottoz et al, 2014). Strains were otherwise constructed using standard PCR-based homologous recombination or integration of linearized plasmids. See Appendix Table S2 for details of plasmids used for integration. All strains constructed in this study are available from the corresponding author upon request.

### Cultivation conditions

Cultures were grown to log phase in SC-Glucose (1.7 g/l yeast nitrogen base without amino acids (US Biological), 5 g/l ammonium sulfate, 50 mM potassium phthalate, pH adjusted to 5 with KOH, synthetic complete with all amino acids, see Appendix Table S3 for concentrations). TIR expression was induced by the addition of anhydrotetracycline (aTC, 0.2 mg/mL Stock, dissolved in 100% ethanol) to a final concentration of 50 ng/mL for 2 h. Cultures were diluted to OD_600_ = 0.1, and depletion was induced by the addition of 5 µL 5-Ph-IAA (2-(5-Phenyl-1H-indol-3-yl)acetic acid, 100 mM Stock, dissolved in DMSO) to a final concentration of 2 µM. Cultures were diluted every 3 h, and OD_600_ was determined every hour. Samples were withdrawn for DNA isolation and protein extraction (see below) before dilution, frozen in liquid nitrogen and stored at −70 °C. Experiments in which mitochondrial translation was inhibited were performed in a ∆*pdr5* background to ensure efficient translation inhibition (Roussou et al, 2024). Cultures were grown to log phase and treated with 2 mg/mL Chloramphenicol (CAP) and either untreated or Fzo1-depleted (see above).

### Microfluidic cultivation

In preparation for live-cell imaging, cells were grown overnight in SC-Glucose, diluted 1:50 the next morning, and grown for 6 h. For the late time frame after Fzo1 depletion (8–18 h and 9–24 h), cells were grown to log phase overnight, treated with 50 ng/mL aTC or 200 nM β-estradiol (dissolved at 1 mM in 100% ethanol) for 2 h to induce TIR expression. Cells were then diluted to OD_600_ = 0.01, aTC and 5-Ph-IAA (2 µM final) were added, and cells were treated for the respective time. Cells were sonicated at low power for 3 s and loaded onto a commercial microfluidics system (Y04C-02 plates, CellASIC ONIX2 system, Merck). SC-Glucose medium, with either no addition for untreated cells or supplemented with 100 ng/mL aTC or 800 nM β-estradiol (due to the hydrophobicity of the PDMS, higher concentrations are required) and 2 µM 5-Ph-IAA for depletion, was supplied with a pressure of 3 psi. Cells were grown inside the microfluidic chamber for the duration of 1.5 h before imaging started. The temperature was kept constant at 30 °C using an incubator chamber surrounding the imaging system (Okolab Cage Incubator, Okolab USA INC, San Bruno, CA).

### Epifluorescence microscopy

Epifluorescence microscopy was used for most imaging experiments. These were performed on a Nikon Ti2 inverted epifluorescence microscope (Nikon Instruments, Japan) with a Lumencor SPECTRA X light engine (Lumencor, Beaverton, USA), a Photometrics Prime 95 (Teledyne Photometrics, USA) backilluminated sCMOS camera. The system was programmed and controlled by the Nikon software NIS Elements. Focus was maintained using the Nikon “Perfect Focus System.” The time-lapse images were taken using a Nikon PlanApo oil-immersion ×100 objective (NA = 1.45) with a frequency of 8 min for only preSu9-mCardinal experiments and every 12 min for experiments in which two fluorophores were imaged. Nine z-slices were acquired with a step size of 0.45 µm. See Appendix Table S4 for optical filters and Appendix Table S5 for exposure settings. For all fluorophores and tagged proteins, we checked for the absence of phototoxicity (Cuny et al, 2022) by comparing growth rates in the microscopy setup to growth rates in shake flasks (see Fig. EV1F,G). We further confirmed that mitochondrial morphology was not affected by imaging, since Control cells maintained a constant average morphology over the imaging period of 9 h (Fig. EV1A).

### Confocal microscopy

Confocal live-cell imaging was used for Figs. 1B,C, EV3A,B, and EV4F and carried out on a Zeiss LSM 800 microscope equipped with an Axiocam 506 camera, and using a 63×/1.4 NA oil DIC objective. The Incubator XLmulti S1, Pecon, was used to maintain a temperature of 30 °C. mCardinal was imaged with an excitation wavelength of 561 nm and detected with an emission between 610 and 700 nm. Bright-field images were taken using the transmitted light detector (T-PMT). In total, 15 z-slices with a step size of 0.5 µm were taken every 30 s or every 3 min. For the control experiment in Fig. EV3A,B 35 z-slices with a step size of 0.23 were taken at the respective time points.

### Image analysis and data processing

Images were recorded at 12-bit gray scale and then converted to tiffs in Cell-ACDC (Padovani et al, 2022). Cell segmentation was performed in Cell-ACDC using YeaZ (Dietler et al, 2020), minimum area: 10 pixels, minimum solidity 0.5, maximal elongation 3.0). Each time frame was then visually inspected, and any segmentation and tracking errors were corrected in Cell-ACDC. Cell volumes were determined automatically in Cell-ACDC as described in (Padovani et al, 2022). The median background fluorescence (cell-free area) for each image was determined and subtracted from the signal at each time point. Cell cycle states and the mother-bud connections were annotated manually in Cell-ACDC, which allows us to extract mother–daughter relationships and generation numbers. Mitochondria were segmented in 3D using SpotMAX (preprint: Padovani et al, 2024) based on mitochondrially localized preSu9-mCardinal. For pre-processing in SpotMAX we used a Gaussian filter with sigma 0.7 followed by a Sato filter with two sigmas 1.0 and 1.5 to enhance network-like structures (Sato et al, 1998). The pre-processed images are then segmented by SpotMAX using Otsu thresholding (Otsu, 1979). Mitochondrial amounts of imported preSu9-mCardinal and Atp6-Neongreen were calculated by Cell-ACDC as the amount of mCardinal or Neongreen within the 3-dimensional mitochondrial segmentation mask. The amounts are calculated as follows: amount = (mean_obj − median_background)*area_obj. Cytosolic protein amounts were determined as follows: A 3D mask was generated from the cell segmentation by stacking the middle slice in a cylinder. From this, the mitochondrial segmentation was subtracted to obtain an approximation of the cytosol. Mitochondrial membrane potential (MMP) using the MitoLoc reporter modified from (Vowinckel et al, 2015) was calculated as follows:

$${{\rm{MMP}}}= 	\left(\frac{{{\rm{mitochondrial}}}\; {{\rm{imported}}}\; {{\rm{preCox}}}4-{{\rm{Neongreen}}}}{{{\rm{mitochondrial}}}\; {{\rm{imported}}}\; {{\rm{preSu}}}9-{{\rm{mCardinal}}}}\right)\, / \\ 	\, \left(\frac{{{\rm{cytosolic}}}\; {{\rm{preCox}}}4-{{\rm{Neongreen}}}}{{{\rm{cytosolic}}}\; {{\rm{Su}}}9-{{\rm{mCardinal}}}}\right)$$

We quantified nucleoids using the mt-Kaede-HI-NESS system as described recently (preprint: Deng et al, 2025). To count the number of spots, we used SpotMAX with the Li algorithm (Li and Lee, 1993) for segmentation and local peak detection. The spheroid radii were determined through visual inspection, and they are 0.453 μm in the *xy* directions (twice the Abbe’s diffraction limit) and 1 μm in the z-direction. The .ini files containing all parameters used in SpotMAX have been uploaded to GitHub.

### SDS-PAGE and Western blot

Pellets were frozen in liquid nitrogen and stored at −70 °C. Lysates were prepared with a FastPrep® shaking three times 40 s at 6 m/s with a 1-minute break in between each cycle using lysis buffer (50 mM Tris-HCl pH 8.0, 150 mM NaCl, 5 mM EDTA, 1% Tergitol) supplemented with 2 × EDTA-free protease inhibitor (GoldBio). In total, 30–50 µg total protein were loaded on 8, 10, or 14% SDS polyacrylamide (29:1 Bio-Rad) gels. SDS gels were blotted using a commercial wet transfer system (Bio-Rad). For the detection of 3xFLAG anti-FLAG M2 antibody (Sigma, product number: F1804) (1:5000 in milk powder) and anti-mouse IgG (H + L), HRP conjugate (Promega, W4021) was used. For the detection of Cox2, a custom polyclonal rabbit anti-Cox2 antibody (Singhal et al, 2017) (1:2000 in milk powder) and anti-rabbit IgG (H + L), HRP conjugate (Promega, W4011) were used. Luminescence was imaged on a Licor Odyssey FC. Bands were quantified using the image analysis software Image Studio Lite Ver 5.2.5 (Li-COR https://licor.app.box.com/s/4hrk823vov7vittqjg3onj51tb0wbo6w). See Appendix Fig. S3 for images of uncut blots.

### DNA quantitative PCR (qPCR)

To isolate complete genomic DNA (gDNA), phenol-chloroform-isoamyl alcohol (PCI) extraction was performed. For this, the pellets were first dissolved in 200 μL DNA extraction buffer (pH 8.0) and transferred into a 1.5 mL safe-lock tube containing 200 μL PCI and ~300 µg of glass beads. The cells were mechanically disrupted by vortexing for 20–30 s, and incubated for 5 min. Then, 200 µL TE buffer was added, samples were inverted a few times and centrifuged at 13,300 rpm for 5 min. In all, 250 µL aqueous phase was then transferred to a new reaction tube containing 1 mL of 100% EtOH to precipitate the gDNA. The mixture was then centrifuged again for 5 min at 13,300 rpm and the supernatant was discarded. The gDNA pellet was washed with 70% EtOH, centrifuged at 13,300 rpm for 5 min, and the supernatant was again discarded. The pellet was dried at 40 °C for 20 min and dissolved in 50 μL of nuclease-free water. DNA for quantitative PCR was subjected to RNase A digestion to remove RNA residues. Therefore, the solubilized pellet was treated with 1 mg/mL RNase A (ribonuclease A, 10 mg/mL, 50 U/mg, DNase-free, Roche) and incubated at 37 °C for 30 min. To inactivate RNase A, DNA extraction buffer and PCI were added, and the extraction steps were repeated. One microliter of the sample was loaded on an NP80 spectrophotometer, and the concentration was determined by absorbance at 260 nm. For qPCR, the samples were diluted to 0.25 ng/µL, and 1 ng of DNA was used in 10 µL total reaction volume.

qPCR was performed on a QuantStudio 7 Flex Real-Time PCR system (Applied Biosystems, Thermo Fisher Scientific, Waltham, USA) in 384 multiwell plates. For amplification, a DNA-binding fluorescent dye (FastStart Universal SYBR Green Master (Rox), Roche) and specific primers for the nuclear DNA gene *ACT1* and the mtDNA genes *COX2*, *COX3*, and *COB* (Appendix Table S6) were used. For the calculation of the mtDNA fold change, the average Ct of three technical replicates was used. Technical replicates were excluded if the standard deviation was higher than 0.5. The average of the nuclear control gene *ACT1* was then subtracted for each gene from the average Ct for each sample. Each gene was normalized on the 0 h Control sample. The fold change was then calculated by 2^-(ΔΔCT)^. The average change of mtDNA encoded genes is presented as the average of the three mitochondrial encoded genes *COX1*, *COX2*, and *COB*.

### RT-qPCR

The pellet of 10 mL culture was washed once with nuclease-free water and centrifuged at 5000 × *g* for 1 min. RNA was extracted using the YeaStar RNA Kit (Zymo Research) according to the manufacturer’s instructions. DNase digestion step was performed to remove DNA contamination. 1 µg of each sample was then reverse transcribed into cDNA using the high-capacity cDNA reverse-transcription kit with RNase inhibitors (Thermo Fisher Scientific). See Appendix Table S7 for primers used.

### DAPI staining

For DAPI staining, strain KK144 was grown to log phase overnight. Then, cells were Fzo1 depleted for 6 h and stained with 5 µg/mL DAPI (1 mg / mL stock in H_2_O) for 30 min before microscopy.

### TMRM staining

For TMRM staining, strain KK150 was grown to log phase overnight. Then, cells were Fzo1-depleted for 8 h. 1 mL culture was centrifuged at 13,000 rpm for 30 s. Cells were washed in 1 mL 10 mM HEPES pH 7.4 + 5% glucose. Staining was performed for 20 min at 30 °C with 20 nM TMRM in 10 mM pH 7.4 + 5% glucose. Before microscopy, cells were washed twice with 10 mM HEPES + 5% glucose and then resuspended in 100 µL.

### Electron microscopy

For electron microscopy, cells were grown as described in the cultivation conditions. For 21 h samples, cells were diluted to a lower OD_600_ after the 9 h timepoint and grown until 21 h to log phase. Microwave-assisted preparation and analysis of electron microscopy samples was performed as published recently (Mayer et al, 2024).

In brief, cells were resuspended in 0.1 M sodium cacodylate, 1 mM CaCl_2_, 3% glutaraldehyde, pH 7.2, subjected to microwave treatment (40 s, 750 W, max. 30 °C; PELCO BioWave Pro + , Ted Pella, Redding, CA, USA), incubated for 5 min at room temperature, and again subjected to microwave treatment. Cells were washed two times in 0.1 M sodium cacodylate, 1 mM CaCl_2_, resuspended in 4% KMnO_4_, subjected to microwave treatment, washed in 4% KMnO_4_, and again subjected to microwave treatment. Cells were washed six times in water, incubated for 15 min at room temperature in 0.5% NaIO_4_, and washed three times in water. The cell pellet was embedded in the same volume of 2% agar noble, cut into small pieces (about 1 mm^3^), and washed with water. For dehydration, the sample was transferred to 50% acetone and subjected to microwave treatment (40 s, 750 W, max. 37 °C). Microwave treatment was repeated with increasing concentrations of acetone: 70%, 95%, and two times 100%. For Epon infiltration, the sample was transferred into a 1:1 acetone/Epon mixture and subjected to microwave treatment (5 min, 450 W, max. 42 °C, 20“Hg vacuum). Microwave treatment was repeated two times in pure Epon. Epon polymerization was for 12 h at 60 °C. Contrast enhancement of ultrathin sections (60–70 nm) placed on Pioloform-coated copper grids was performed with 2% uranyl acetate for 15 min at room temperature, followed by three times washing with water for 2 min, incubation with lead citrate for 2 min, and three times washing with water for 2 min. Electron microscopy was performed with a JEOL JEM-1400 Plus transmission electron microscope operated at 80 kV, a JEOL Ruby CCD camera (3296 × 2472 pixels) and the TEM Center software Ver.1.7.19.2439 (JEOL, Tokyo, Japan). Adobe Photoshop CS6 (Adobe Inc., San Jose, CA, USA) was used for linear adjustments of brightness and contrast.

### Proteomics

Prior to mass spectrometric analysis, proteins were precipitated using acetone precipitation. Briefly, protein lysates were mixed with eight volumes of acetone and one volume of methanol. After incubation overnight at −20 °C, the protein was pelleted by centrifugation at 2500 × *g* for 20 min at 4 °C and washed once with 80% acetone. Protein pellets were air-dried and resuspended in denaturation buffer. Subsequently, protein quantification was performed using the Bradford assay. Absorbance was measured at 595 nm, and concentrations were interpolated using a standard curve of BSA. For in-solution digestion, 10 µg of each sample was diluted to a concentration of 1 mg/mL with denaturation buffer. Cysteine bonds were reduced by incubation with 1 mM DTT for 1 h at RT before carbamidomethylation by incubation with 550 nM IAA for 1 h at RT in the dark. Predigestion was performed with LysC for 3 h at RT, followed by digestion with Trypsin overnight at RT. The reaction was quenched by the addition of 1% TFA, and peptides were purified using StageTips (Rappsilber et al, 2007).

Mass spectrometric analysis was performed using an Orbitrap Exploris 480 online coupled to an EASY nLC-1200 system (Thermo Fisher Scientific). Separation of peptides was performed using a 20 cm HPLC column with 75 µm inner diameter (CoAnnTech) in-house packed with 1.9 µm ReproSil-Pur C18 -AQ silica beads (Dr. Maisch GmbH) and elution by solvent B in a 60-min linear gradient from 5 to 33% at a flow rate of 200 nL/min. Thirty-minute wash runs were performed after each sample to minimize carry-over. Ionization was achieved by ESI, and the mass spectrometer was operated in positive ion mode controlled by XCalibur (Thermo Fisher Scientific).

Spectra were acquired with a scan range of 350–950 *m/z* and a resolution on the MS1 level of 60,000. MS2 scans were performed using DIA isolation windows of 8 *m/z* with an overlap of 1 *m/z* (75 isolation windows), HCD collision energy of 30%, and resolution of 15,000.

Raw files were processed using Spectronaut version 18 in directDIA+ mode using default settings (Bruderer et al, 2017). Spectra were predicted using a Uniprot *Saccharomyces cerevisiae* database with the TIR protein added by hand (53,563 entries, downloaded on 2022/12/16). The data were exported using the protein pivot report using PG.ProteinGroups as a unique protein ID and the PG.Quantity as a quantification column. Downstream data analysis was performed using Perseus version 2.0.5.0 (Tyanova et al, 2016) and Microsoft Excel. Protein functions and compartments were annotated using Gene Ontology Biological Processes and Gene Ontology Cellular Compartment databases as well as Saccharomyces Genome Database for mitochondrial sub-compartments all downloaded on 2023/12/12. Statistical analysis of proteomes of 0 h vs. Control and 21 h vs. *Δfzo1* was done by Student’s *t* test. Hierarchical clustering of important processes altered by Fzo1 depletion was done after filtering for the selected GO terms using z-scores while preserving the order of rows as well as columns (supervised hierarchical clustering).

### Model construction and simulation

The aim of our simulation was to test whether unequal segregation and reduced synthesis of mitochondrial DNA are sufficient to explain the rapid loss of mtDNA upon Fzo1 depletion qualitatively, i.e., on a time-scale that roughly matches the experimental observations. The strategy to design this “toy model” was therefore to keep it as simple as possible while including sufficient detail to (1) obtain a stable distribution of mtDNA for the wild-type condition on the time-scale of the simulation, and (2) capture the major differences between wild-type and mutant cells observed experimentally.

Model simulations were performed in MATLAB R2017a (MathWorks). Each simulation was initiated with a population of 10,000 cells, each of which contained 16 nucleoids, and each was about to divide. At division, each cell distributed its nucleoids between the bud and the mother cell with a ratio that was randomly drawn from a log-normal distribution (distributed nucleoids were rounded to integers). The parameters for the log-normal distribution were different depending on whether wild-type or Fzo1-depleted cells were simulated and were determined from experiments by fitting with the Matlab Lognormal distribution function (Fig. EV8A). Specifically, for wildtype we used experimental measurements of the entire experiment, while for Fzo1-depleted cells we included data from 3 to 9 h after onset of depletion.

To keep the number of cells constant throughout the simulation, after cell division, the number of total cells was reduced by 50% to 10000 at random. To simulate mtDNA synthesis during the cell cycle, the number of nucleoids in each cell was increased by a factor that depends on whether the nucleoid number in the cell is very low (*n *< 4), low (4 = <*n* < 7), medium (7 = <*n* < 10), high (10 = <*n* < 13), or very high (*n* > 13). The corresponding parameters were again determined from experiments and were different for wild-type (entire experiment) and Fzo1-depleted cells (3–9 h after onset of depletion). Note that including this mtDNA-dependent synthesis rate in the model was necessary to ensure a stable distribution in the wild-type condition (Fig. EV8E). The choice of five categories led to a stable wild-type simulation while at the same time ensuring a sufficient number of cells in each category to determine the parameters experimentally for both wild-type and mutant cells. Specifically, to categorize cells from the experiment, we measured the Atp6 amount at birth. The cells were then categorized accordingly: for example, cells with less than 3.5/8 of the mean were categorized as “very low”. This was done because the mean amount in the population was assumed to correspond to cells that are born with 8 nucleoids. Note that we chose the ranges of these categories for the continuous values obtained from experiments in such a way that, through nearest-integer rounding, they most appropriately reflect the discrete nucleoid values in the model. We then measured the mean relative Atp6 synthesis rate across all cells in the category (Fig. EV8B). After simulating synthesis, nucleoid numbers were again rounded to integers. Continuing with the simulation of the next cell division, the entire simulation was repeated 100 times.

Note that we did not consider in the simulation any variability in cell size or cell cycle duration. To test whether our simulation results are robust to changes in the initial distribution of nucleoids, we repeated it with normally distributed nucleoid numbers at time 0, mimicking the experimentally determined distribution at cell birth, and found no qualitative differences. We also ensured that the simulation results are qualitatively robust to moderate changes to the number of initial nucleoids (Fig. EV8C, adjusting the categories for the mtDNA synthesis accordingly) or the width of log-normal distributions setting segregation at inheritance (lower or higher sigmas, Fig. EV8D). Finally, we replaced the log-normal distributions with Gamma distributions, leading again to the same conclusions (Fig. EV8F).

## Supplementary information

Appendix

Peer Review File

Dataset EV1

Movie EV1

Movie EV2

Movie EV3

Movie EV4

Movie EV5

Source data Fig. 1

Source data Fig. 2

Source data Fig. 3

Source data Fig. 4

Source data Fig. 5

Source data Fig. 6

Source data Fig. 7

Source data Fig. 8

Expanded View Figures

## Data Availability

The datasets and computer code produced in this study are available in the following databases: Raw and processed images and related data files have been uploaded to the University of Tübingen’s OMERO repository (Burel et al, 2015). All data can be accessed and downloaded by requesting a guest account from the Quantitative Biology Center (QBiC) at the University of Tuebingen via email (support@qbic.zendesk.com) for project ID: QBIOIMG0002. https://proxy.qbic.uni-tuebingen.de/webclient/?show=project-251 provides a direct link once an account has been installed. The mass spectrometry proteomics data have been deposited to the ProteomeXchange Consortium via the PRIDE (Deutsch et al, 2023; Perez-Riverol et al, 2025) partner repository with the dataset identifier PXD063340. The code for the model in Fig. 8 has been deposited on GitHub. https://github.com/SchmollerLab/Fzo1Model. The source data of this paper are collected in the following database record: biostudies:S-SCDT-10_1038-S44319-026-00794-5.
