# Supplementary material for: In the absence of mitochondrial fusion unequal segregation of mitochondria drives mtDNA loss
Source: EMBO Rep. 2026 May 14;27(12):3359–93. doi: 10.1038/s44319-026-00794-5 (PMC13303861; doi:10.1038/s44319-026-00794-5)
Supplement: Supplementary file 1 — Appendix [file 44319_2026_794_MOESM1_ESM.pdf]

Appendix File for:

In the absence of mitochondrial fusion unequal segregation of mitochondria drives mtDNA loss

Lisa Dengler[1], Francesco Padovani[2], Bianca Lemke[3], Rebecca Brugger[4], Alissa Benedikt[2], Benedikt Westermann[4], Boris Maček[3], Kurt M. Schmoller[2], Jennifer C. Ewald[1*]

[1]Molecular Cell Biology, Institute of Cell Biology, University of Tübingen, Germany
[2]Institute of Functional Epigenetics, Molecular Targets and Therapeutics Center, Helmholtz Zentrum München, Germany
[3]Quantitative Proteomics, Institute of Cell Biology, University of Tübingen, Germany
[4]Cell Biology, University of Bayreuth, Germany
*corresponding author: jennifer-christina.ewald@uni-tuebingen.de

Appendix Figures

Appendix Figure S1: z-scores of the individual mitochondrial sub-compartments of the proteomics measurements....................2

Appendix Figure S2: IQR of the mitochondrial concentration related to Figure 3A and cell sizes and mitochondrial amounts related to Figure 4F....................3

Appendix Figure S3: Uncropped Western Blots....................4

Appendix Tables

Appendix Table S1: Strains used in this study....................5

Appendix Table S2: Plasmids used in this study....................7

Appendix Table S3: Plasmids used in this study....................8

Appendix Table S4: Optical filters for microscopy....................9

Appendix Table S5: Exposure settings and intensities....................9

Appendix Table S6: Primers used for DNA-qPCR....................10

Appendix Table S7: Primers used for RT-qPCR....................10

Appendix References....................11

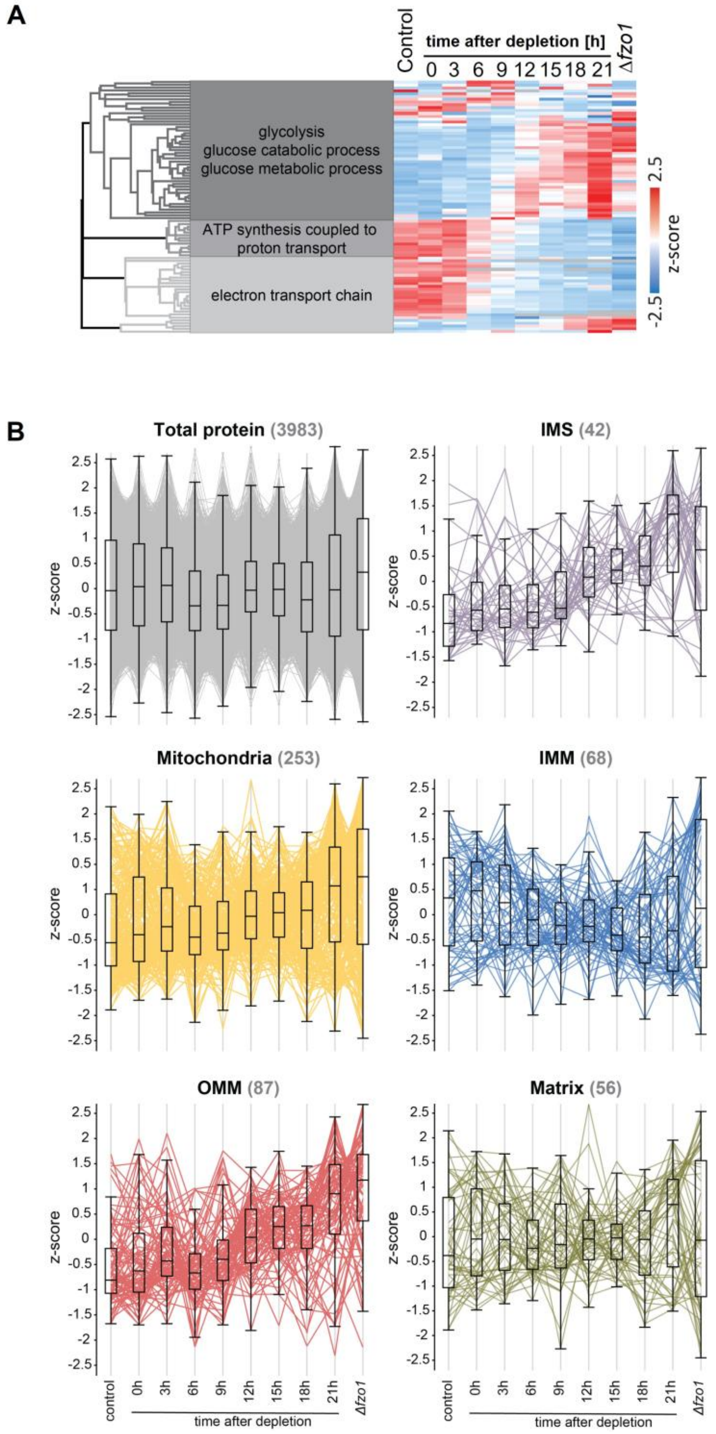

Appendix Figure S1: A) Heat map of important processes which are altered through Fzo1 depletion. B) Z-scores of proteomic measurements of the total protein and the different mitochondrial sub-compartments from three biological replicates. Boxplots are presented using the Tukey method, line indicates median, boxes show 25th and 75th percentiles, whiskers show 10th and 90th percentiles.

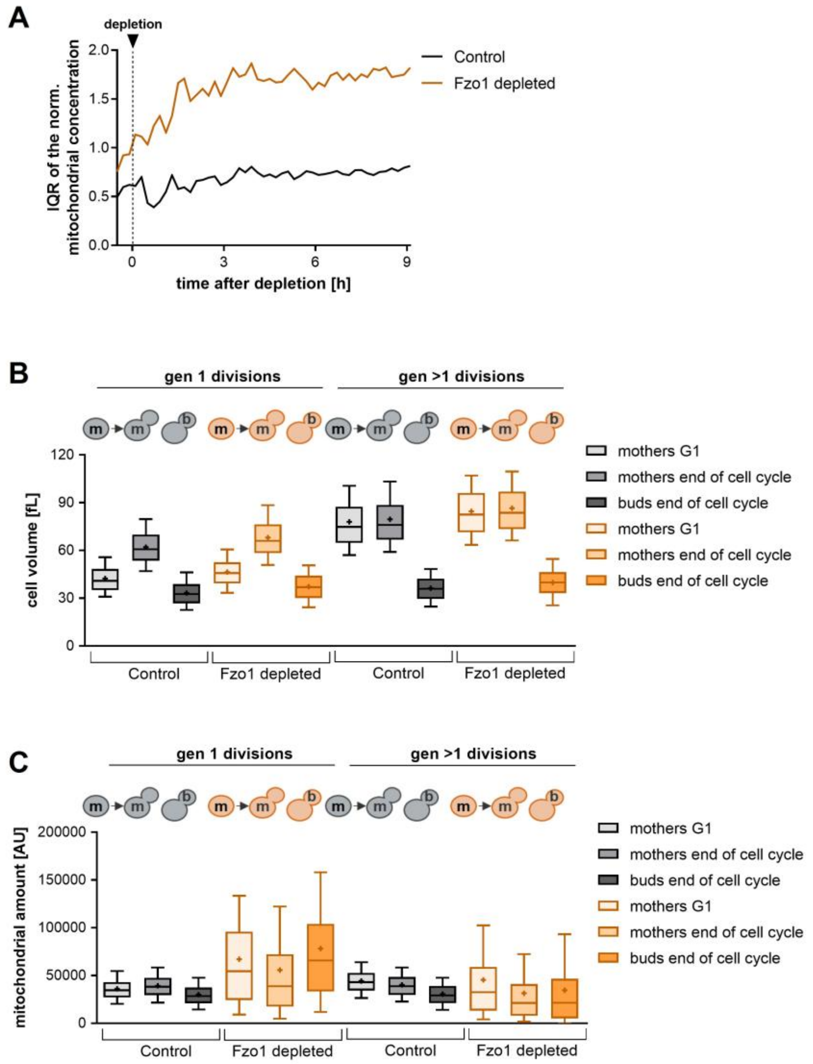

Appendix Figure S2: A) Interquartile range (IQR) of the mitochondrial concentration of Figure 3A. Data from two biological replicates with 1785 Control and 2075 Fzo1 depleted cells at 9 h is shown. B) Cell sizes and C) mitochondrial amounts related to Figure 4F. Control: n (gen 1) = 544, n (gen>1) = 706; Fzo1 depleted: n (gen 1) = 619, n (gen>1) = 790. Boxes indicate median, 25th, and 75th percentiles; + indicate means; whiskers indicate 10th and 90th percentiles.

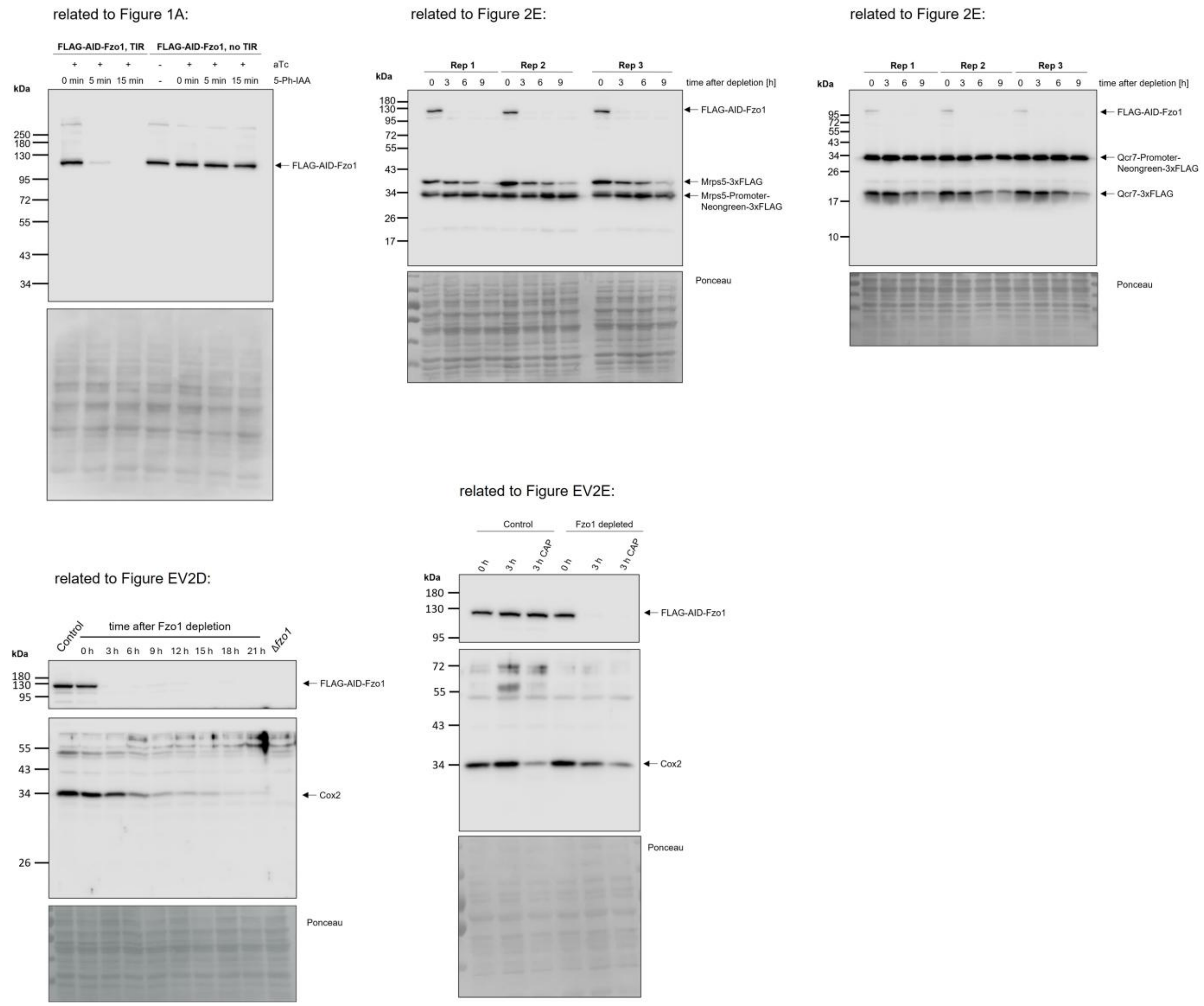

Appendix Figure S3: Uncropped Western Blots used in the indicated figures.

Appendix Table S1. Strains used in this study. All strains are W303 derivatives. All strains were constructed in this study.

Name	Genotype	Description	Figures
LD137-1	*Mat a, leu2-3,112:: TetR-LEU, can1-100 ura3-1::URA3-Tet-pr-OsTIR1F74G, his3-11,15::His3*	Tetracycline inducible TIR, based on (Azizoğlu *et al*, 2023)	Fig EV1D,E
LD138-1	*Mat a, LEU, can1-100 ura3-1::URA, his3-11,15::HIS3*	Wild-type	Fig EV1D,EV1E, 2H, EV2J
LD162-1	*Mat a, leu2-3,112:: TetR-LEU, can1-100 ura3-1::URA3-Tet-pr-OsTIR1F74G, his3-11,15::HIS3, FLAG-AID-Fzo1*	Tetracycline inducible TIR, FLAG-AID degron (Yesbolatova *et al*, 2020)	Fig 1A, 1D, EV1D,E,F, 2A,B,D,G,H, EV2A,B,C,D,F,G, H,I,K, S1A,B
LD146-1	*Mat a, LEU, can1-100 ura3-1::URA, his3-11,15::HIS3, Fzo1::HygMX*	Fzo1 deletion	Fig 1D, 2H, EV2D,H,I,J. S1A,B
LD181-1	*Mat a, LEU, can1-100 ura3-1::URA, his3-11,15::His3, 3xFLAG-AID-Fzo1*	FLAG-AID degron	Fig 1A, EV1D
LD219-1	*Mat a, leu2-3,112:: TetR-LEU, can1-100 ura3-1::URA3-Tet-pr-OsTIR1F74G, his3-11,15::HIS3, FLAG-AID-Fzo1, Pdr5::HphMX*	Tetracycline inducible TIR, FLAG-AID, Pdr5 deletion	Fig 2C, EV2E,
LD188-1	*Mat a, leu2-3,112:: TetR-LEU, can1-100 ura3-1::URA3-Tet-pr-OsTIR1F74G, his3-11,15::TEF-pr-preSu9-mCardinal-His5*	Tetracycline inducible TIR	Fig EV1B,C,
LD190-1	*Mat a, leu2-3,112:: TetR-LEU, can1-100 ura3-1::URA3-Tet-pr-OsTIR1F74G, his3-11,15::TEF-pr-preSu9-mCardinal-His5, FLAG-AID-Fzo1*	Tetracycline inducible TIR, FLAG-AID degron, preSu9-mCardinal	Fig EV1A,B,C,G EV3I, 4A-H EV4A,B,C,D
LD195-1	*Mat a, leu2-3,112:: LEU2, can1-100 ura3-1::URA3, his3-11,15::TEF-pr-preSu9-mCardinal-His5, Fzo1::HygMX*	Fzo1 deletion, preSu9-mCardinal	Fig EV1B,C,G, 4A, EV4A,B

LD206-1	*Mat a, leu2-3,112:: LEU2, can1-100 ura3-1::LexA-ER-AD-LexA-Pr-OsTIR1F74G-Ura3, his3-11,15::TEF-pr-preSu9-mCardinal-His5, 3x-FLAG-AID-Fzo1, TEFpr-preCox4-NG-HO-homology-KanMX*	Estradiol inducible TIR, FLAG-AID, MitoLoc reporter to estimate protein import changes, modified from (Vowinckel *et al*, 2015)	Fig 3A,C,D, EV3D,E,F,G, 4I, EV4E,G,H
KK124-1	*Mat a, leu2-3,112:: LEU2, can1-100 ura3-1::URA3, his3-11,15::TEF-pr-preSu9-mCardinal-His5, TEFpr-preCox4-NG-HO-homology-KanMX, Fzo1::HygMX*	Fzo1 deletion, MitoLoc reporter to estimate protein import changes	Fig EV3F
LD208-1	*Mat alpha, ade2-1 ::Ade2, his3-11,15::TEF-pr-preSu9-mCardinal-His5, trp1-1::Trp1, leu2-3,112::Leu2, ura3-1::LexA-ER-AD-LexA-Pr-OsTIR1F74G-Ura3, can1-100, ATP6-NG, 3xFLAG-AID-Fzo1*	Atp6-NG (Jakubke *et al*, 2021), preSu9-mCardinal, Estradiol inducible TIR, FLAG-AID degron	Fig 1B,C, EV3A,B EV4F, 5B,C,D EV5B,C,D,F, 6A-D, EV6A-F, 7A-G, EV7A-C, EV8A,B
LD209-1	*Mat alpha, ade2-1 ::Ade2, his3-11,15::TEF-pr-preSu9-mCardinal-His5, trp1-1::Trp1, leu2-3,112::Leu2, ura3-1::LexA-ER-AD-LexA-Pr-OsTIR1F74G-Ura3, can1-100, ATP6-NG, 3xFLAG-AID-Fzo1, Atg32::HphMX*	Atp6-NG, preSu9-mCardinal, Estradiol inducible TIR, FLAG-AID degron, Atg32 deletion	Fig EV5C,D
LD222-1	*Mat a, leu2-3,112:: TetR-LEU, can1-100 ura3-1::URA3-Tet-pr-OsTIR1F74G, his3-11,15::HIS3, FLAG-AID-Fzo1, Mrps5-3xFLAG-HphMX, Mrps5-Pr-Neongreen-FLAG-KanMX*	Tetracycline inducible TIR, FLAG-AID, Mrps5-3xFLAG, Mrps5-Promoter-reporter	Fig 2E,F
LD224-1	*Mat a, leu2-3,112:: TetR-LEU, can1-100 ura3-1::URA3-Tet-pr-OsTIR1F74G, his3-11,15::HIS3, FLAG-AID-Fzo1, Qcr7-3xFLAG-HphMX, Qcr7-Pr-Neongreen-FLAG-KanMX*	Tetracycline inducible TIR, FLAG-AID, Qcr7-3xFLAG, Qcr7-Promoter-reporter	Fig 2E,F
KK144-1	*Mat a, leu2-3,112:: TetR-LEU, can1-100 ura3-1::URA3-Tet-pr-*	Tetracycline inducible TIR,	Fig 5A, EV5B,E,F

	OsTIR1F74G, his3-11,15::HIS3, FLAG-AID-Fzo1, HO-su9-mscarlet-i3-KanMX-kaede-HINESS	FLAG-AID, HINESS system (Deng *et al*, 2025)	
KK149-1	*Mat a, leu2-3,112:: TetR-LEU, can1-100 ura3-1::URA3-Tet-pr-OsTIR1F74G, his3-11,15::HIS3, FLAG-AID-Fzo1, Tom70-NG-NatMX*	Tetracycline inducible TIR, FLAG-AID, Tom70-Neongreen	Fig EV3H
KK150-1	*Mat a, leu2-3,112:: TetR-LEU, can1-100 ura3-1::URA3-Tet-pr-OsTIR1F74G, his3-11,15::TEF-pr-preSu9-mCardinal-His5, FLAG-AID-Fzo1, Tom70-NG-NatMX*	Tetracycline inducible TIR, FLAG-AID, preSu9-mCardinal, Tom70-Neongreen	Fig EV3C

Appendix Table S2. Plasmids used in this study.

Name	Description	Origin
pLHG003	Tet-Promoter-OsTIRF74G-URA3	This study
FRP2061	Estradiol inducible Cas9-KanMX	(Azizoğlu *et al.*, 2023)
FRP2100	Nat helper plasmid	(Azizoğlu *et al.*, 2023)
FRP2370	Tet-repressor-LEU2	(Azizoğlu *et al.*, 2023)
pLD036	TEFpr-preSu9-mCardinal-His5	This study
pLD038	TEFpr-preCox4-Neongreen-HO homology	This study
pLD039	LexA transcriotion factor with Estradiol-Promoter-OsTIRF74G-URA3	This study
pLD043	Atp17-Promoter-Neongreen-FLAG-HO-homology-KanMX	This study
pLD044	Mrps5-Promoter-Neongreen-FLAG-HO-homology-KanMX	This study
pLD046	Qcr7-Promoter-Neongreen-FLAG-HO-homology-KanMX	This study
pFP002	preSu9-mScarlet-i3-Kaede-HI-NESS-HO-homology-KanMX	This study, based on (Deng *et al.*, 2025)

Appendix Table S3. Components of 1x Synthetic complete (SC).

component	g/L	[mM] final
Adenine	0.031	0.228
L-Arg (HCl)	0.021	0.098
L-Aspartic acid	0.103	0.773
L-Glutamic acid	0.081	0.548
L-His	0.021	0.133
L-Leu	0.123	0.941
L-Lys (HCl)	0.031	0.169
L-Met	0.021	0.138
L-Phe	0.051	0.311
L-Ser	0.386	3.670
L-Thr	0.206	1.727
L-Tyr	0.031	0.170
L-Trp	0.041	0.201
L-Val	0.154	1.317
Uracil	0.021	0.184

Appendix TableS4. Optical filters for epifluorescence microscopy.

Filters used for imaging on a Nikon Ti2-E epifluorescence microscope. All described filters are manufactured by Chroma and purchased from AHF.

Fluorophore	LED Wave-length	Filter Set	Excitation Filter	Dichroic	Emission Filter
mNeongreen, Kaede	513 nm	YFP ET Filter Set	ET500/20x	T515lp, Di 25 mm x 36 mm	ET535/30m
mCardinal, TMRM	575 nm	585/29 BrightLine HC<br>650/60 BrightLine HC<br>Beamsplitter T610 LPXR	ET585/29x	T610 LPXR, Di 25 mm x 36 mm	ET650/60
DAPI	390 nm	DAPI/FITC/Cy3 /Cy5<br>Quad LED ET Set	Quadband Exciter ET391/479 /554/638	Beamsplitter (89402bs), Di 25 mm x 36 mm	ET435/520<br>/595/695

Appendix TableS5. Exposure times and intensities for epifluorescence microscopy.

Fluorophore	Imaged protein	Intensity	Exposure time
mCardinal	preSu9	5 %	100 ms
mNeongreen	preCox4	10 %	200 ms
mNeongreen	Atp6	10 %	300 ms
mKaede	HINESS	15 %	300 ms
mNeongreen	Tom70	15 %	500 ms
TMRM	MMP	30 %	300 ms
DAPI	nucleoids	26 %	500 ms

Appendix Table S6. Primers used for DNA-qPCR. All Primers were purchased from Integrated DNA Technologies (IDT).

Name	Sequence
COX1_fw	CAACGGGGACAATAGCATGC
COX1_rev	CGGACAGTTCTTACCTTGCG
COB1_fw	AAATTGGAGCATGCCATGTA
COB1_rev	AGCGATTTGTCCCATTAAGA
COX2_fw	GTTGATGCTACTCCTGGTAGATT
COX2_rev	TTGCATGACCTGTCCCACAC
ACT1_fw	CACCCTGTTCTTTTGACTGA
ACT1_rev	CGTAGAAGGCTGGAACGTTG

Appendix Table S7. Primers used for RT-qPCR. All Primers were purchased from Integrated DNA Technologies (IDT).

Name	Sequence
COX2_fw	GTTGATGCTACTCCTGGTAGATT
COX2_rev	TTGCATGACCTGTCCCACAC
RDN18_fw	AACTCACCAGGTCCAGACACAATAAGG
RDN18_rev	AAGGTCTCGTTCGTTATCGCAATTAAGC
ACT1_fw	CACCCTGTTCTTTTGACTGA
ACT1_rev	CGTAGAAGGCTGGAACGTTG
QCR7_fw	CGTCTATTGCGAGAATTGG
QCR7_rev	CAACTCGGTTTGATGAGCCCTG
MRPS5_fw	GCGTCTTTCTATGCCTTGGT
MRPS5_rev	GATACCTGCACATTCACAAATCTC

Appendix References

Azizoğlu A, Loureiro C, Venetz J, Brent R (2023) Autorepression-Based Conditional Gene Expression System in Yeast for Variation-Suppressed Control of Protein Dosage. *Curr Protoc* 3: e647

PREPRINT: Deng J, Swift L, Zaman M, Shahhosseini F, Sharma A, Bureik D, Padovani F, Benedikt A, Jaiswal A, Brideau C *et al* (2025) A novel genetic fluorescent reporter to visualize mitochondrial nucleoids. *bioRxiv*: 2023.2010.2023.563667

Jakubke C, Roussou R, Maiser A, Schug C, Thoma F, Bunk D, Hörl D, Leonhardt H, Walter P, Klecker T *et al* (2021) Cristae-dependent quality control of the mitochondrial genome. *Science Advances* 7: eabi8886

Vowinckel J, Hartl J, Butler R, Ralser M (2015) MitoLoc: A method for the simultaneous quantification of mitochondrial network morphology and membrane potential in single cells. *Mitochondrion* 24: 77-86

Yesbolatova A, Saito Y, Kitamoto N, Makino-Itou H, Ajima R, Nakano R, Nakaoka H, Fukui K, Gamo K, Tominari Y *et al* (2020) The auxin-inducible degron 2 technology provides sharp degradation control in yeast, mammalian cells, and mice. *Nat Commun* 11: 5701
