# Supplementary material for: In the absence of mitochondrial fusion unequal segregation of mitochondria drives mtDNA loss
Source: EMBO Rep. 2026 May 14;27(12):3359–93. doi: 10.1038/s44319-026-00794-5 (PMC13303861; doi:10.1038/s44319-026-00794-5)
Supplement: Supplementary file 4 — Movie EV1 [file 44319_2026_794_MOESM4_ESM.zip › Legend_MovieEV1.docx]

**Movie EV1, related to Figure 1: Fragmentation of the mitochondrial network after Fzo1 depletion.** Fzo1-depletion was initiated at t = 0 h by addition of 2 µM 5-Ph-IAA. Pre-Su9-mCardinal was imaged every 30s using confocal microscopy. Images are maximum z-projections. Scale bar = 5 µm. LUTs were set to stay constant throughout the movie.
