# Supplementary material for: In the absence of mitochondrial fusion unequal segregation of mitochondria drives mtDNA loss
Source: EMBO Rep. 2026 May 14;27(12):3359–93. doi: 10.1038/s44319-026-00794-5 (PMC13303861; doi:10.1038/s44319-026-00794-5)
Supplement: Supplementary file 5 — Movie EV2 [file 44319_2026_794_MOESM5_ESM.zip › Legend_MovieEV2.docx]

**Movie EV2, related to Figure 5 and 6: Atp6-mNeongreen is unequally distributed and continuously lost after Fzo1 depletion.** Depletion of Fzo1 was initiated at t = 0 h by addition of 2 µM 5-Ph-IAA. Atp6-mNeongreen was imaged every 12 min using epifluorescence microscopy. Images are maximum z-projections. Scale bar = 10 µm.
