# Supplementary material for: In the absence of mitochondrial fusion unequal segregation of mitochondria drives mtDNA loss
Source: EMBO Rep. 2026 May 14;27(12):3359–93. doi: 10.1038/s44319-026-00794-5 (PMC13303861; doi:10.1038/s44319-026-00794-5)
Supplement: Supplementary file 7 — Movie EV4 [file 44319_2026_794_MOESM7_ESM.zip › Legend_MovieEV4.docx]

**Movie EV4, related to Figure 5: Nucleoids are unequally distributed and continuously lost after Fzo1 depletion.** Same video as Movie 3, with an additional overlay of pre-Su9-mScarlet-i3 (purple). Depletion of Fzo1 was initiated at t = 0 h by addition of 2 µM 5-Ph-IAA. HI-NESS-mKaede was imaged every 12 min using epifluorescence microscopy Images are maximum z-projections. Scale bar = 20 µm.
