# Supplementary material for: In the absence of mitochondrial fusion unequal segregation of mitochondria drives mtDNA loss
Source: EMBO Rep. 2026 May 14;27(12):3359–93. doi: 10.1038/s44319-026-00794-5 (PMC13303861; doi:10.1038/s44319-026-00794-5)
Supplement: Supplementary file 8 — Movie EV5 [file 44319_2026_794_MOESM8_ESM.zip › Legend_MovieEV5.docx]

**Movie EV5, related to Figure 7: Re-addition of Fzo1 enables partially recovery of the Fzo1 phenotype.** Depletion of Fzo1 was initiated at t = 0 h by addition of 2 µM 5-Ph-IAA. At t = 6 h depletion was removed by changing to medium without inducers. Atp6-mNeongreen (yellow) and pre-Su9-mCardinal (purple) were imaged every 12 min using epifluorescence microscopy. Images are maximum z-projections. Scale bar = 20 µm.
