# Supplementary figures and images for: In the absence of mitochondrial fusion unequal segregation of mitochondria drives mtDNA loss
Source: EMBO Rep. 2026 May 14;27(12):3359–93. doi: 10.1038/s44319-026-00794-5 (PMC13303861; doi:10.1038/s44319-026-00794-5)

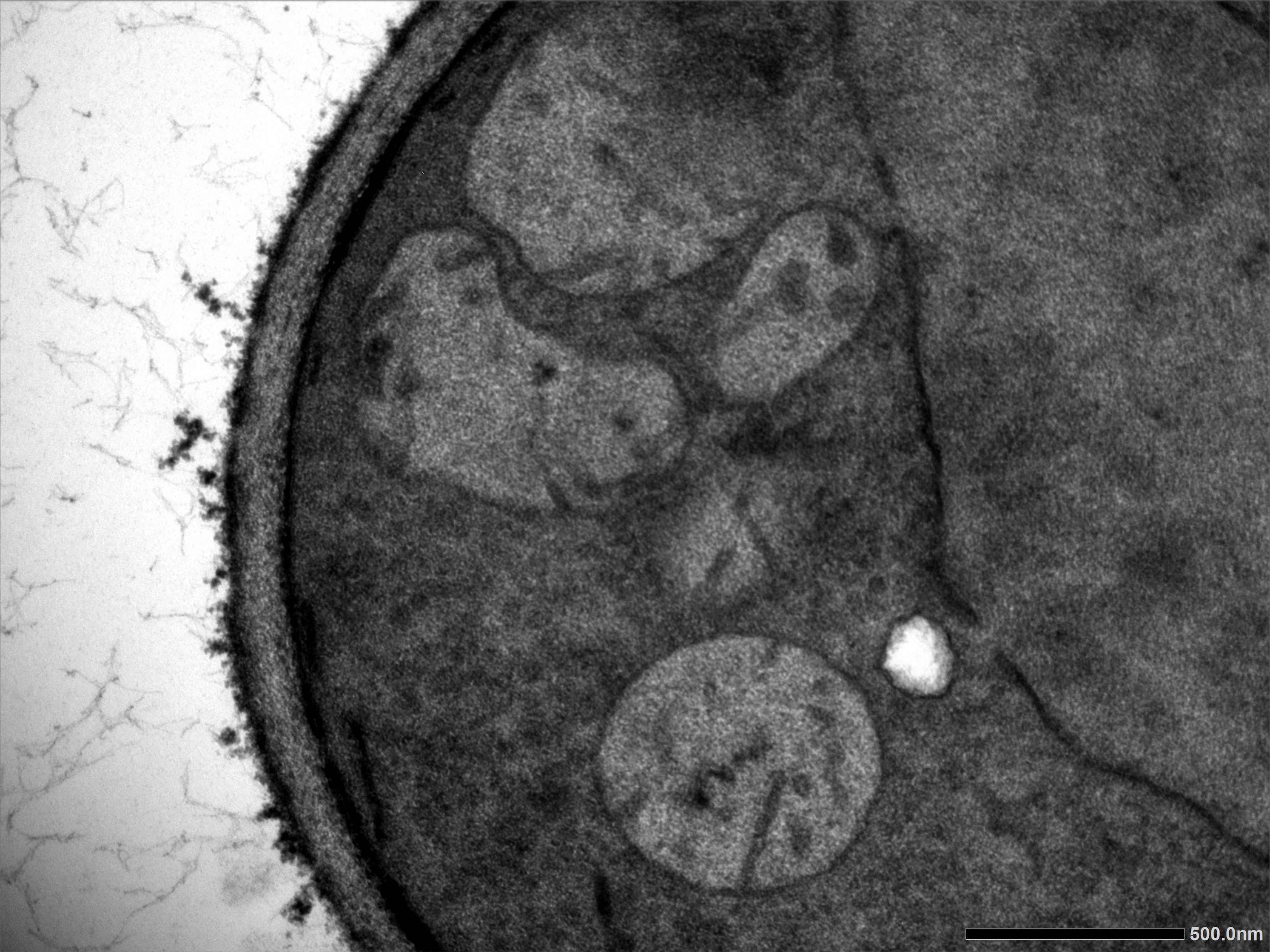

Supplement: Supplementary file 10 — Source data Fig. 2 [file 44319_2026_794_MOESM10_ESM.zip › Source Data Figure 2/2G_EM raw images/category aberrant_SA-MAG_X20k.jpg]

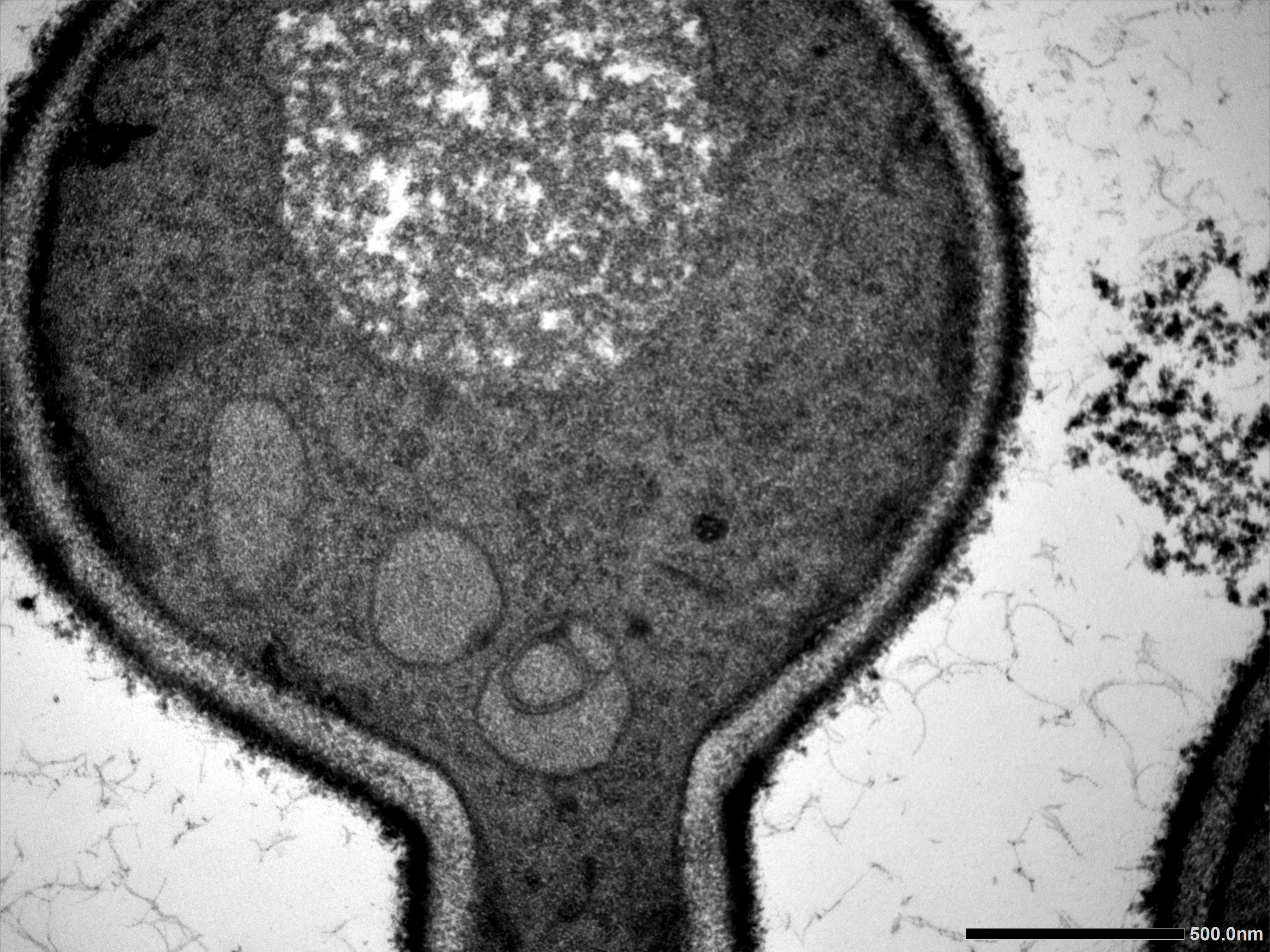

Supplement: Supplementary file 10 — Source data Fig. 2 [file 44319_2026_794_MOESM10_ESM.zip › Source Data Figure 2/2G_EM raw images/category empty_SA-MAG_X20k.jpg]

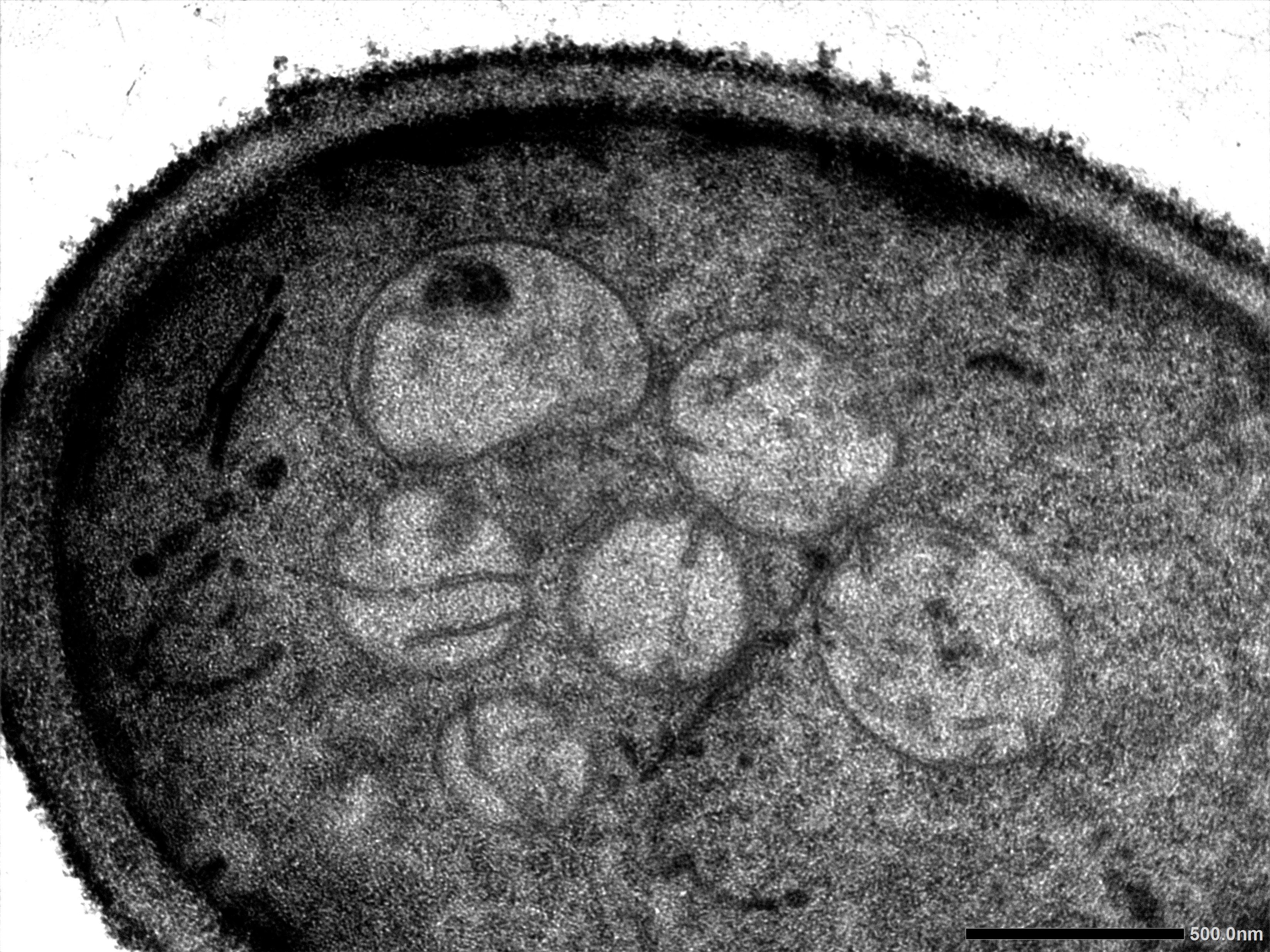

Supplement: Supplementary file 10 — Source data Fig. 2 [file 44319_2026_794_MOESM10_ESM.zip › Source Data Figure 2/2G_EM raw images/category septa_SA-MAG_X20k.jpg]

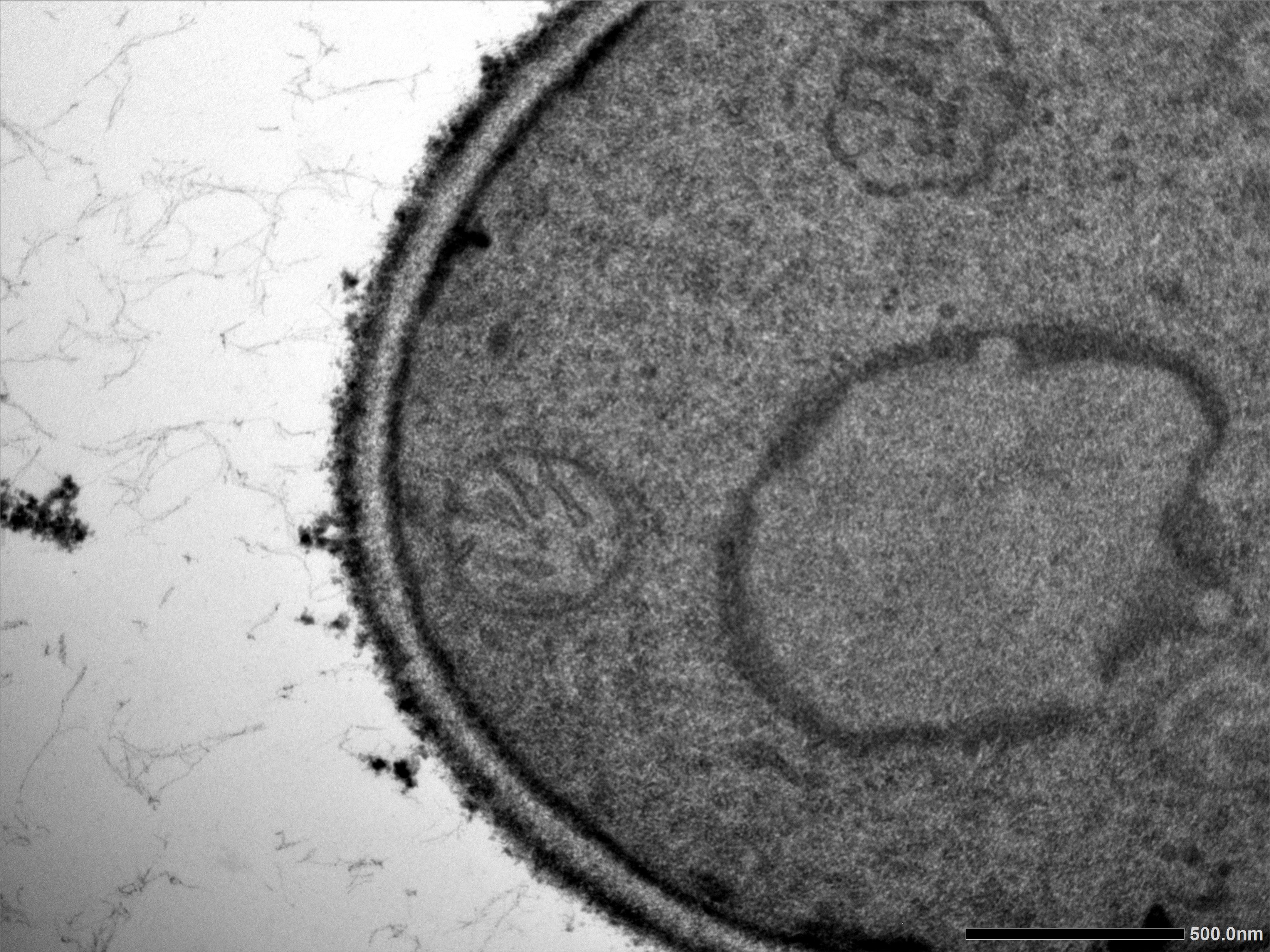

Supplement: Supplementary file 10 — Source data Fig. 2 [file 44319_2026_794_MOESM10_ESM.zip › Source Data Figure 2/2G_EM raw images/category wildtype cristae_SA-MAG_X20k.jpg]

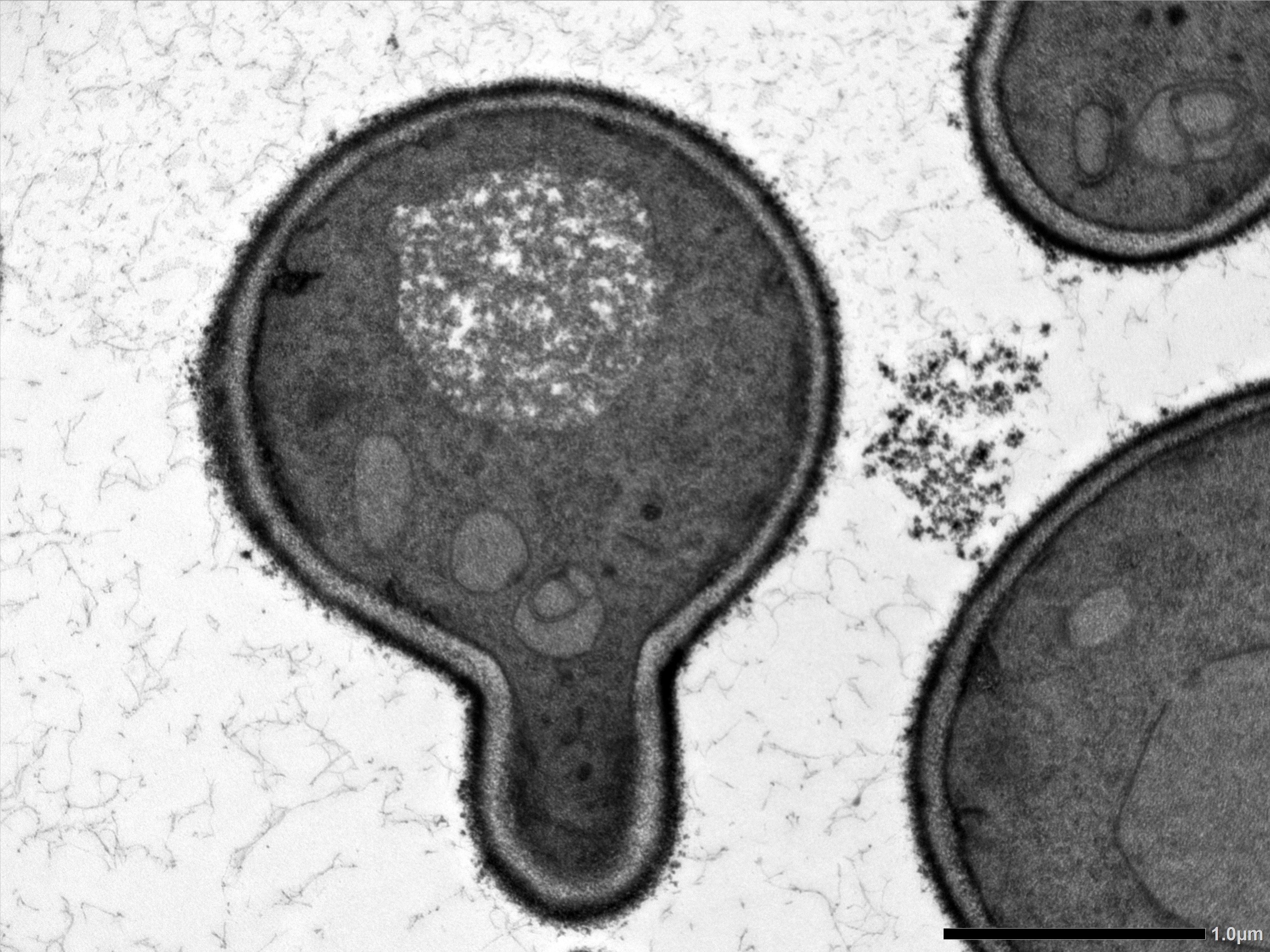

Supplement: Supplementary file 10 — Source data Fig. 2 [file 44319_2026_794_MOESM10_ESM.zip › Source Data Figure 2/2G_EM raw images/delta fzo1 example_SA-MAG_X12k.jpg]

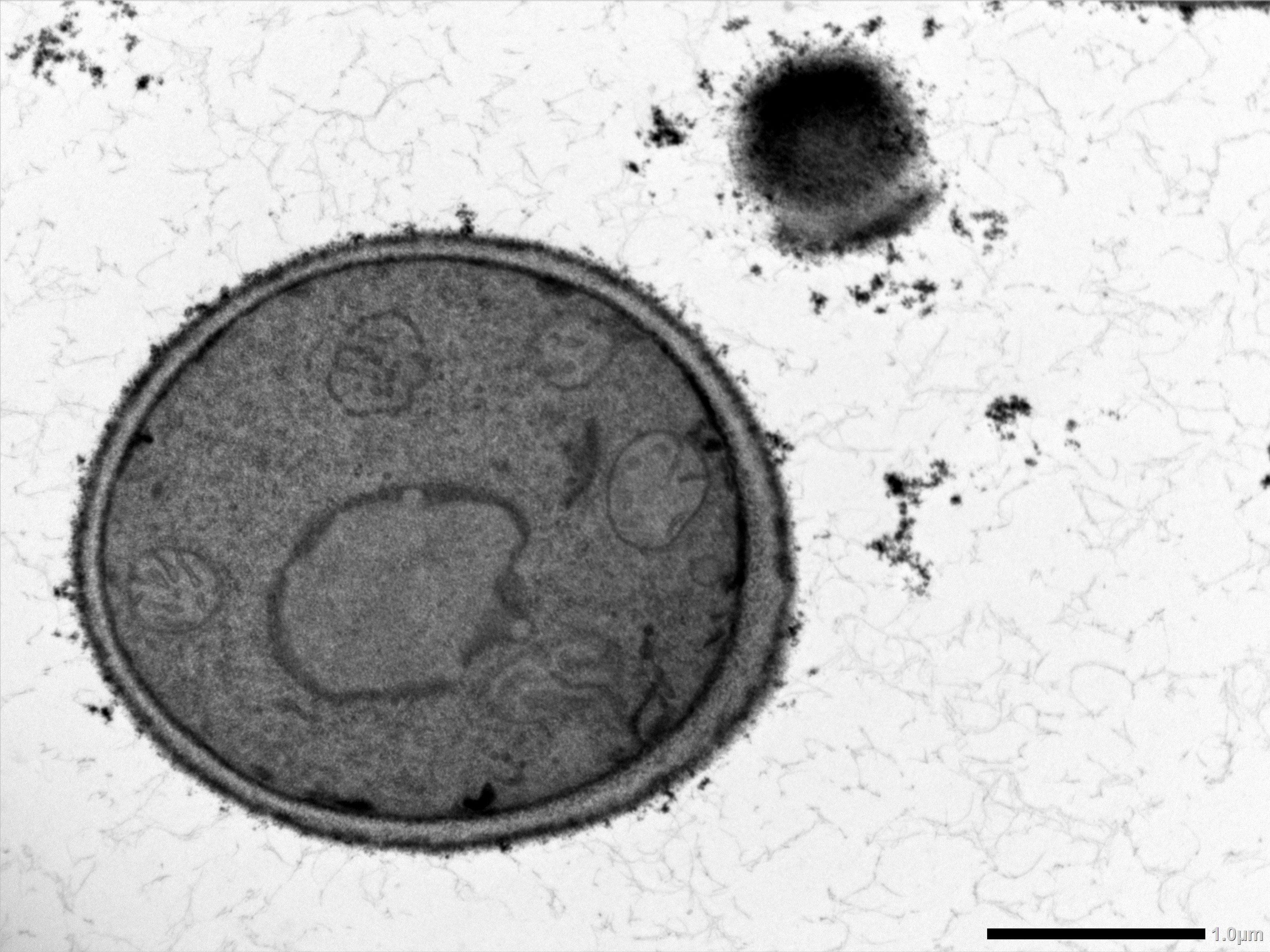

Supplement: Supplementary file 10 — Source data Fig. 2 [file 44319_2026_794_MOESM10_ESM.zip › Source Data Figure 2/2G_EM raw images/depletion t0_SA-MAG_X10k.jpg]

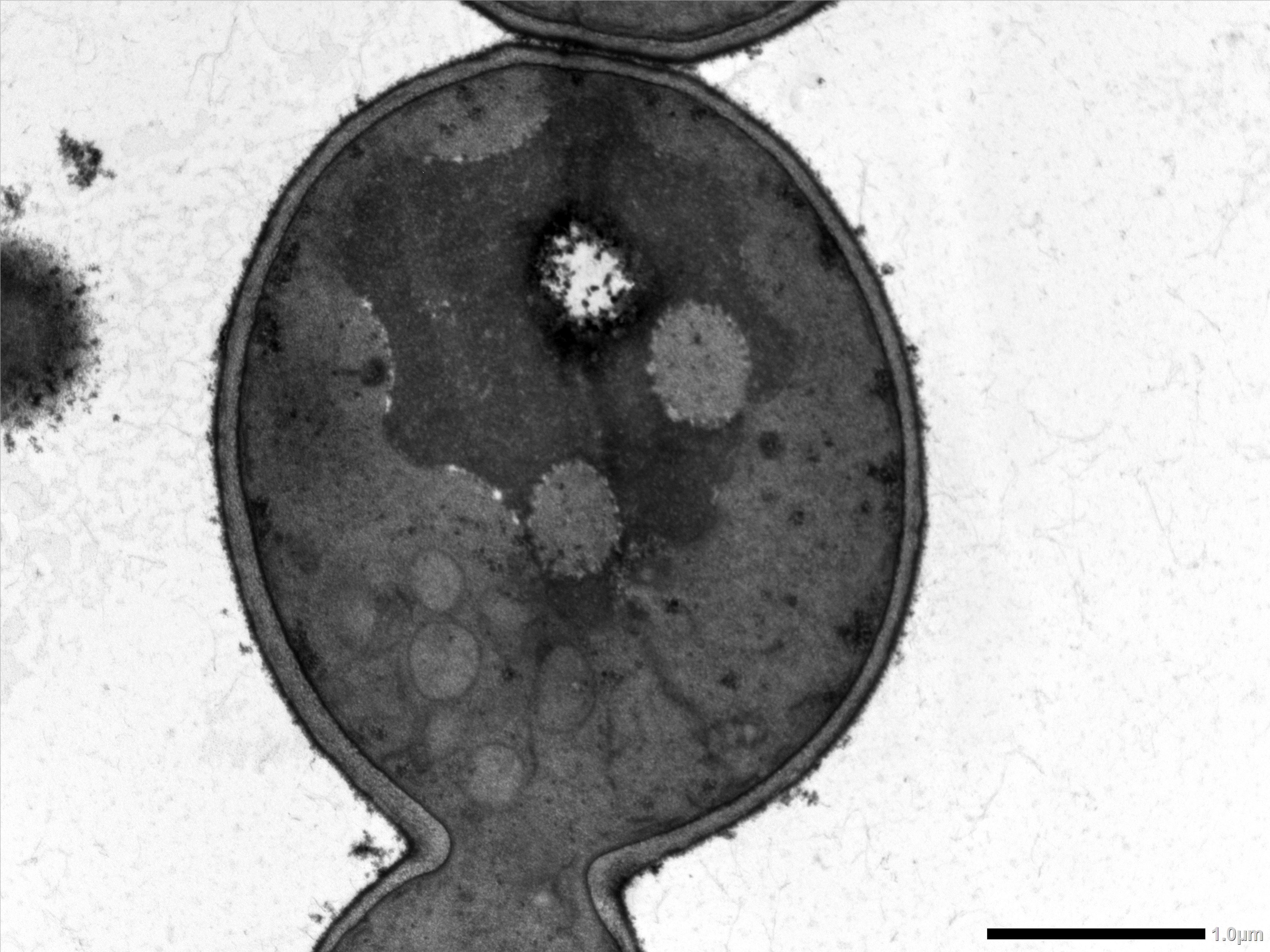

Supplement: Supplementary file 10 — Source data Fig. 2 [file 44319_2026_794_MOESM10_ESM.zip › Source Data Figure 2/2G_EM raw images/depletion t21_SA-MAG_X10k.jpg]

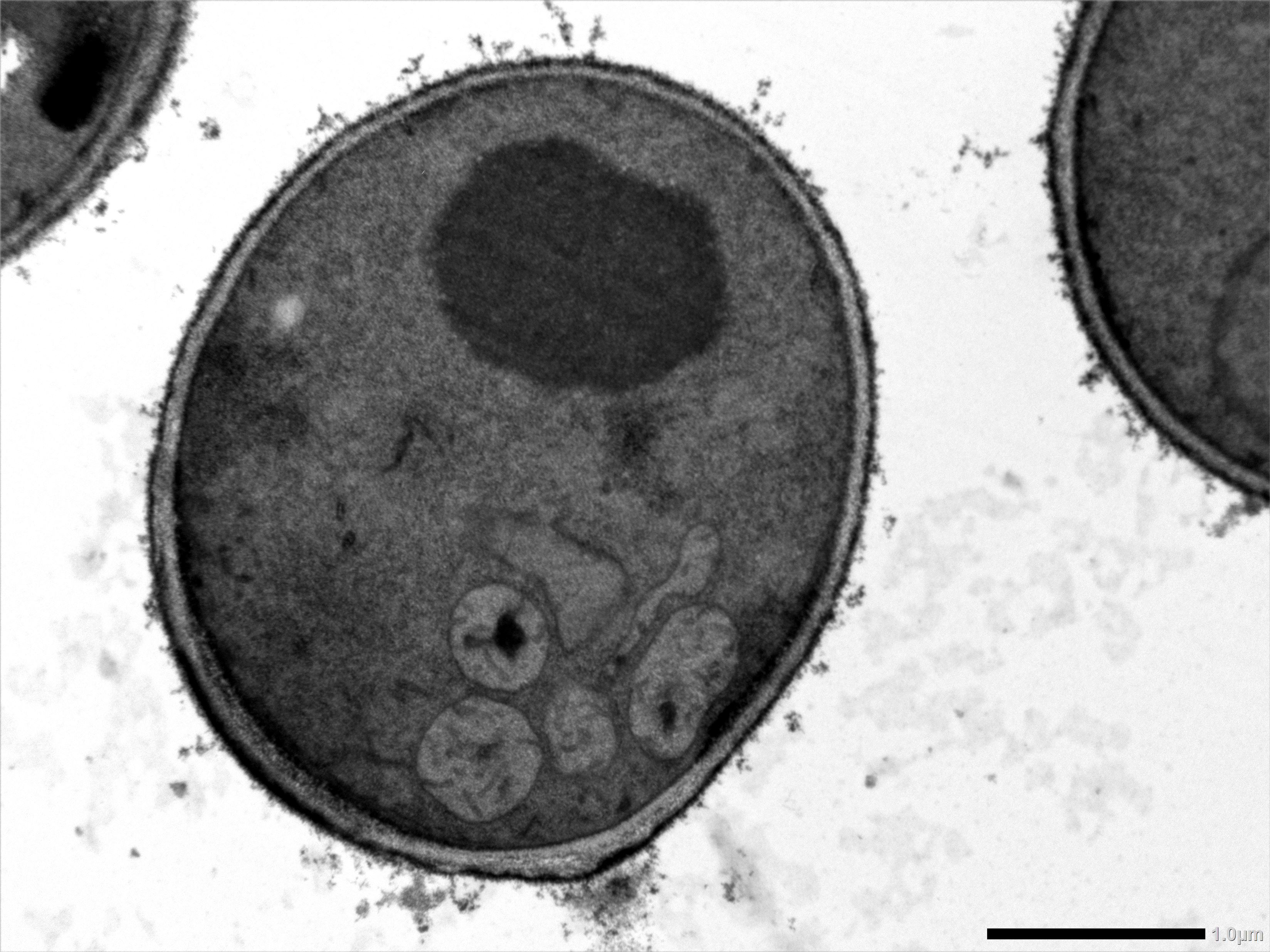

Supplement: Supplementary file 10 — Source data Fig. 2 [file 44319_2026_794_MOESM10_ESM.zip › Source Data Figure 2/2G_EM raw images/depletion t3_SA-MAG_X10k.jpg]

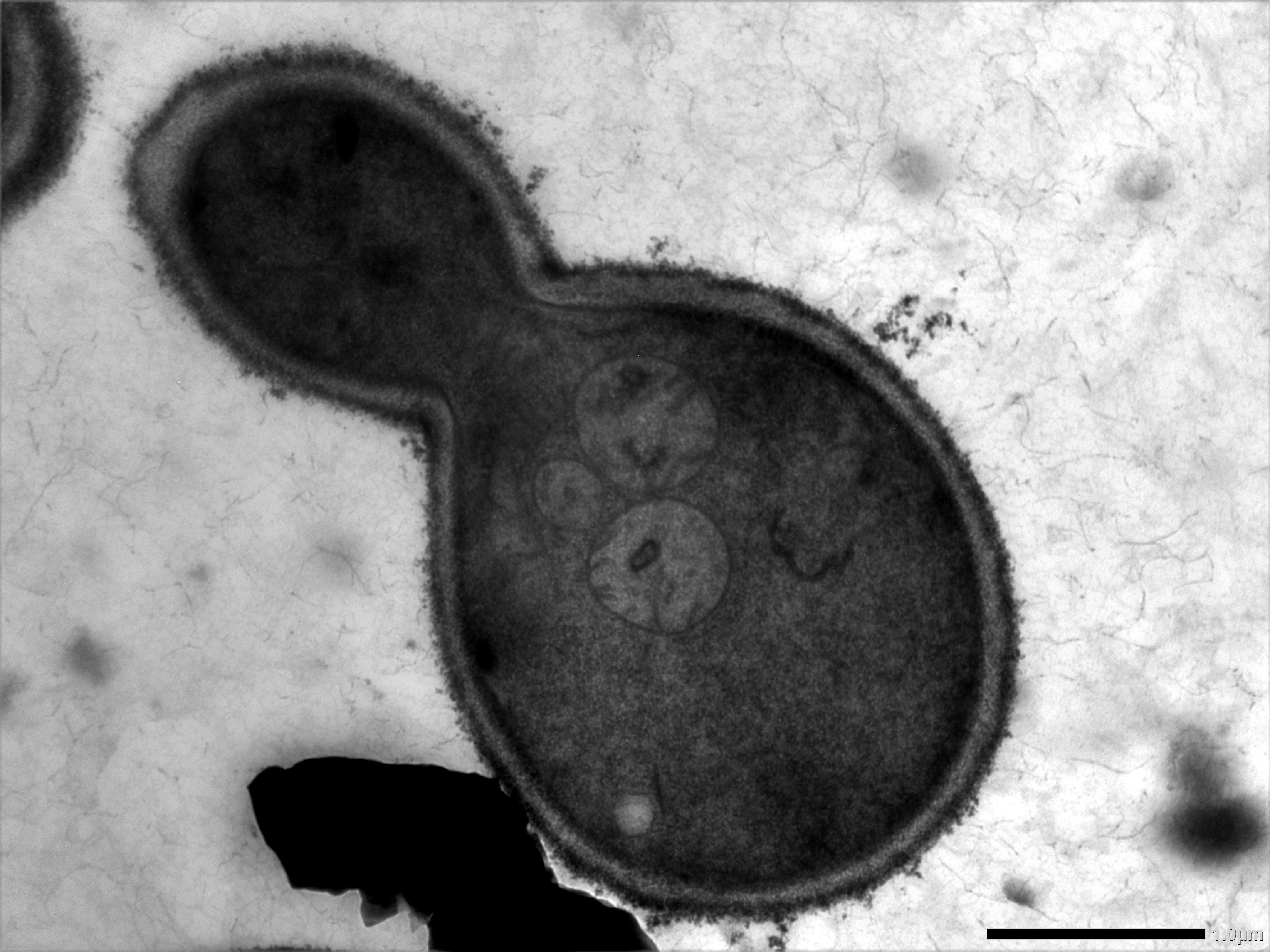

Supplement: Supplementary file 10 — Source data Fig. 2 [file 44319_2026_794_MOESM10_ESM.zip › Source Data Figure 2/2G_EM raw images/depletion t6_SA-MAG_X10k.jpg]

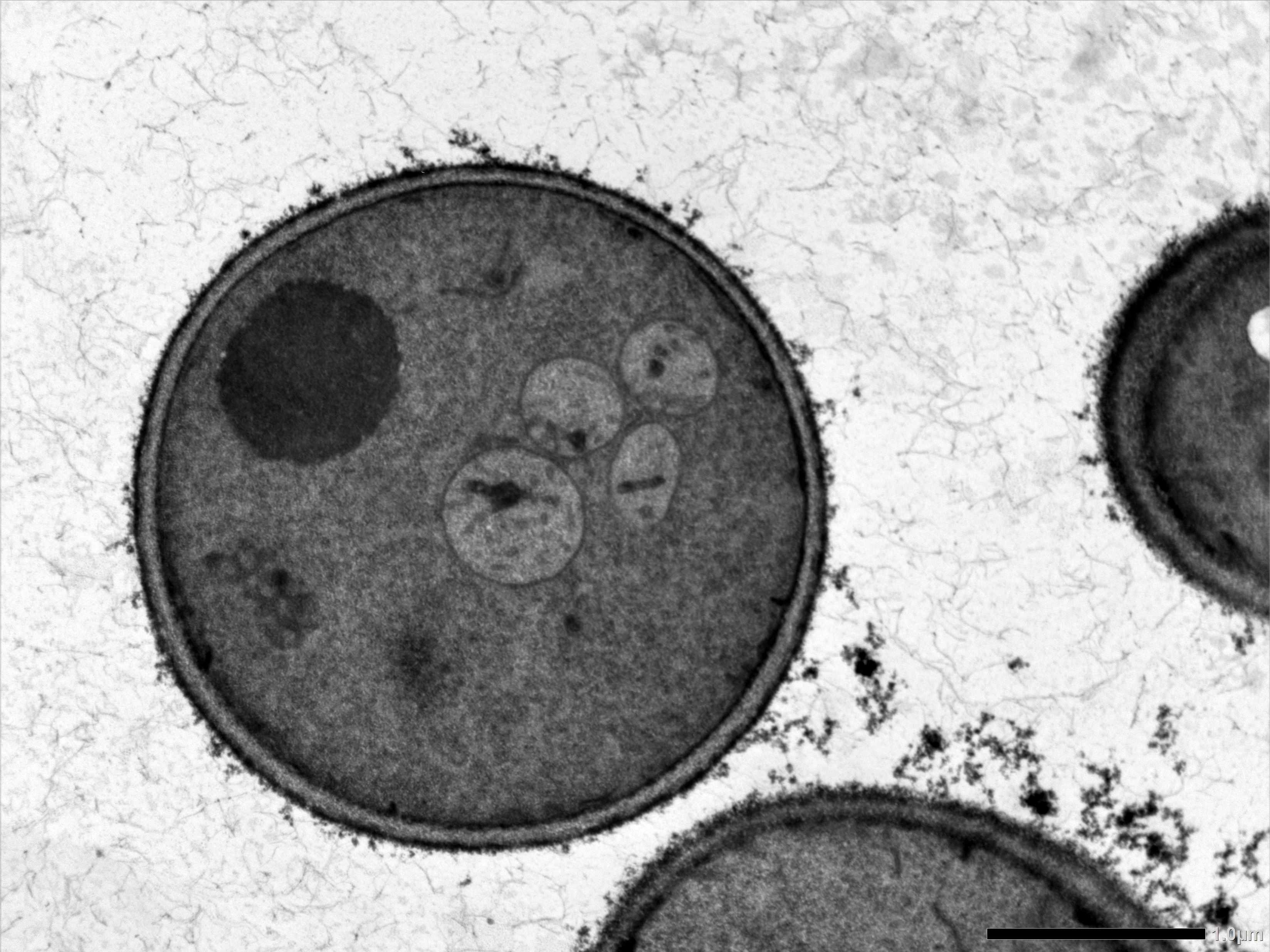

Supplement: Supplementary file 10 — Source data Fig. 2 [file 44319_2026_794_MOESM10_ESM.zip › Source Data Figure 2/2G_EM raw images/depletion t9_SA-MAG_X10k.jpg]

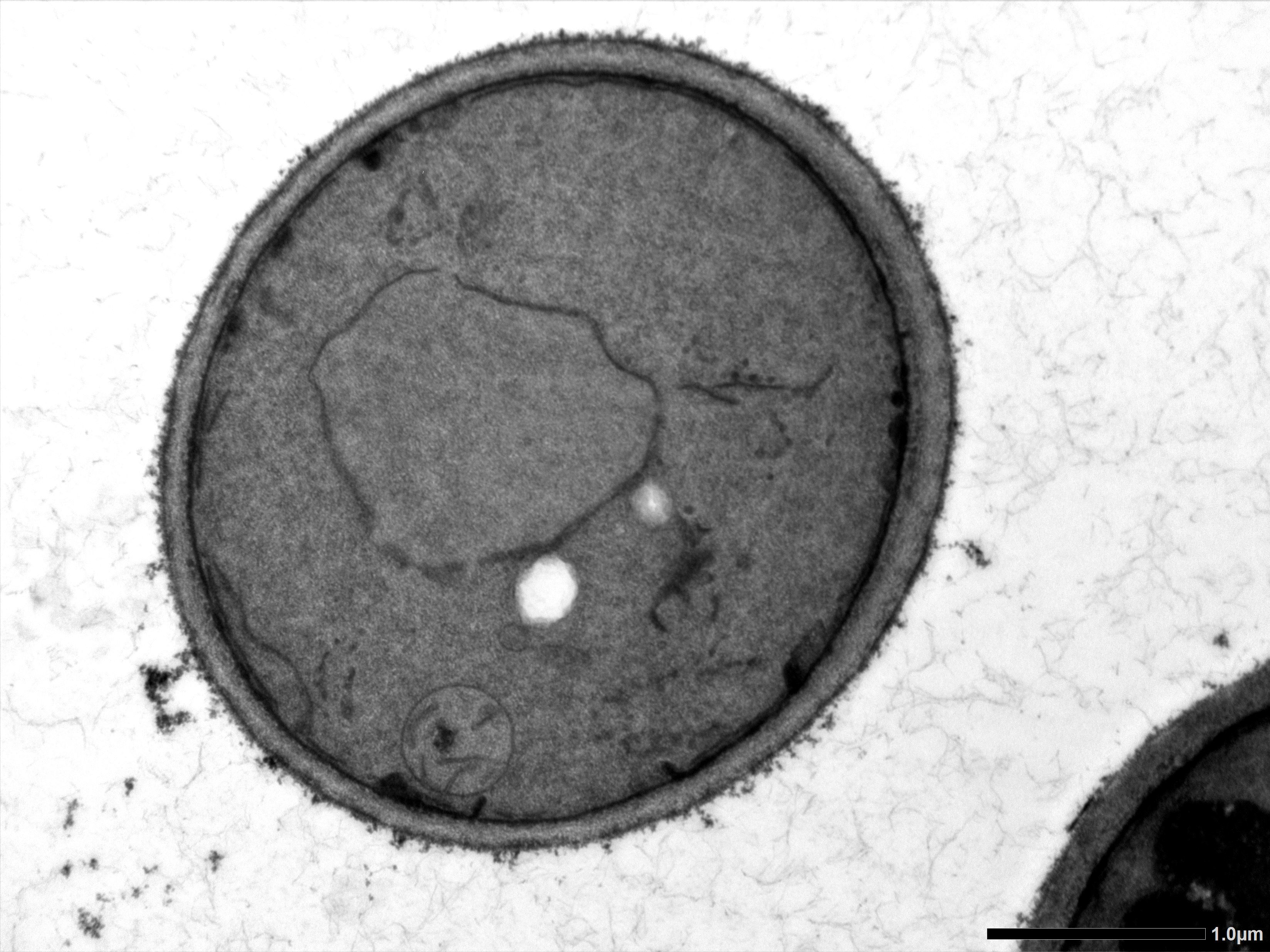

Supplement: Supplementary file 10 — Source data Fig. 2 [file 44319_2026_794_MOESM10_ESM.zip › Source Data Figure 2/2G_EM raw images/wildtype example_SA-MAG_X10k.jpg]
