# Supplementary material for: In the absence of mitochondrial fusion unequal segregation of mitochondria drives mtDNA loss
Source: EMBO Rep. 2026 May 14;27(12):3359–93. doi: 10.1038/s44319-026-00794-5 (PMC13303861; doi:10.1038/s44319-026-00794-5)
Supplement: Supplementary file 17 — Expanded View Figures [file 44319_2026_794_MOESM17_ESM.pdf]

Expanded View Figures

Figure EV1. Mitochondrial morphology, mtDNA levels, and growth are not affected by genetic constructs or the addition of chemicals.

Cells were grown in synthetic complete (SC) medium containing 1% glucose, and Fzo1 depletion was induced at the indicated times. (**A**) Solidity of the mitochondrial network from epifluorescence microscopy. Mean of three biological replicates with a total of 1694 Control and 1694 Fzo1-depleted cells. (**B**) Example images of maximum z-projections of mitochondria visualized by pre-Su9-mCardinal. Scale bar = 10 µm. 1: Control = AID-Fzo1, TIR, untreated; 2: TIR + anhydrotetracycline (aTC) + 5-Ph-IAA, no AID-Flag; 3: Fzo1-AID, TIR + aTC before addition of 5-Ph-IAA; 4: AID-Fzo1, TIR + aTC + 5-Ph-IAA (1 h 5-Ph-IAA = Fzo1 depletion); 5: Fzo1-AID, TIR + aTC + 5-Ph-IAA (21 h 5-Ph-IAA = Fzo1 depletion); 6: Δ*fzo1*. (**C**) Solidity of different control strains and treatments from three biological replicates. Whiskers indicate 10th and 90th percentiles, + indicates mean, line indicates median, boxes show 25th and 75th percentiles. Control (all 8.5 h of imaging) 26850 datapoints of 1694 cells; TIR + aTC + 5-Ph-IAA (after addition of the chemicals) 17170 datapoints of 1078 cells; Fzo1-AID, TIR, before depletion (0.5 h before addition of 5-Ph-IAA, aTC is added already) 207 datapoints of 57 cells; 18 h Fzo1 depleted 1434 datapoints of 1434 cells; Δ*fzo1* (all of 10 h imaging) 43414 datapoints of 1419 cells. (**D**) Cell cycle duration of WT cells, cells with TIR treated with aTC and 5-Ph-IAA, cells with FLAG-AID-Fzo1 treated with aTC and 5-Ph-IAA, and cells with TIR and FLAG-AID-Fzo1 without treatment. Mean with SD from three biological replicates is shown. (**E**) DNA-qPCR of WT, 2 h aTC-treated WT, TIR-expressing cells treated with aTC and 5-Ph-IAA for 3 h, and cells expressing AID-Fzo1 and TIR treated with aTC for 2 h. mtDNA per nuclear DNA values were normalized to 0 h WT samples. Mean with SD from three biological replicates is shown. (**F**) Unnormalized cell cycle durations corresponding to growth rates shown in Fig. 1D. Mean with SD from three biological replicates is shown. (**G**) Cell cycle durations from live-cell microscopy. Data from three biological replicates is shown. Control: $n = 1250$, 0–8 h Fzo1 depleted: $n = 1409$, 8–18 h Fzo1 depleted: $n = 1755$, Δ*fzo1*: $n = 1277$. Whiskers indicate 10th and 90th percentiles, + indicates mean, line indicates median, boxes show 25th and 75th percentiles. Cell cycle durations from live-cell microscopy are comparable to shake flask cell cycle durations.

 © The Author(s)

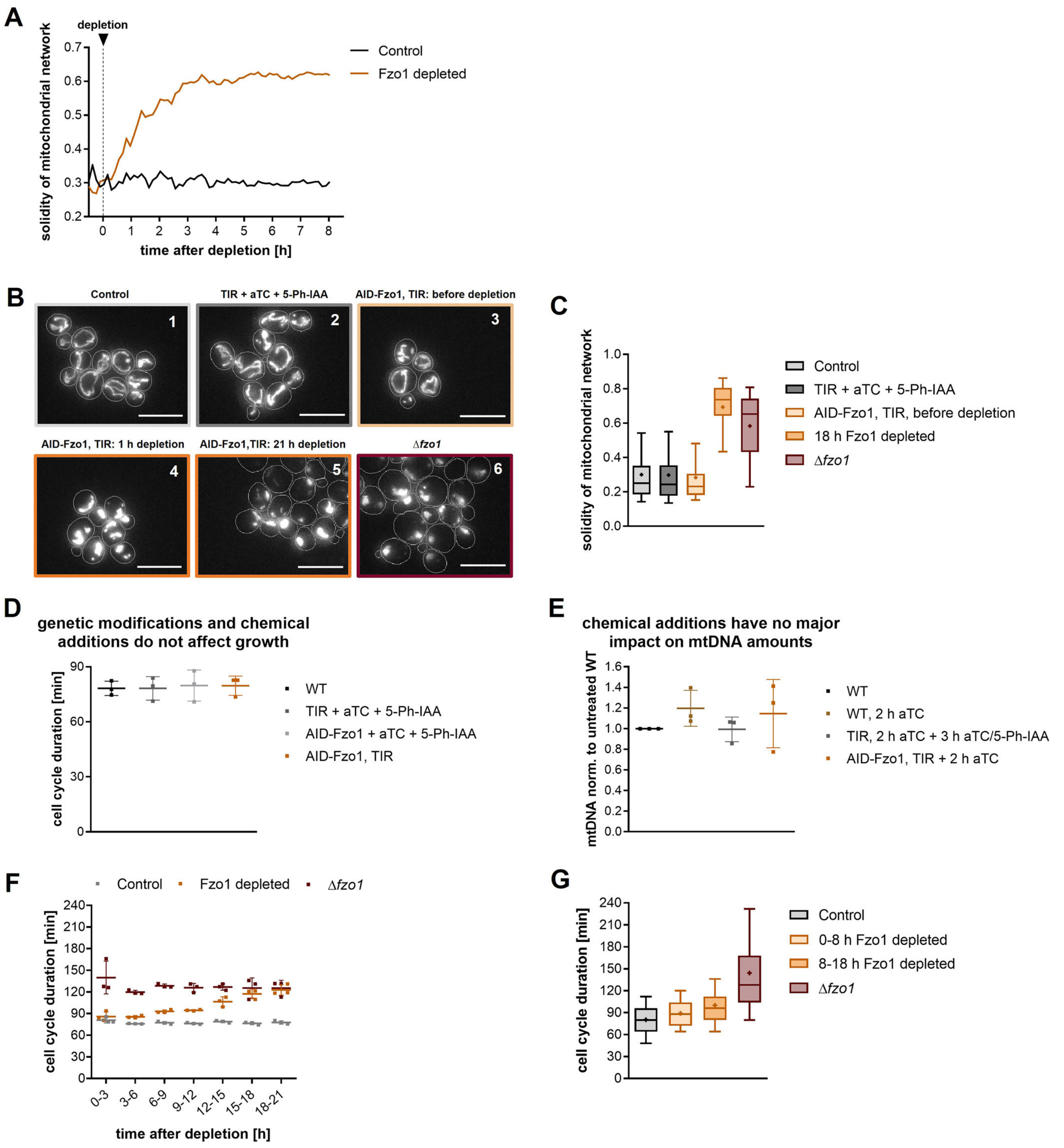
A
depletion
solidity of mitochondrial network
time after depletion [h]
Control
Fzo1 depleted
B
Control
TIR + aTC + 5-Ph-IAA
AID-Fzo1, TIR: before depletion
AID-Fzo1, TIR: 1 h depletion
AID-Fzo1,TIR: 21 h depletion
Δfzo1
C
solidity of mitochondrial network
Control
TIR + aTC + 5-Ph-IAA
AID-Fzo1, TIR, before depletion
18 h Fzo1 depleted
Δfzo1
D
genetic modifications and chemical additions do not affect growth
cell cycle duration [min]
WT
TIR + aTC + 5-Ph-IAA
AID-Fzo1 + aTC + 5-Ph-IAA
AID-Fzo1, TIR
E
chemical additions have no major impact on mtDNA amounts
mtDNA norm. to untreated WT
WT
WT, 2 h aTC
TIR, 2 h aTC + 3 h aTC/5-Ph-IAA
AID-Fzo1, TIR + 2 h aTC
F
Control
Fzo1 depleted
Δfzo1
cell cycle duration [min]
time after depletion [h]
G
cell cycle duration [min]
Control
0-8 h Fzo1 depleted
8-18 h Fzo1 depleted
Δfzo1

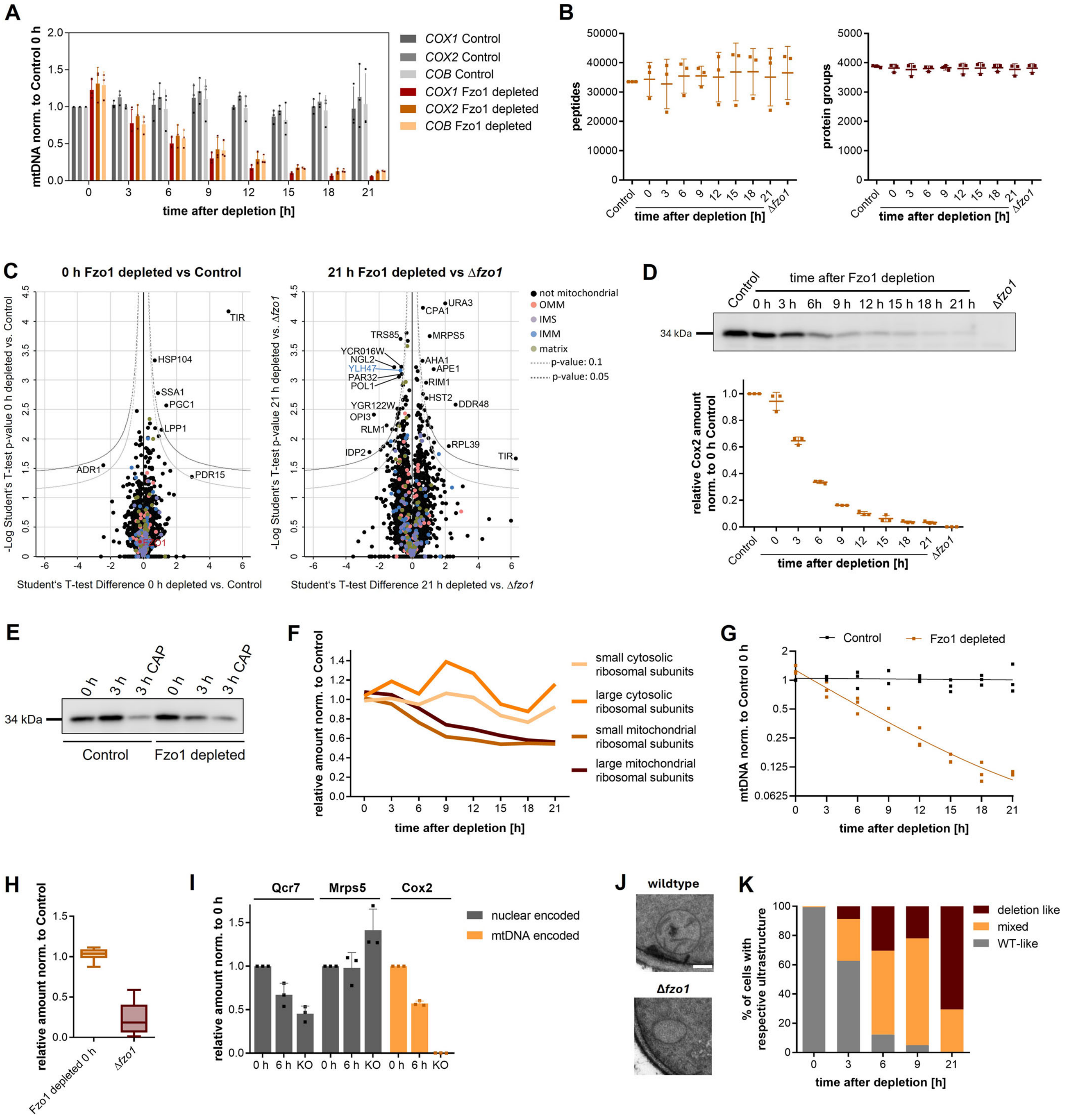
A
B
C
0 h Fzo1 depleted vs Control
21 h Fzo1 depleted vs Δfzo1
D
time after Fzo1 depletion
E
Control
Fzo1 depleted
F
G
H
I
J
wildtype
Δfzo1
K
time after depletion [h]

 © The Author(s)

Figure EV2. Progressive loss of mtDNA results in the full establishment of the Fzo1 deletion phenotype.

(**A**) DNA-qPCR of individual mitochondrial genes. The level of each gene was normalized to the amount of the nuclear gene *ACT1*. Mean and SD from three biological replicates are shown. (**B**) The number of peptides and protein groups detected in the mass spectrometry analysis. Mean with SD from three biological replicates is shown. (**C**) Volcano plots of proteomics measurement of 0 h depleted vs Control cells (left) and 21 h depleted vs Δ*fzo1* cells. 0 h Fzo1 depleted (treated with aTC for 2 h) shows a comparable proteome as Control cells (no aTC). As expected, the strongest and most significant difference is the TIR protein. Data from three biological replicates is shown. (**D**) Example Western blot and quantification of Cox2 levels by. Mean and SD from three biological replicates. (**E**) Example Western blot of Chloramphenicol (CAP) treatment quantified in Fig. 2C. (**F**) Abundance of mitochondrial ribosomal subunits and cytosolic ribosomal subunits measured by mass spectrometry-based proteomics in three replicates, normalized to $t = 0$. The average of all measured protein groups of the respective subunits is shown. (**G**) mtDNA levels shown in Fig. 2A from three biological replicates on log2 scale. Solid lines show a fit to a one-phase exponential decay with a half-life of 4.7 h for Fzo1-depleted cells. (**H**) Abundance of 30 nuclear encoded respiratory chain proteins of Control and Δ*fzo1* cells (related to Fig. 2D) as determined by mass spectrometry from three biological replicates. Whiskers indicate 10th and 90th percentiles, + indicates mean, line indicates median, boxes show 25th and 75th percentiles. (**I**) Expression of nuclear encoded Qcr7 and Mrps5, and mtDNA encoded Cox2 determined by RT-qPCR. Mean and SD from three biological replicates with three technical replicates each are shown. (**J**) Example pictures of electron microscopy related to Fig. 2H), scale bar = 200 nm. (**K**) Categorization of cells based on their ultrastructure in electron microscopy of three biological replicates, related to Fig. 2H.

© The Author(s)

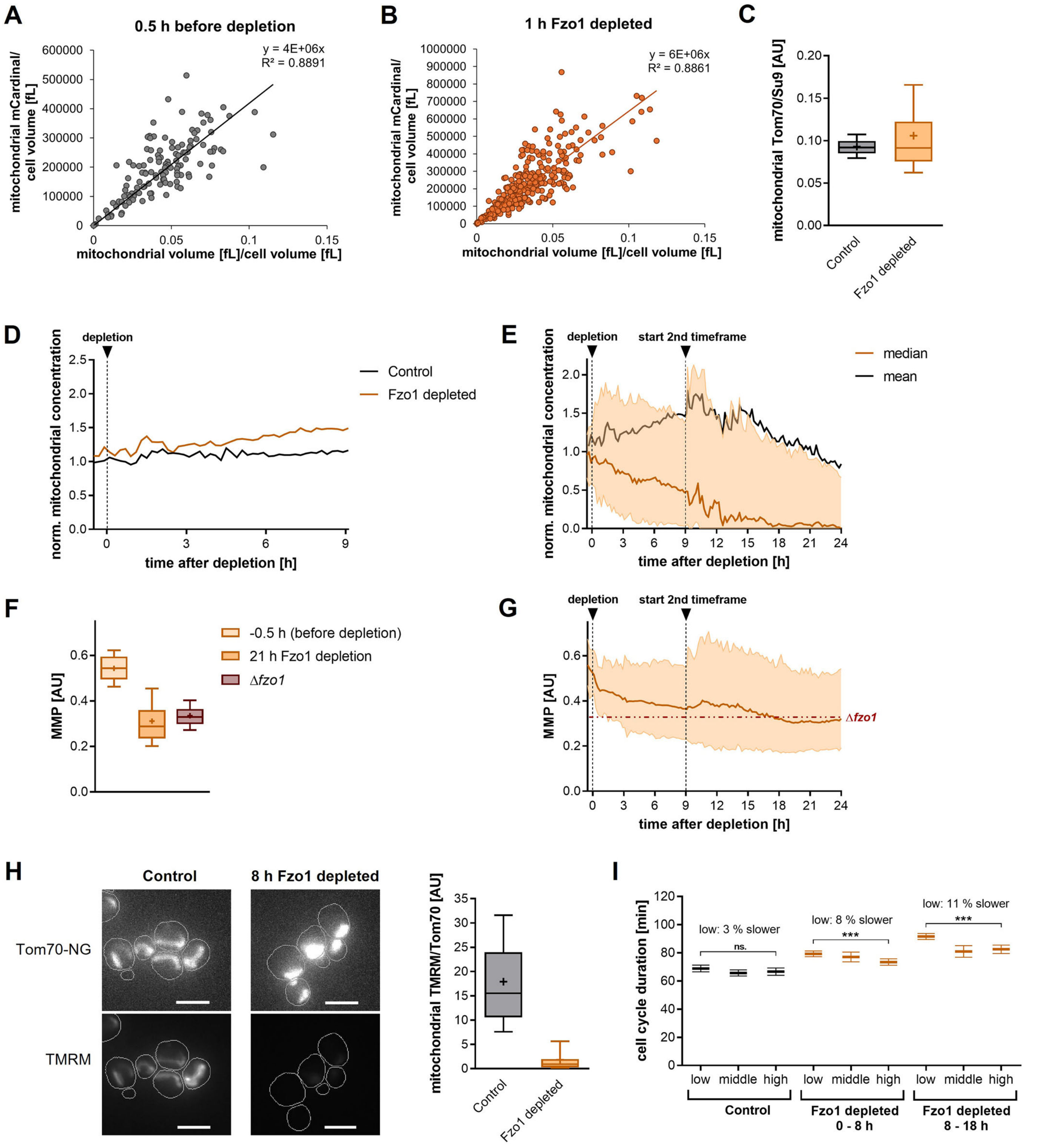
A
0.5 h before depletion
y = 4E+06x
R² = 0.8891
mitochondrial mCardinal/ cell volume [fL]
mitochondrial volume [fL]/cell volume [fL]
B
1 h Fzo1 depleted
y = 6E+06x
R² = 0.8861
mitochondrial mCardinal/ cell volume [fL]
mitochondrial volume [fL]/cell volume [fL]
C
mitochondrial Tom70/Su9 [AU]
Control
Fzo1 depleted
D
depletion
Control
Fzo1 depleted
norm. mitochondrial concentration
time after depletion [h]
E
depletion
start 2nd timeframe
median
mean
norm. mitochondrial concentration
time after depletion [h]
F
-0.5 h (before depletion)
21 h Fzo1 depletion
Δfzo1
MMP [AU]
G
depletion
start 2nd timeframe
Δfzo1
MMP [AU]
time after depletion [h]
H
Control
8 h Fzo1 depleted
Tom70-NG
TMRM
mitochondrial TMRM/Tom70 [AU]
Control
Fzo1 depleted
I
low: 3 % slower
ns.
low: 8 % slower
***
low: 11 % slower
***
cell cycle duration [min]
low middle high
Control
Fzo1 depleted 0 - 8 h
Fzo1 depleted 8 - 18 h

 © The Author(s)

Figure EV3. Quantification of mitochondrial concentration and mitochondrial membrane potential by microscopy.

(**A**, **B**) Mitochondrial volume estimated from a 3D volume reconstruction was compared to mitochondrial volume estimated from total pre-Su9-mCardinal fluorescence intensity. mCardinal signal correlates with mitochondrial volume/cell volume. However, due to the morphology change the slope of the correlation changes, likely to bias in the 3D reconstruction. Therefore, we consider the mitochondrial mCardinal as the better proxy for mitochondrial concentration. (**A**) mitochondrial volume [fL]/ cell volume [fL] vs mitochondrial mCardinal/cell volume [fL] before depletion. $n = 129$. (**B**) mitochondrial volume [fL]/cell volume [fL] vs mitochondrial mCardinal/cell volume [fL] 1 h after Fzo1 depletion. $N = 286$. (**C**) Mitochondrial Tom70-Neongreen/preSu9-mCardinal for Control and Fzo1-depleted cells 8 h after depletion. n (Control) = 127, *n* (Fzo1 depletion) = 165. Whiskers indicate 10th and 90th percentiles, + indicates mean, line indicates median, boxes show 25th and 75th percentiles. (**D**) Mean of the normalized mitochondrial concentrations after Fzo1 depletion. Same data as in Fig. 3A. (**E**) Normalized mitochondrial concentrations after Fzo1 depletion. Shaded areas show 25th and 75th percentiles. Cells were imaged in two time intervals: −0.5–9 h and 9–24 h after Fzo1 depletion. *n* (24 h Fzo1 depletion) = 2019. (**F**) MMP of controls as described in Fig. 3. −0.5 h before depletion: 184 datapoints of 67 cells, 21 h Fzo1 depletion: 718 datapoints of 718 cells, Δ*fzo1:* 25,197 datapoints of 1376 cells over 10 h of imaging. Whiskers indicate 10th and 90th percentiles, + indicates mean, line indicates median, boxes show 25th and 75th percentiles. (**G**) MMP of Fzo1-depleted cells shown in (**E**). Shaded areas show 5th and 95th percentiles. *n* (24 h Fzo1 depletion) = 2019. The dashed line depicts the median MMP of Δ*fzo1* cells. (**H**) TMRM staining of Control and 8 h Fzo1-depleted cells. Left: example images of maximum z-projections, scale bar = 5 μm. Right: Quantification of the TMRM staining: mitochondria were segmented using Tom70-Neongreen, and the TMRM signal was normalized to the Tom70 signal. *n* (Control) = 290, *n* (Fzo1 depletion) = 189. Whiskers indicate 10th and 90th percentiles, + indicates mean, line indicates median, boxes show 25th and 75th percentiles. (**I**) Cell cycle duration of generation >1 with different mitochondrial concentrations. Categorization was performed based on Control cells gen >1. Images were taken every 8 min to determine cell cycle durations. Mean with 95% Confidence interval is shown. Statistical significance was determined using a paired two-tailed *t* test. Control low vs high: $P = 0.219$, 0–8 h Fzo1 depleted: $P = 0.0005$, 8–18 h Fzo1 depleted: $P < 0.0001$. Control: *n* (low) = 233, *n* (middle) = 233, *n* (high) = 240; Fzo1 depleted 0–8 h: *n* (low) = 460, *n* (middle) = 98, *n* (high) = 232; Fzo1 depleted 8–18 h: *n* (low) = 714, *n* (middle) = 82, *n* (high) = 232.

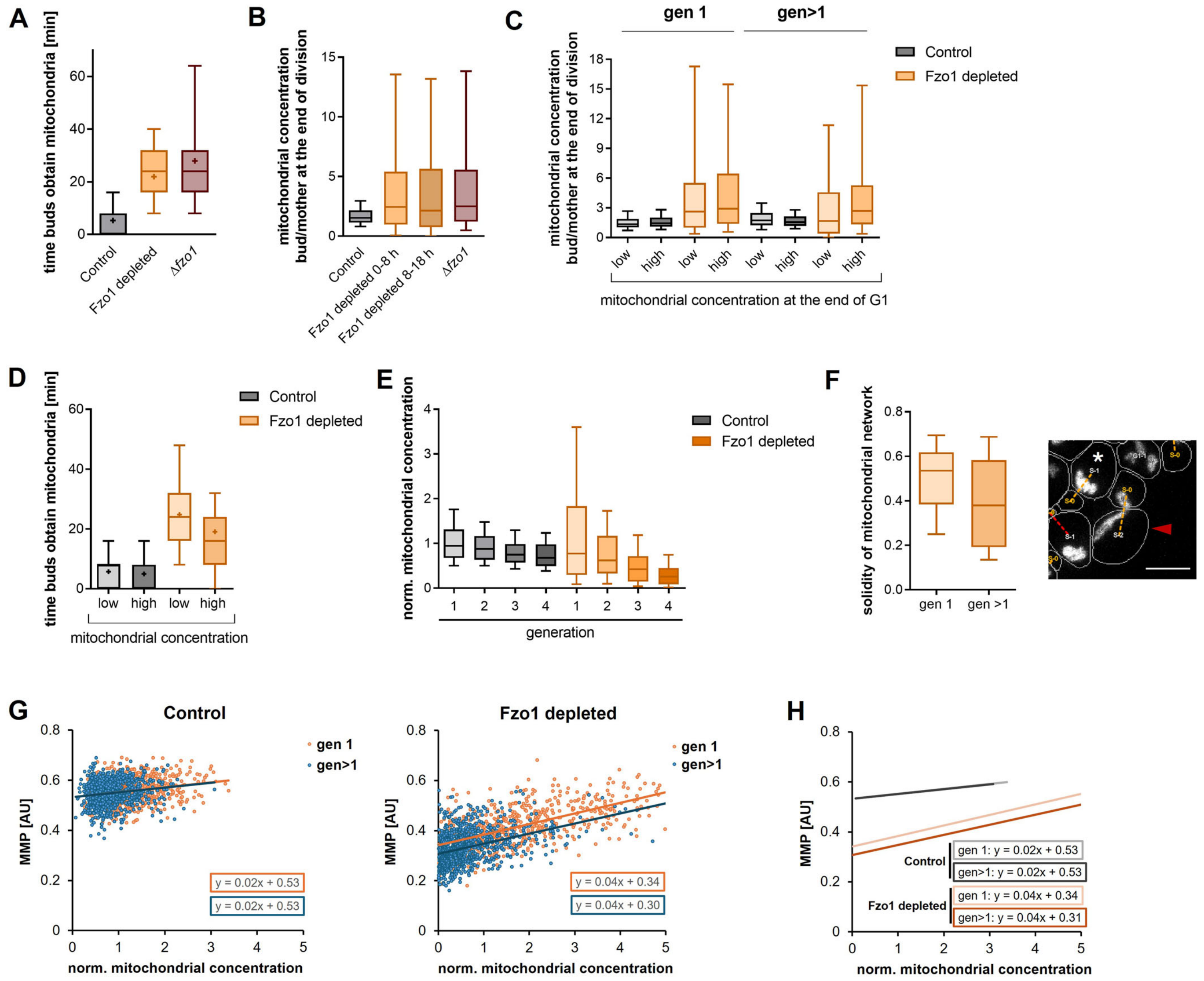

Figure EV4. First-generation cells with higher mitochondrial concentrations exhibit a higher mitochondrial membrane potential and earlier mitochondrial inheritance.

(**A**) Time when mitochondria are first detected in the bud. Control = 1249, Fzo1 depleted = 1275, Δ*fzo1* = 1237. (**B**) Ratio of the mitochondrial concentration of buds to mothers at the end of the cell cycle. *n* (Control) = 1250, *n* (Fzo1 depleted 0–8 h) = 1324, *n* (Fzo1 depleted 8–18 h) = 1410, *n* (Δ*fzo1*) = 1226. (**C**) Ratio of the mitochondrial concentration of buds to mothers at the end of the cell cycle, sorted by the mitochondrial concentration at the end of G1. Grouping is based on the quartiles of the Control. gen 1 (Control): *n* (low) = 272, *n* (high) = 272; gen 1 (Fzo1 depleted): *n* (low) = 164, *n* (high) = 425; gen > 1 (Control): *n* (low) = 353, *n* (high) = 353; gen > 1 (Fzo1 depleted): *n* (low) = 473, *n* (high) = 262. (**D**) The time when mitochondria are first detected in the bud of mothers with a low or high mitochondrial concentration. The median mitochondrial concentration of Control cells at G1 was used to determine low and high categories. Control: *n* (low) = 625, *n* (high) = 624; Fzo1 depleted: *n* (low) = 638, *n* (high) = 637. (**E**) Mitochondrial concentrations of cells shown in Fig. 4I. Distributions differ significantly across generations under a Gamma likelihood-ratio test ($P < 2.225 \times 10^{-308}$). Medians decrease strongly and significantly for Control and Fzo1-depleted cells from the first to fourth generation (Wilcoxon rank-sum tests, $P = 8.456 \times 10^{-11}$ for Control and $P = 3.231 \times 10^{-20}$ for Fzo1-depleted cells) with the median in the first generation being 1.4-fold and 3.1-fold higher than the fourth generation in Control and Fzo1-depleted cells, respectively. (**F**) Solidity of gen 1 and gen >1 mothers throughout their cell cycle is shown. *n* > 11,000 datapoints of at least 257 cells each. Red arrow on the example image shows a gen > 1 mother, white asterisk shows a first-generation mother. Scale bar = 5 µm. The difference in mitochondrial morphology is likely one factor that contributes to the lower inheritance of mitochondria to buds in higher generations. In higher generation cell cycles the mitochondrial network of the mothers more likely exhibits strings as observed by a lower solidity than in first-generation divisions. Of note, these are only transient morphological changes and are not a sign of incomplete Fzo1 depletion in these mitochondria. (**G**) MMP vs mitochondrial concentration of mothers of gen 1 or gen > 1 for Control and Fzo1-depleted cells (0–9 h following Fzo1 depletion). Control: *n* (gen 1) = 687, *n* (gen >1) = 943. Fzo1 depleted: *n* (gen 1) = 745, *n* (gen >1) = 1024. (**H**) Slopes of the data shown in (**G**). (**A–F**) Whiskers of box plots indicate 10th and 90th percentiles, line indicates median, boxes show 25th and 75th percentiles. Where shown, + indicate means.

 © The Author(s)

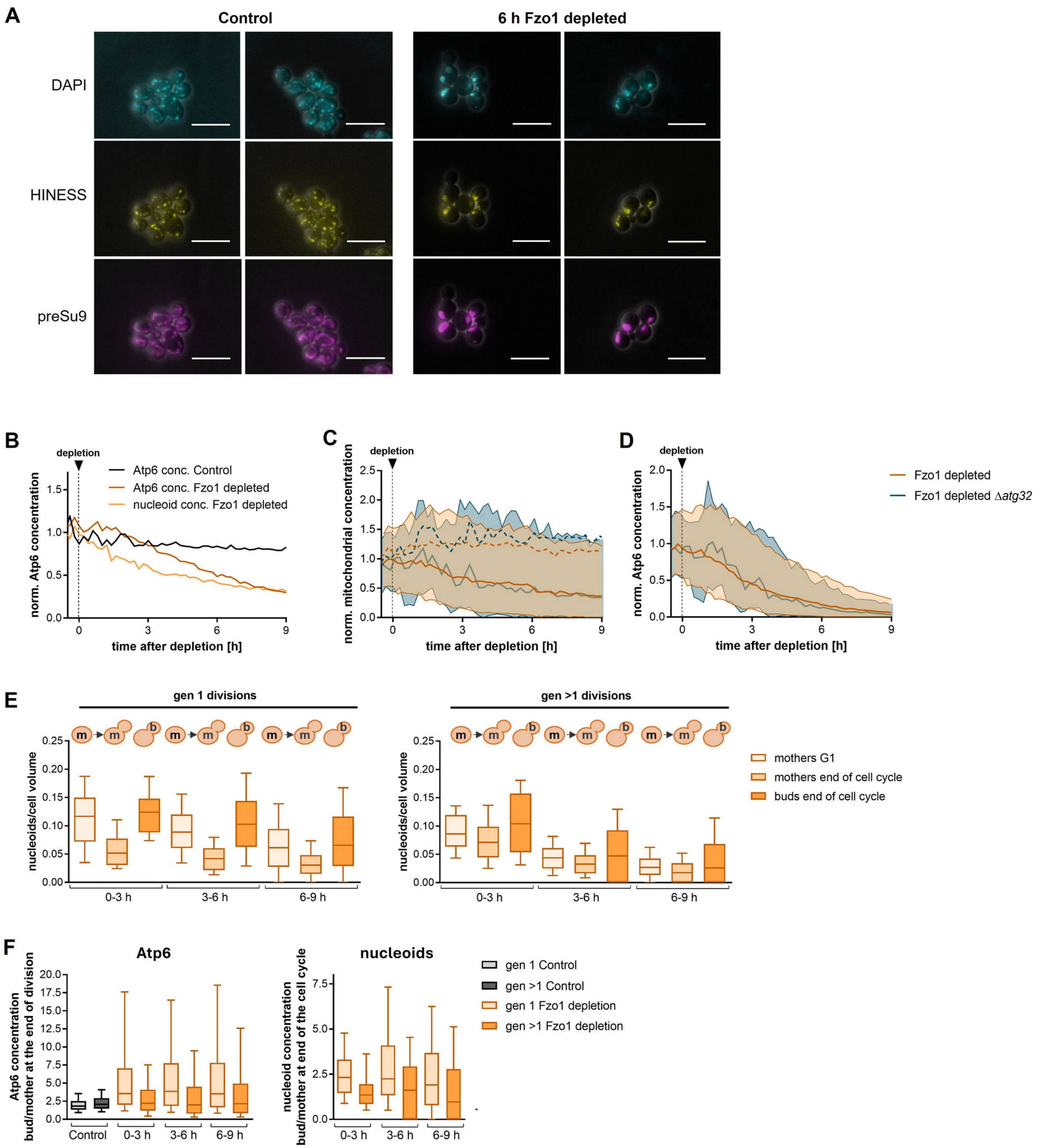
A
Control
6 h Fzo1 depleted
DAPI
HINESS
preSu9
B
depletion
Atp6 conc. Control
Atp6 conc. Fzo1 depleted
nucleoid conc. Fzo1 depleted
norm. Atp6 concentration
time after depletion [h]
C
depletion
norm. mitochondrial concentration
time after depletion [h]
D
depletion
norm. Atp6 concentration
time after depletion [h]
Fzo1 depleted
Fzo1 depleted Δatg32
E
gen 1 divisions
gen >1 divisions
nucleoids/cell volume
0-3 h
3-6 h
6-9 h
mothers G1
mothers end of cell cycle
buds end of cell cycle
F
Atp6
nucleoids
Atp6 concentration bud/mother at the end of division
nucleoid concentration bud/mother at end of the cell cycle
Control
0-3 h
3-6 h
6-9 h
gen 1 Control
gen >1 Control
gen 1 Fzo1 depletion
gen >1 Fzo1 depletion

Figure EV5. Atp6 concentration is a good proxy for nucleoid counts.

(**A**) Example images of DAPI staining compared to mt-Kaede-HI-NESS of Control and Fzo1-depleted cells 6 h after depletion. Scale bar = 10 µm. (**B**) Atp6 and nucleoid concentration after Fzo1 depletion. Same data as in main Fig. 5A,B. (**C**) Mitochondrial concentration after Fzo1 depletion in Δ*atg32* cells which are unable to perform mitophagy. Mean (dashed lines) and median (solid lines) with 25th and 75th percentiles are shown for Fzo1-depleted cells without additional mutation (orange) and for Fzo1-depleted cells with Atg32 deletion (turquoise). *n* (Fzo1 depletion at 9 h) = 3686 cells from three biological replicates, *n* (Fzo1 depletion with Δ*atg32* at 9 h) = 1909 cells from two biological replicates. (**D**) Atp6-Neongreen concentration of data shown in (**C**). (**E**) Nucleoid concentrations of mothers at the beginning of the cell cycle (G1), and of mothers and buds at the end of the cell cycle. *n* (gen 1): 0–3 h = 48, 3–6 h = 225, 6–9 h = 848; *n* (gen >1): 0–3 h = 71, 3–6 h = 303, 6–9 h = 1111. Whiskers indicate 10th and 90th percentiles, line indicates median, boxes show 25th and 75th percentiles. (**F**) Ratio of the Atp6 and nucleoid concentrations between buds and mothers at the end of division. Atp6 (corresponding to data shown in Fig. 5C) *n* (gen 1): Control = 1095, 0–3 h = 569, 3–6 h = 2122, 6–9 h = 728; *n* (gen >1): Control = 1389, 0–3 h = 777, 3–6 h = 2293, 6–9 h = 789. Nucleoid concentration (corresponding to data shown in (**E**) *n* (gen 1): 0–3 h = 47, 3–6 h = 210, 6–9 h = 682; *n* (gen >1): 0–3 h = 70, 3–6 h = 274, 6–9 h = 801. Whiskers indicate 10th and 90th percentiles, line indicates median, boxes show 25th and 75th percentiles.

 © The Author(s)

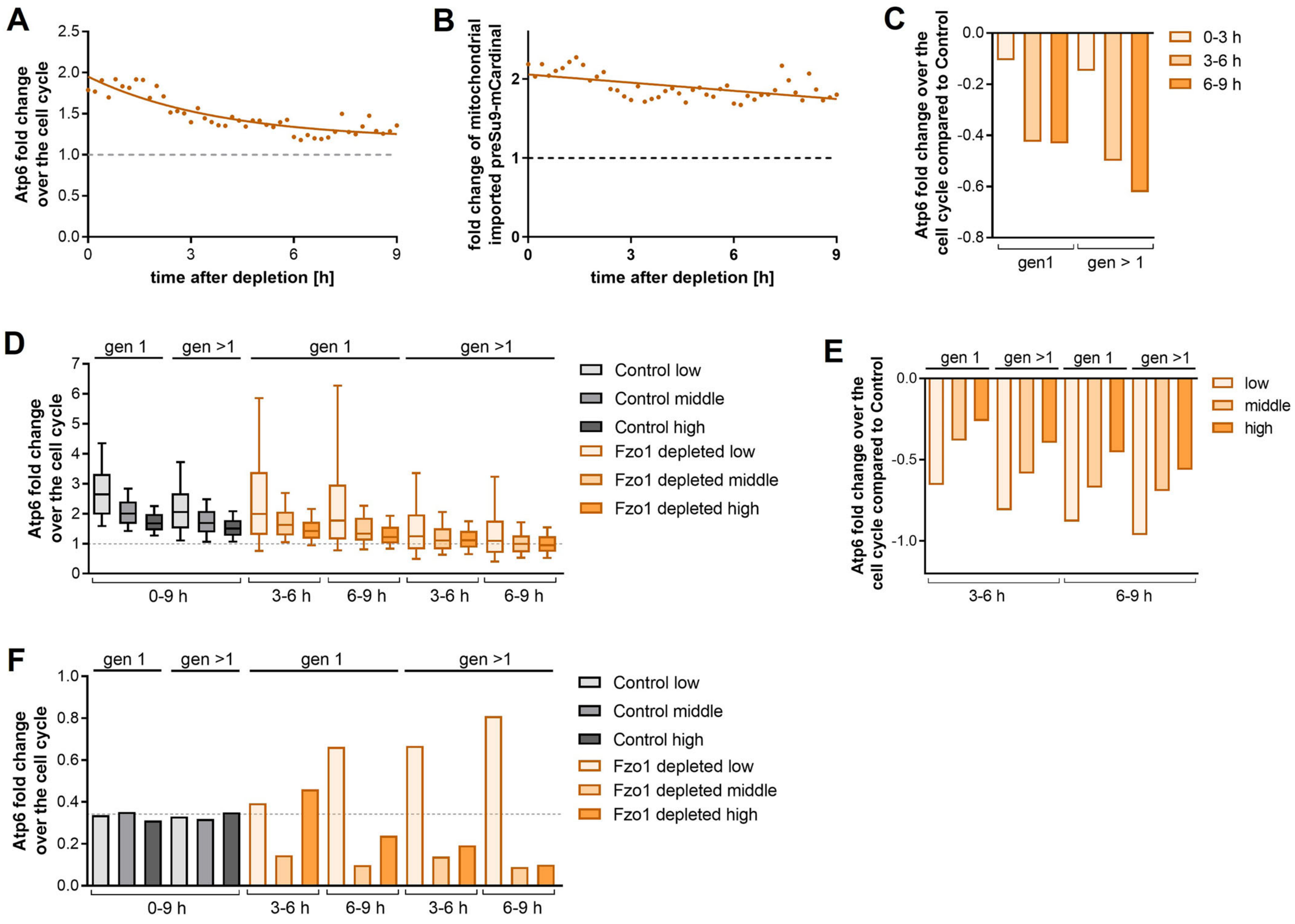

Figure EV6. Synthesis of mtDNA-encoded proteins is most strongly reduced in higher generations.

(**A**) Fold change of Atp6 per cell cycle of all generations after Fzo1 depletion, related to Fig. 6B. In all, 9013 cell divisions were analyzed. (**B**) Fold change of mitochondrial imported preSu9-mCardinal per cell cycle of all generations after Fzo1 depletion as in (**A**). (**C**) Difference of the median fold change of Atp6 over the cell cycle in Fzo1-depleted versus Control cells of gen 1 and gen >1 divisions, related to Fig. 6C. (**D**) Fold change of Atp6 over the cell cycle of first and higher generation divisions of mothers with low, middle, or high Atp6 content at the beginning of the cell cycle. At least 79 cell cycles were analyzed for each group. Whiskers indicate 10th and 90th percentiles, line indicates median, boxes show 25th and 75th percentiles. (**E**) Difference of the median Atp6 fold change of Fzo1-depleted cells compared to the respective Control group of cells shown in (**D**). (**F**) Fraction of the population of cells shown in (**D**).

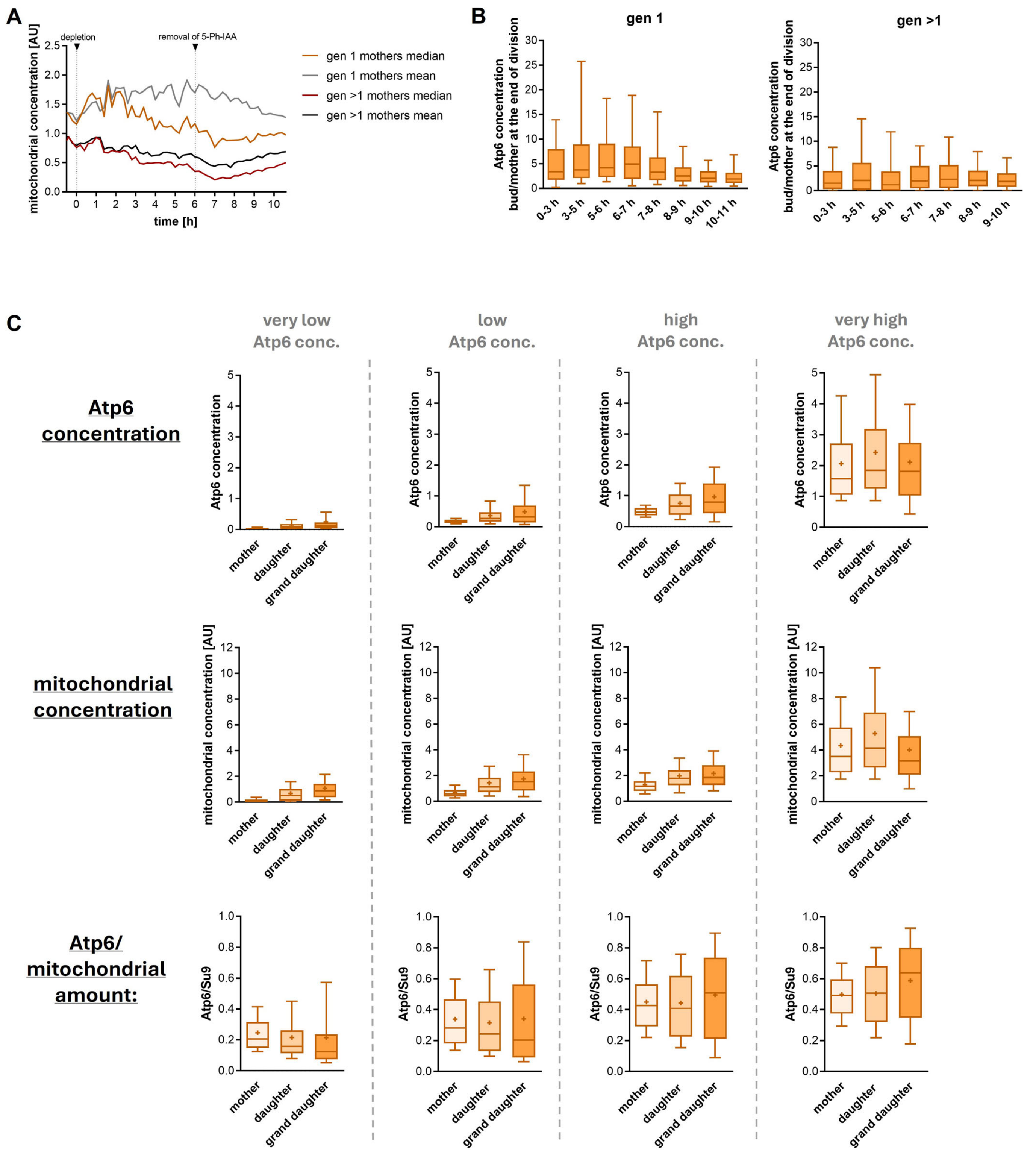
A
depletion
removal of 5-Ph-IAA
mitochondrial concentration [AU]
time [h]
gen 1 mothers median
gen 1 mothers mean
gen >1 mothers median
gen >1 mothers mean
B
gen 1
gen >1
Atp6 concentration bud/mother at the end of division
C
very low Atp6 conc.
low Atp6 conc.
high Atp6 conc.
very high Atp6 conc.
Atp6 concentration
mitochondrial concentration
Atp6/ mitochondrial amount:
Atp6/Su9
mother
daughter
grand daughter

 © The Author(s)

Figure EV7. Cells with low amounts of mtDNA-encoded proteins are unable to recover after Fzo1 re-addition.

(**A**) Mean and median mitochondrial concentration of first and higher generation mothers. 176 mothers at 10.6 h for gen 1 and 210 for gen >1. (**B**) Bud/mother Atp6 concentration ratios at the end of division as shown for the mitochondrial concentration in Fig. 7C. Whiskers indicate 10th and 90th percentiles, line indicates median, boxes show 25th and 75th percentiles. (**C**) Atp6 concentrations of mothers, daughters, and granddaughters after re-addition of Fzo1. Selection is based on daughters that were born at least 1 h after removal of 5-Ph-IAA. Grouping is based on the Atp6 concentration of mothers, which were divided into four equal groups. Each group contains 163 mother–daughter–granddaughter lineages. Whiskers indicate 10th and 90th percentiles, + indicates mean, line indicates median, boxes show 25th and 75th percentiles.

© The Author(s)

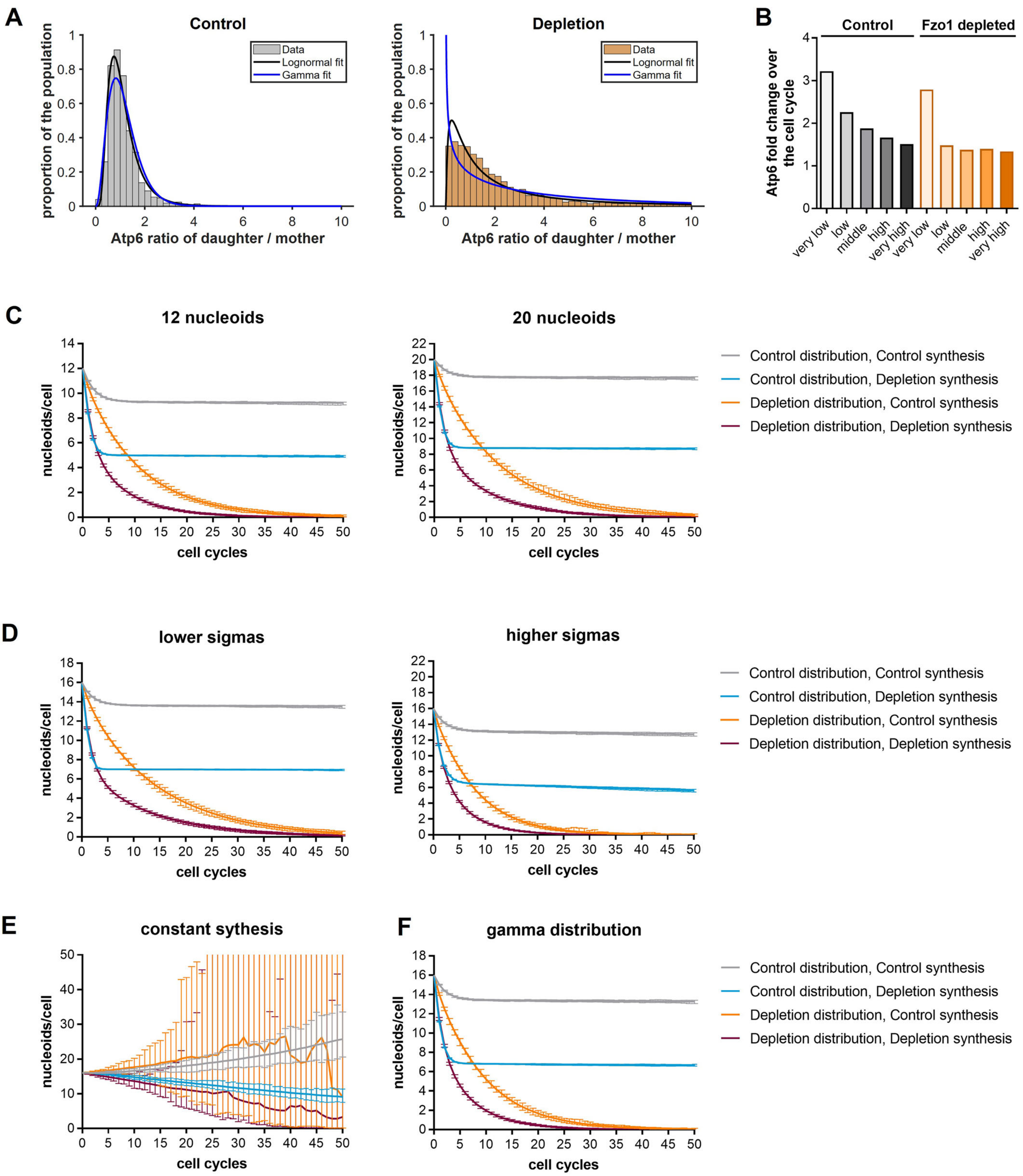
A
Control
Depletion
Data
Lognormal fit
Gamma fit
proportion of the population
Atp6 ratio of daughter / mother
B
Control
Fzo1 depleted
Atp6 fold change over the cell cycle
very low
low
middle
high
very high
C
12 nucleoids
20 nucleoids
nucleoids/cell
cell cycles
Control distribution, Control synthesis
Control distribution, Depletion synthesis
Depletion distribution, Control synthesis
Depletion distribution, Depletion synthesis
D
lower sigmas
higher sigmas
E
constant sythesis
F
gamma distribution

 © The Author(s)

Figure EV8. Sensitivity analysis of model parameters.

(**A**) Histogram with lognormal (black line) and gamma (blue line) distribution fits of the ratio of Atp6 between daughters and their mothers immediately after division is completed. (**B**) Average fold change of Atp6 over the cell cycle of Control and Fzo1-depleted cells with different Atp6 content of the mother at the beginning of the cell cycle. mtDNA synthesis was determined based on the Atp6 content at the beginning of G1. (**C–E**) Nucleoid levels modeled with Atp6 daughter/mother ratios fitted with lognormal distributions. Mean and range of 100 simulations are shown. (**C**) Nucleoid levels modeled with lower (12 nucleoids) or higher (20 nucleoids) starting amounts compared to 16 nucleoids used for the simulation in Fig. 8. (**D**) Nucleoid levels modeled with lower and higher sigmas compared to the sigmas obtained from the measured data. (**E**) Nucleoid levels modeled with a constant synthesis rate instead of the category-dependent synthesis rates shown in (**B**). (**F**) Nucleoid levels modeled with a gamma distribution instead of the lognormal distribution. See "Methods" for further details.
