# Supplementary material for: In the absence of mitochondrial fusion unequal segregation of mitochondria drives mtDNA loss
Source: EMBO Rep. 2026 May 14;27(12):3359–93. doi: 10.1038/s44319-026-00794-5 (PMC13303861; doi:10.1038/s44319-026-00794-5)
Supplement: Supplementary file 2 — Peer Review File [file 44319_2026_794_MOESM2_ESM.pdf]

EMBO reports

In the absence of mitochondrial fusion unequal segregation of mitochondria drives mtDNA loss

Lisa Dengler, Francesco Padovani, Bianca Lemke, Rebecca Brugger, Alissa Benedikt, Benedikt Westermann, Boris Maček, Kurt Schmoller, and Jennifer Ewald

Corresponding author(s): Jennifer Ewald (jennifer-christina.ewald@uni-tuebingen.de)

Review Timeline:

Transfer Date:	9th Oct 25
Editorial Decision:	17th Oct 25
Revision Received:	13th Feb 26
Editorial Decision:	26th Mar 26
Revision Received:	3rd Apr 26
Accepted:	17th Apr 26

Editor: Martina Rembold

Transaction Report: This manuscript was transferred to EMBO Reports following peer review at Review Commons.

(Note: With the exception of the correction of typographical or spelling errors that could be a source of ambiguity, letters and reports are not edited. Depending on transfer agreements, referee reports obtained elsewhere may or may not be included in this compilation. Referee reports are anonymous unless the Referee chooses to sign their reports.)

Review #1

1. Evidence, reproducibility and clarity:

Evidence, reproducibility and clarity (Required)

This manuscript by Dengler et al examines the mechanisms underlying the mtDNA depletion observed in cells where mitochondrial fusion is disrupted by depletion of the fusion factor Fzo1. In Saccharomyces cerevisiae, the authors deplete Fzo1 and use live-cell imaging of thousands of cells to follow the effects and their dynamic following Fzo1 depletion. They find that Fzo1-depleted cells show very rapid mitochondrial fragmentation (within 1h of Fzo1 depletion), and also an immediate drop in mitochondrial membrane potential (MMP). MtDNA is lost by 15h, and along with it the expression of mitochondrially-encoded proteins. Nuclear-encoded mitochondrial proteins are also decreased though somewhat later, and the authors find that this is largely due to their degradation (probably a consequence of lack of mitochondrial import into low-MMP cells). Most importantly, the study identifies two separate mechanisms that together contribute to the loss of mt-encoded proteins in Fzo1-depleted cells: unequal distribution of mitochondria during cell division and the reduction of a fusion-dependent compensatory synthesis of mt-encoded proteins. Unexpectedly, Fzo1-depleted cells end up passing an increased (rather than decreased) amount of mitochondria and mitochondria-encoded proteins to their daughters. Over several generations, and combined with the loss of the compensatory synthesis of more mt-encoded proteins, this leads to the progressive loss of mtDNA and mtDNA-encoded proteins in the population.

The study is very thorough and the experiments contain the appropriate controls. The conclusions are convincing and largely supported by the experimental data that has been appropriately replicated. The data presentation is generally clear although the text could benefit from some streamlining.

However, addressing the following major comments is required:

1. Statistical comparisons are missing throughout the manuscript (with the exception of Fig. 2c). Appropriate statistical tests, along with p-values, should be used and reported where different gorups are compared, for example (but not limited to) Fig. 3d and most panels of Fig. 4.
2. I do not agree with the use of Atp6 protein as a direct read-out of mtDNA content. While Atp6 protein levels will decrease with decreasing mtDNA content, the inverse is not necessarily true: decreased Atp6 protein levels do not necessarily indicate decreased

mtDNA levels, because they could alternatively or additionally be caused by decreased transcription and/or translation. Therefore, please do not equate Atp6 protein levels to mtDNA levels, and instead rephrase the text referencing the Atp6 experiments in the Results and Discussion sections to measure "mtDNA expression" or "mt-encoded protein" or similar. For example, on p. 14 line 431 should read "mtDNA expression" rather than "decreased synthesis of mtDNA", and line 440 on the same page "mean mtDNA levels" should be "mtDNA expression" or similar.

3. In Fig. 3, the authors use the fluorescence intensity of a mitochondrially-targeted mCardinal as a read-out of mitochondrial mass. Please provide evidence that this is not affected by MMP, either with relevant references or by control experiments (e.g. comparing it to N-acridine orange or other MMP-independent dyes or methods).

4. In Fig. 2e-f, the authors use a promoter reporter with Neongreen to answer whether the reduced levels of the nuclear-encoded mitochondrial proteins Mrps5 and Qcr7 are due to decreased expression or to protein degradation, and find no evidence of degradation of the Neongreen reporter protein. However, subcellular localization might affect the availability of the protein to proteases. Although not absolutely required, it would be relevant to know if the Neongreen fusion protein is found in the same subcellular compartment as Mrps5 and Qcr7 at 0h and 9h after Fzo1 depletion.

5. Fzo1 depletion leads to a very rapid drop in MMP during the first hour of depletion. In the Discussion, can the authors speculate on the possible mechanism of this rapid MMP drop that occurs well before mtDNA or mt-encoded proteins are decreased in level?

6. In Fig. 2a, the mtDNA copy number of Fzo1-depleted cells is ca 1.3-fold of the control cells at the 0h timepoint. Why might this be? Is it an impact of one of the inducers? If so, we might be looking at the combination of two different processes when measuring copy number: one that is an induction caused by the inducer(s), and the other a consequence of Fzo1 depletion itself.

Minor comments:

- p. 3, line 71: "ten thousands of dividing cells.." should be "tens of thousands of dividing cells".
- p.4, line 116: please be even more clear with what the "depleted" cells and controls are treated with: are depleted cells treated with both inducers, and controls with neither?
- p.5, lines 147-148: the authors write "the rate with which the abundance of Cox2 and Var1 proteins decreases was similar to the rate of mtDNA loss" though the actual rate is not shown. Please calculate and show rates for these processes side by side to make comparison possible, or alternatively rephrase the statement.

- Fig. 2d: changing the y-axis numbering to match those in panels a and b would facilitate comparisons.
- Fig. 2e: it is recommended to label the western blot panels to indicate what protein is being imaged in each (Neongree,, Mrps5, Qcr7).
- p.9, line 262: I suggest referencing Fig. 4e at the end of the first sentence for clarity.
- In the sections related to Fig. 3a and Fig. 5a as well as the connected supplemental data, the authors discuss both the median and the mean of mitochondrial mass and Atp6 protein, respectively. For purposes of clarity, I suggest decreasing the focus on the mean (that is provided only in the supplemental data) and focusing the text mainly on the median. The two show differing trends and it is very good that both are shown, but the clarity of the text can be improved by focusing more on the median where possible.
- p. 14, line 435: the statement that mt mass is maintained over the first 9h of depletion is only true for the mean mt mass, not for the median. Please make this clear or rephrase.
- p.14, line 452: "mitofusions" should be "mitofusins".

Referees cross-commenting

I think that the reviews of the other two reviewers are both insightful and constructive. Especially the rescue experiment suggested by Reviewer 2 could provide strong support for the interpretations of the study. Note that all three reviewers ask for validation of the use of Atp6p as a read-out of mtDNA function, and that all agree the data is powerful and the study of value to the field.

2. Significance:

Significance (Required)

The fact that disruption of mt fusion leads to mtDNA loss has been known for some time, but the mechanism behind this phenomenon has remained unknown to date. This thorough and precise study by Dengler et al uses state-of-the-art single-cell analysis to dissect the mechanisms underlying the mtDNA loss following the disruption of mt fusion, and convincingly reveal that it is caused by two different mechanisms: i) the inequal inheritance of mitochondria between mother and bud, and ii) the loss of a compensatory mechanism that normally maintains homeostatic mt protein levels. In the process, the authors shed light on the dynamics of the events following Fzo1 depletion, revealing dramatically fast mt fragmentation and a loss of MMP, which in turn can be expected to act as a stress signal and influence a number of cellular processes.

The findings of the study can have relevance for human conditions involving disrupted

mitochondrial dynamics, caused for example by mutations in mitofusins. The study will be of interest to researchers in mitochondrial biology ranging from dynamics and mtDNA maintenance to mitochondrial medicine.

The field of expertise of this reviewer: mtDNA maintenance. I am not able to properly evaluate the modelling in Fig. 7.

3. How much time do you estimate the authors will need to complete the suggested revisions:

Estimated time to Complete Revisions (Required)

(Decision Recommendation)

Less than 1 month

4. *Review Commons* values the work of reviewers and encourages them to get credit for their work. Select 'Yes' below to register your reviewing activity at Web of Science Reviewer Recognition Service (formerly Publons); note that the content of your review will not be visible on Web of Science.

Web of Science Reviewer Recognition

Yes

Review #2

1. Evidence, reproducibility and clarity:

Evidence, reproducibility and clarity (Required)

Dengler and colleagues use an AID-based acute depletion of Fzo1 in budding yeast, coupling microfluidics live imaging, single-cell quantification (>30k cells), proteomics, an mtDNA-encoded Atp6 reporter, and simple modeling to argue that fusion loss causes (i) rapid fragmentation and $\Delta\Psi m$ decline, (ii) progressive mtDNA/RC depletion, and (iii) unequal mother-daughter mitochondrial inheritance; together with a failure of compensatory synthesis, these changes drive petite formation. The time-resolved design is valuable, but several readouts are indirect, and some claims (particularly those regarding membrane potential, synthesis "failure," and causality) appear over-interpreted without additional controls.

Major points

1. While inducible TIR is used to reduce background, the manuscript should rigorously exclude auxin/TIR off-targets (growth, mitochondrial phenotypes, gene expression). Please include full matched controls: {plus minus}auxin, {plus minus}TIR, epitope tag alone, and a degron control on an unrelated mitochondrial membrane protein.
2. The Mitoloc preSu9 vs Cox4 import ratio is only a proxy of mitochondrial membrane potential (ΔΨm) and itself depends on mitochondrial mass, protein expression, matrix ATP, and import saturation. The authors need to calibrate ΔΨm with orthogonal dyes (TMRE/TMRM) and pharmacologic titrations (FCCP/antimycin/oligomycin) to generate a response curve; show that Mitoloc tracks dye-based ΔΨm across the relevant range and corrects for mass/photobleaching. Report single-cell ΔΨm vs mass residuals.
3. To use Atp6-mNeon as a proxy for mtDNA is an assumption. Interpreting Atp6 intensity as "functional mtDNA" could be confounded by translation, turnover, or assembly. Please (i) report mtDNA copy number time courses (you have qPCR), nucleoid counts (DAPI/PicoGreen or TFAM/Abf2 tagging), and (ii) assess translation (e.g., 35S-labeling or puromycin proxies) and turnover (proteasome/AAA protease inhibition, mitophagy mutants -some data are alluded to- plus mRNA levels for mtDNA-encoded genes). This will support the "reduced synthesis" versus "increased degradation" conclusion.
4. The promoter-NeonGreen reporters argue against transcriptional down-regulation of nuclear OXPHOS. Please add mRNA (RT-qPCR/RNA-seq) for representative genes and a pulse-chase or degradation-pathway dependency (e.g., proteasome/mitophagy/autophagy mutants) to firmly assign active degradation. The authors need to normalize proteomics to mitochondrial mass (e.g., citrate synthase/porin) to separate organelle abundance from protein turnover.
5. Using preSu9-mCardinal intensity as "mitochondrial concentration" is sensitive to expression, import competence, and morphology/segmentation. The authors should provide validation that this metric tracks 3D volume across fragmentation states (e.g., correlation with mito-GFP volumetrics; detergent-free CS activity; TOMM20/Por1 immunoblot per cell).
6. The unequal mother-daughter distribution is compelling, but causality remains inferred. Test whether modulating inheritance machinery (actin cables/Myo2, Num1, Mmr1) or altering fission (Dnm1 inhibition) modifies segregation defects and rescues mtDNA/Atp6 decline. Complementation with Fzo1 re-expression at defined times would help order the phenotype cascade.
7. The model is useful but should include parameter sensitivity (segregation variance,

synthesis slopes, initial nucleoid number) and prospective validation (e.g., predict rescue upon partial restoration of synthesis or inheritance, then test experimentally).

2. Significance:

Significance (Required)

The dataset is rich and the time-resolved approach strong, but key conclusions rely on indirect proxies and need orthogonal validation and at least one causal rescue experiment to avoid over-interpretation.

3. How much time do you estimate the authors will need to complete the suggested revisions:

Estimated time to Complete Revisions (Required)

(Decision Recommendation)

Between 3 and 6 months

4. *Review Commons* values the work of reviewers and encourages them to get credit for their work. Select 'Yes' below to register your reviewing activity at [Web of Science Reviewer Recognition Service] (formerly Publons); note that the content of your review will not be visible on Web of Science.

Web of Science Reviewer Recognition

Yes

Review #3

1. Evidence, reproducibility and clarity:

Evidence, reproducibility and clarity (Required)

This article addresses the connection between perturbed mitochondrial structure and genetics in yeast. When mitochondrial fusion is compromised, what is the chain of causality -- the mechanism -- that leads to mtDNA populations becoming depleted? This is a fascinating question, linking physical cell biology to population genetics. I admire the philosophy of the research, acknowledging and attempt to control for the many possible confounding influences. The manuscript describes the context and the research tightly and digestibly; the figures illustrate the results in a clear and natural way.

For transparency, I am Iain Johnston and I am happy for this review to be treated as public domain. To my eyes my most important shortcoming as a review is my relative lack of familiarity with the yeast fzo1 mutant; while I am familiar with analysis of yeast mito morphology and mtDNA segregation, a reviewer familiar with the nuances of this strain and its culture would be a useful complement.

I have a few more general points and a collection of smaller points below that I believe might help make the story more robust.

General points

1. About the use of Atp6 as a good proxy for mtDNA content. This is assumed from l285 onwards, based on a previous publication. As the link is fairly central to part of the paper's arguments, and the system in this study is being perturbed in several different ways, a stronger argument or demonstration that this link remains intact (and unchanged, as it is used in comparisons) would seem important.
2. About confounding variables and processes. The study does an admirable job of being transparent and attempting to control for the many different influences involved in the physical-genetic link. But some remain less clearly unpacked, including some I think could be quite important. For example, there is a lot of focus on mito concentration -- but given the phenotypes are changing the sizes of cells, do concentration changes come from volume changes, mito changes, or both? In "ruling out" mitophagy -- a potentially important (and intuitive) influence, the argument is not presented as directly as it could be and it's not completely clear that it can in fact be ruled out in this way. There are a couple of other instances which I've put in the smaller points below.

Smaller points

l47 full genus name when it first appears

l58 I may be wrong here, but I thought the petite phenotype more classically arises from mtDNA deletion mutations, not loss? The way this is phrased implies that mtDNA loss is [always] the cause. Whether I'm wrong on that point or not, the petite phenotype should be described and referenced.

para starting l59 -- should mention for context that mitochondria in (healthy, wildtype) yeast are generally much more fused than in other organisms

Fig 1C -- very odd choice of y-axis range! either start at zero or ensure that the data fill as much vertical space of the plot as possible

l105 "wild-type like more tubular mitochondria" reads rather awkwardly. "more tubular mitochondria (as in the wild-type)"?

l106 -- imaging artefacts? are mitos fragmenting because of photo stress? -- this is mentioned in l577-8 in the Methods, but the data from the growth rate and MMP comparison isn't given -- an SI figure would be helpful here. It would be reassuring to know that mito morphology wasn't changing in response to phototoxicity too.

para l146 -- so this suggests mtDNA-encoded proteins have a very rapid turnover, O(hours) -- is this known/reasonable?

section l189 -- it's hard to reason fully about these statistics of mitochondrial concentration given that the petite phenotype is fundamentally affecting overall cell volume. can we have details on the cell size distribution in parallel with these results? to put it another way -- how does mitochondrial *amount* per cell change?

l199 the mean in Fig S3C certainly does change -- it increases, clearly relative both to control and to its initial value. rather than sweeping this under the carpet we should look in more detail to understand it (a consequence of the increased skew of the distribution)?

para line 206 -- this doesn't make it clear whether your MMP signal is integrated over all mitochondria in the cell, or normalised by mitochondrial content? this matters quite a lot for the intepretation if the distributions of mitochondrial content are changing. reading on, this is even more important for para line 222.
Reading further on, there is an equation on l612 that gives a definition, but it doesn't really clarify (apologies if I'm misunderstanding).

l230 -- a point of personal interest -- low mito concentrations are connected to low "function" (MMP) and give extended division times -- this is interestingly exactly the model needed to reproduce observations in HeLa cells (https://journals.plos.org/ploscompbiol/article?id=10.1371/journal.pcbi.1002416). That model went on to predict several aspects of downstream cellular behaviour -- it would be

very interesting to see how compatible that picture (parameterised using HeLa observations) is with yeast!

l239 "less mitochondria" -- a bit tricky but I'd say "fewer mitochondria" or "less mitochondrial content"

Section l234
So here (and in Fig 4) the focus is on overall distributions of mitochondrial concentration in different cells (mother-to-be, mother, bud; gen 1, gen >1). But we've just seen that one effect of fzo1 is to broader the distribution of mitochondrial concentration across cells. Can't we look in more depth at the implications of this heterogeneity? For example in Fig 4F (which is cool) we look at the distribution of all fzo1 mothers-to-be, mothers, and buds. But this loses information about the provenance. For example, do mothers-to-be with extremely low mito concentrations just push everything to the bud, while mothers-to-be with high mito concentrations distribute things more evenly?
It would seem very easy and very interesting to somehow subset the distribution of mothers-to-be by concentration and see how different subsets behave

l285 -- experimental design -- do we know that Atp6 will continue to be a good proxy for functional mtDNA in the face of the perturbations provided by Fzo1 depletion? Especially if there is impact on the expression of mitoribosomes, the relationship between mtDNA and Atp6 may look rather different in the mutant?

l290 -- ruled out mitophagy. This message could be much clearer. Comparing Fig S5C and Fig 3A side-by-side is a needlessly difficult task -- put Fig 3A into Fig S5. Then we see that when mitophagy is compromised, the distribution of mitochondrial concentration has a lower median and much lower upper quartile than in the mitophagy-equipped Fzo1 mutant? What is going on here? For a paper motivated by disentagling coupled mechanisms, this should be made clearer!

With the Atp6 signal, how do we know that fluorescence from different cells is comparable? Buds will be smaller than mother cells for example, potentially leading to less occlusion of the fluorescent signal by other content in the cytoplasm

l336 -- similar to the Jajoo et al. mechanism in fission yeast -- but are you talking about feedback control of the mtDNA or the protein (or mRNA) product?

l343 -- maintenance of mtDNA -- here the point about l285 (is the Atp6-mtDNA relationship

the same in the Fzo1 mutant) is particularly important, as we're directly tying findings about the protein product to implications about the mtDNA

l367 -- on a first read this description of the model feels like lots of choices have been made without being fully justified. Why a log-normal distribution (when the fit to the data looks rather flawed); why the choice of 5 groups for nucleoid number (why not 3? or 8?); the process used for parameter fitting is very unclear (after reading the methods I think some of these values are read directly from the data, but the shapes of the distributions remain unexplained).
l705 -- presumably the ratio was drawn from a log-normal distribution and then the corresponding nucleoid numbers were rounded to integers? the ratio itself wasn't rounded? (also l367)
How were the log-normal distributions fitted to experiments (Figs. S7A,B)? Just by eye?
l711 by random selection -- just at random? ("selection" could be confusing)
Overall, it feels like the model may be too complicated for what it needs to show. Either (a) the model should show qualitatively that unequal inheritance and reduced production leads to rapid loss -- which a much simpler model, probably just involving a couple of lines of algebra, could show. Or (b) the model should quantitatively reproduce the particular numerical observations from the experiments -- it's not totally clear that it does this (do the cell-cycle-based decay timescales in Fig 7 correspond to the hour-based decay timescales in other plots, for example). At the moment the model is at a (b) level of detail but it's only clear that it's reporting the (a) level of results.

A lot of the discussion repeats the results; depending on editorial preferences some of this text could probably be pared back to focus on the literature connections and context.

Data availability -- it looks like much of the data required to reproduce the results is not going to be made available. Images and proteomic data are promised, but the data associated with mitochondrial concentration and other features are not mentioned. For FAIR purposes all the data (including statistics from analysis of the images) should be published.

l660 -- can an overview of the EM protocol be given, to avoid having to buy the Mayer 2024 article?

2. Significance:

Significance (Required)

This is a powerful and thoughtful study that provides a collection of new mechanistic insights into the link between physical and genetic properties of mitochondria in yeast. Cell biologists, geneticists, and the mitochondrial field will find this of potentially deep interest. Because of the mode and dynamics of inheritance in budding yeast, findings here may not be directly transferrable to other eukaryotes, but these insights are still of interest for researchers outside of yeast for their insight into how this well-studied system manages its mitochondrial populations.

3. How much time do you estimate the authors will need to complete the suggested revisions:

Estimated time to Complete Revisions (Required)

(Decision Recommendation)

Between 1 and 3 months

4. *Review Commons* values the work of reviewers and encourages them to get credit for their work. Select 'Yes' below to register your reviewing activity at [Web of Science Reviewer Recognition Service] (formerly Publons); note that the content of your review will not be visible on Web of Science.

Web of Science Reviewer Recognition

Yes

1st Editorial Decision 17th Oct 2025

Dear Prof. Ewald

Thank you for the submission of your research manuscript to our journal. I have now read your manuscript, the referee reports and your point-by-point response and agree that your study might make an interesting contribution to EMBO reports.

I would therefore like to invite you to revise your study along the lines proposed in your response to the referees. Your revised manuscript will then be seen again by the original referees. Acceptance of the manuscript will depend on a positive outcome of a second round of review. It is EMBO Reports policy to allow a single round of revision only and acceptance or rejection of the manuscript will therefore depend on the completeness of your responses included in the next, final version of the manuscript.

I noticed the comments on source data availability (Referee #3). You state that the proteomics data and the model are already fully accessible, which is great. Please include information and the link to these datasets/model in a dedicated Data Availability section (see point 7 below). Any other data used to generate figures can (and must be) provided as source data (see point 8 below). This will allow you to share Western blot and Ponceau staining data. The imaging data could be deposited in a public repository, but I would understand if there are size limits.

We realize that it is difficult to revise to a specific deadline. In the interest of protecting the conceptual advance provided by the work, we recommend a revision within 3 months (Janury 18th, 2026). Please discuss the revision progress ahead of this time with the editor if you require more time to complete the revisions.

I am also happy to discuss the revision further via e-mail or a video call, if you wish.

You can either publish the study as a short report or as a full article. For short reports, the revised manuscript should not exceed 27,000 characters (including spaces but excluding materials & methods and references) and 5 main plus 5 expanded view figures. The results and discussion sections must further be combined, which will help to shorten the manuscript text by eliminating some redundancy that is inevitable when discussing the same experiments twice. For a normal article there are no length limitations, but it should have more than 5 main figures and the results and discussion sections must be separate. In both cases, the entire materials and methods must be included in the main manuscript file.

================

IMPORTANT NOTE:

We perform an initial quality control of all revised manuscripts before re-review. Your manuscript will FAIL this control and the handling will be delayed IN CASE the following APPLIES:

1) A data availability section providing access to data deposited in public databases is missing. If you have not deposited any data, please add a sentence to the data availability section that explains that.

2) Your manuscript contains statistics and error bars based on n=2. Please use scatter blots in these cases. No statistics should be calculated if n=2.

When submitting your revised manuscript, please carefully review the instructions that follow below. Failure to include requested items will delay the evaluation of your revision.

================

When submitting your revised manuscript, we will require:

1) a .docx formatted version of the manuscript text (including legends for main figures, EV figures and tables). Please make sure that the changes are highlighted to be clearly visible.

2) individual production quality figure files as .eps, .tif, .jpg (one file per figure).
Please download our Figure Preparation Guidelines (figure preparation pdf) from our Author Guidelines pages https://www.embopress.org/page/journal/14693178/authorguide for more info on how to prepare your figures.

3) a .docx formatted letter INCLUDING the reviewers' reports and your detailed point-by-point responses to their comments. As part of the EMBO Press transparent editorial process, the point-by-point response is part of the Review Process File (RPF), which will be published alongside your paper.

4) a complete author checklist, which you can download from our author guidelines (). Please insert information in the checklist

that is also reflected in the manuscript. The completed author checklist will also be part of the RPF.

5) Please note that all corresponding authors are required to supply an ORCID ID for their name upon submission of a revised manuscript (). Please find instructions on how to link your ORCID ID to your account in our manuscript tracking system in our Author guidelines
()

6) We replaced Supplementary Information with Expanded View (EV) Figures and Tables that are collapsible/expandable online. A maximum of 5 EV Figures can be typeset. EV Figures should be cited as 'Figure EV1, Figure EV2" etc... in the text and their respective legends should be included in the main text after the legends of regular figures.

- For the figures that you do NOT wish to display as Expanded View figures, they should be bundled together with their legends in a single PDF file called *Appendix*, which should start with a short Table of Content. Appendix figures should be referred to in the main text as: "Appendix Figure S1, Appendix Figure S2" etc. See detailed instructions regarding expanded view here:

- Additional Tables/Datasets should be labeled and referred to as Table EV1, Dataset EV1, etc. Legends have to be provided in a separate tab in case of .xls files. Alternatively, the legend can be supplied as a separate text file (README) and zipped together with the Table/Dataset file.

7) Before submitting your revision, primary datasets (and computer code, where appropriate) produced in this study need to be deposited in an appropriate public database (see <
https://www.embopress.org/page/journal/14693178/authorguide#dataavailability>).

Please remember to provide a reviewer password if the datasets are not yet public.

The accession numbers and database should be listed in a formal "Data Availability " section (placed after Materials & Method) that follows the model below (see also < https://www.embopress.org/page/journal/14693178/authorguide#dataavailability>). Please note that the Data Availability Section is restricted to new primary data that are part of this study.

Data availability

The datasets (and computer code) produced in this study are available in the following databases:

- RNA-Seq data: Gene Expression Omnibus GSE46843 (https://www.ncbi.nlm.nih.gov/geo/query/acc.cgi?acc=GSE46843)
- [data type]: [name of the resource] [accession number/identifier/doi] ([URL or identifiers.org/DATABASE:ACCESSION])

*** Note - All links should resolve to a page where the data can be accessed. ***

8) At EMBO Press we ask authors to provide source data for the main manuscript figures. You will receive a separate email with instructions for providing source data with your revised manuscript, including how to upload and organize the files.

Additional information on source data and instruction on how to label the files are available

9) The journal requires a statement specifying whether or not authors have competing interests (defined as all potential or actual interests that could be perceived to influence the presentation or interpretation of an article). In case of competing interests, this must be specified in your disclosure statement. Further information: https://www.embopress.org/competing-interests

10) Figure legends and data quantification:
The following points must be specified in each figure legend:

- the name of the statistical test used to generate error bars and P values,
- the EXACT p-values,
- the number (n) of independent experiments (please specify technical or biological replicates) underlying each data point,
- the nature of the bars and error bars (s.d., s.e.m.)

- If the data are obtained from n {less than or equal to} 5, show the individual data points in addition to the SD or SEM.
- If the data are obtained from n {less than or equal to} 2, use scatter blots showing the individual data points.

Discussion of statistical methodology can be reported in the materials and methods section, but figure legends should contain a basic description of n, P and the test applied.

See also the guidelines for figure legend preparation:
https://www.embopress.org/page/journal/14693178/authorguide#figureformat

- Please also include scale bars in all microscopy images and define their size in the legend, not the image.

11) Our journal encourages inclusion of *data citations in the reference list* to directly cite datasets that were re-used and obtained from public databases. Data citations in the article text are distinct from normal bibliographical citations and should directly link to the database records from which the data can be accessed. In the main text, data citations are formatted as follows: "Data ref: Smith et al, 2001" or "Data ref: NCBI Sequence Read Archive PRJNA342805, 2017". In the Reference list, data citations must be labeled with "[DATASET]". A data reference must provide the database name, accession number/identifiers and a resolvable link to the landing page from which the data can be accessed at the end of the reference. Further instructions are available at .

12) All Materials and Methods need to be described in the main text using our 'Structured Methods' format. According to this format, the Methods section includes a Reagents and Tools Table (listing key reagents, experimental models, software and relevant equipment and including their sources and relevant identifiers) followed by a Methods and Protocols section describing the methods, ideally using a step-by-step protocol format. The aim is to facilitate adoption of the methodologies across labs. Please download and fill our Reagents and Tools Table template (.docx), which you can find in our author guidelines: https://www.embopress.org/page/journal/14693178/authorguide#structuredmethods.
When submitting your revised manuscript, please do not include the Reagents and Tools Table in the Methods section of the manuscript but upload it as a separate file choosing the file type "Reagent Table".
An example of a Method paper with Structured Methods can be found here: https://www.embopress.org/doi/10.15252/msb.20178071.

13) As part of the EMBO publication's Transparent Editorial Process, EMBO Reports publishes online a Review Process File to accompany accepted manuscripts. This File will be published in conjunction with your paper and will include the referee reports, your point-by-point response and all pertinent correspondence relating to the manuscript.

You are able to opt out of this by letting the editorial office know (emboreports@embo.org). If you do opt out, the Review Process File link will point to the following statement: "No Review Process File is available with this article, as the authors have chosen not to make the review process public in this case."

We would also welcome the submission of cover suggestions, or motifs to be used by our Graphics Illustrator in designing a cover.

I look forward to seeing a revised form of your manuscript when it is ready and please let me know if you have questions or comments regarding the revision.

Yours sincerely,

Martina Rembold, PhD
Senior Editor
EMBO reports

Response to Reviewers:

When mitochondria fall apart: Unbalanced mitochondrial segregation triggers loss of mtDNA in the absence of mitochondrial fusion

Lisa Dengler[1], Francesco Padovani[2], Bianca Lemke[3], Rebecca Brugger[4], Alissa Benedikt[2], Benedikt Westermann[4], Boris Maček[3], Kurt M. Schmoller[2], Jennifer C. Ewald[1]

We thank the reviewers for their thoughtful comments and overall very supportive feedback.

We believe we have addressed all points, as detailed below.

<u>Reviewer 1:</u>

1.1 Statistical comparisons are missing throughout the manuscript (with the exception of Fig. 2c). Appropriate statistical tests, along with p-values, should be used and reported where different gorups are compared, for example (but not limited to) Fig. 3d and most panels of Fig. 4.

We initially decided not to add too many extra labels to the already very busy plots, given that the magnitude of change mostly speaks for itself. However, we have now added statistical tests (especially figure 3D and 4) wherever we think it makes sense and report p-values in the figure legends.

1.2. I do not agree with the use of Atp6 protein as a direct read-out of mtDNA content. While Atp6 protein levels will decrease with decreasing mtDNA content, the inverse is not necessarily true: decreased Atp6 protein levels do not necessarily indicate decreased mtDNA levels, because they could alternatively or additionally be caused by decreased transcription and/or translation. Therefore, please do not equate Atp6 protein levels to mtDNA levels, and instead rephrase the text referencing the Atp6 experiments in the Results and Discussion sections to measure "mtDNA expression" or "mt-encoded protein" or similar. For example, on p. 14 line 431 should read "mtDNA expression" rather than "decreased synthesis of mtDNA", and line 440 on the same page "mean mtDNA levels" should be "mtDNA expression" or similar.

All three reviewers agree that using Atp6-NG as a direct proxy for mtDNA requires more validation. We agreed that this is the most important point to address. We had previously tried using an mtDNA LacO array-LacI-system (Osman et al., 2015) to directly assess the amount of nucleoids per cell. However, the altered mitochondrial morphology of the Fzo1 depleted cells combined with the LacI-GFP which is still in mitochondria even when mtDNA is gone, increases the noise level to a point that we cannot interpret the signal. However, as this manuscript was in the submission process, the Schmoller lab (co-authors #2 and #7) adapted the HI-NESS system to label mtDNA in live yeast cells (Deng et al., 2025). This system shows much better signal to noise and we now show both nucleoids counts and Atp6 in figure 5B and EV5B. Reassuringly, the decrease in Atp6 directly follows the drop in nucleoid counts.

1.3. In Fig. 3, the authors use the fluorescence intensity of a mitochondrially-targeted mCardinal as a read-out of mitochondrial mass. Please provide evidence that this is not affected by MMP, either with relevant references or by control experiments (e.g. comparing it to N-acridine orange or other MMP-independent dyes or methods).

Whether or not the import of any mitochondrial protein is dependent on the MMP depends largely on the signal sequence. The preSu9 mitochondrial targeting sequence was previously characterized as largely independent of the MMP compared to other presequences (Martin et al., 1991), which is why Vowinckel (Vowinckel et al., 2015) and others (Di Bartolomeo et al., 2020; Ebert et al., 2025; Perić et al., 2016) have previously used this as a neutral reference to the strongly MMP-dependent pre-Cox4 signal to estimate MMP. As one control in our own data, we consider that the population-averaged mitochondrial fluorescent signal Figure EV3D stays constant in the first few hours, in agreement with the total averaged mitochondrial proteome (Fig S1). Additionally, we now show that the preSu9 signal correlates well with Tom70-Neongreen, a protein of the outer membrane that is often used to quantify mitochondrial content (EV3C). Supporting the use of Tom70 as a marker protein, it does not decrease in our proteomic dataset (Suppl T8).

1.4. In Fig. 2e-f, the authors use a promoter reporter with Neongreen to answer whether the reduced levels of the nuclear-encoded mitochondrial proteins Mrps5 and Qcr7 are due to decreased expression or to protein degradation, and find no evidence of degradation of the Neongreen reporter protein. However, subcellular localization might affect the availability of the protein to proteases. Although not absolutely required, it would be relevant to know if the Neongreen fusion protein is found in the same subcellular compartment as Mrps5 and Qcr7 at 0h and 9h after Fzo1 depletion.

Here, it seems we need to explain the set-up and interpretation of the data better, which we did by adding an extra sentence to the corresponding section. The key point we are trying to make with the promoter-Neongreen construct is that the regulation is not mainly at the level of transcription. We are showing that the reduction in the levels of the actual protein (orange bars) is not (mainly) explained by a reduction in expression, since the promoter is similarly active at 0 and at 9 hours (grey bars). If expression from the promoter were strongly reduced, the Neongreen would be diluted with growth and would also decrease, but this is not the case. The fluorophore itself is just floating around in the cytosol and is not subject to the same post-translational regulation as Mrps5 and Qcr7, so there is no reason to expect degradation.

To support this data, we have now added RT-qPCR data for Mrps5 and Qcr7. In line with our reporter data, the RNA levels of Mrps5 remain constant after Fzo1 depletion (and even in the *fzo1* deletion), see Fig EV2I. This supports the model that Mrps5 proteins are reduced by degradation rather than by reduced expression. However, to our surprise, the RNA levels of Qrc7 did decrease, indicating a more complex regulation on transcriptional or RNA-stability level that is not captured by the promoter sequence alone. We have extended the text in our results section accordingly. This observation is very interesting and warrants further investigations, but this is outside the scope of the current manuscript.

1. 5. Fzo1 depletion leads to a very rapid drop in MMP during the first hour of depletion. In the Discussion, can the authors speculate on the possible mechanism of this rapid MMP drop that occurs well before mtDNA or mt-encoded proteins are decreased in level?

This is indeed an interesting point. We think there are likely three reasons causing this initial drop: Firstly, due to the fragmentation the mixing of mitochondrial content is disturbed and smaller fragments may have suboptimal stoichiometry of components (see also (Khan et al., 2024) who look at this in detail including the Fzo1 deletion); secondly, already fairly early, some mitochondrial fragments may not contain any mtDNA and therefore will be unable to synthesize mtDNA-encoded ETC proteins and therefore ETC function decreases; thirdly, altered morphological features like changes in the surface-to-volume ratios may play a role. We briefly comment on this in the discussion, but do not want to speculate too much, since we cannot follow-up on this within the scope of the manuscript.

1. 6. In Fig. 2a, the mtDNA copy number of Fzo1-depleted cells is ca 1.3-fold of the control cells at the 0h timepoint. Why might this be? Is it an impact of one of the inducers? If so, we might be looking at the combination of two different processes when measuring copy number: one that is an induction caused by the inducer(s), and the other a consequence of Fzo1 depletion itself.

We believe that this 30% increase is within the noise of the experiment rather than an effect of the induction. Since we normalize to t=0 uninduced, the first black data point does not have error bars, overemphasizing the difference. None of the protein data suggests that there is an increase in mtDNA-encoded proteins (see e.g. Fig 2B, or Atp6 fluorescence data). We also ran qPCRs on additional controls showing that adding the inducers in the presence of the TIR protein as well as the AID-Fzo1 tagged strain after addition of aTC shows comparable mtDNA levels as an untreated WT (EV 1E).

Minor comments:

1.7. p. 3, line 71: "ten thousands of dividing cells.." should be "tens of thousands of dividing cells".

Thank you, we corrected that.

1.8.-p.4, line 116: please be even more clear with what the "depleted" cells and controls are treated with: are depleted cells treated with both inducers, and controls with neither?

We have illustrated this more clearly now in figure 1 and EV1. Depleted cells are treated with both inducers, the control cells are not. However, in Figure 1A and in EV1 we do controls to show that inducing TIR per se or adding aTC per se does not change growth rate (EV1D) or mitochondrial morphology (EV1B,C).

1.9. -p.5, lines 147-148: the authors write "the rate with which the abundance of Cox2 and Var1 proteins decreases was similar to the rate of mtDNA loss" though the actual rate is not shown. Please calculate and show rates for these processes side by side to make comparison possible, or alternatively rephrase the statement.

Indeed this was not phrased well. We now call it dynamics rather than rates. Given that these are different methods and the rates are not actually constant, there is no reason to try to quantitatively compare rates.

1.10. -Fig. 2d: changing the y-axis numbering to match those in panels a and b would facilitate comparisons.

Done.

1.11. Fig. 2e: it is recommended to label the western blot panels to indicate what protein is being imaged in each (Neongree,, Mrps5, Qcr7).

We have adapted the labelling.

1.12. -p.9, line 262: I suggest referencing Fig. 4e at the end of the first sentence for clarity.

Done.

1.13. -In the sections related to Fig. 3a and Fig. 5a as well as the connected supplemental data, the authors discuss both the median and the mean of mitochondrial mass and Atp6 protein, respectively. For purposes of clarity, I suggest decreasing the focus on the mean (that is provided only in the supplemental data) and focusing the text mainly on the median. The two show differing trends and it is very good that both are shown, but the clarity of the text can be improved by focusing more on the median where possible.

This section has changed a bit since we include the nucleoids. The decrease of the mean is noteworthy because it is different to the mitochondrial concentrations. This is also relevant if you compare it to the population level data. We hope the relevance becomes clearer now.

1.14. -p. 14, line 435: the statement that mt mass is maintained over the first 9h of depletion is only true for the mean mt mass, not for the median. Please make this clear or rephrase.

We have made this more clear and have also added more graphs on the proteomics data (Fig S1).

1.15.-p.14, line 452: "mitofusions" should be "mitofusins".

Thanks for catching this.

<u>Reviewer 2:</u>

2. 1. While inducible TIR is used to reduce background, the manuscript should rigorously exclude auxin/TIR off-targets (growth, mitochondrial phenotypes, gene expression). Please include full matched controls: (plus minus)auxin, (plus minus)TIR, epitope tag alone, and a degron control on an unrelated mitochondrial membrane protein.

We agree that rigorous controls are crucial for the interpretation of the results. However, we think we had already included most of the controls the reviewer is asking for, but we might have not pointed this out clearly enough. For example, in Fig 1A, we have made it clearer by adding more labels directly to the figure instead of just the figure legend. We have added a few extra controls that are reported in EV1.

Here is a list of all the controls:

- Each depletion experiment is always matched with an experiment of the same strain with AID-Fzo1 tag and TIR without addition of inducers. So, the genetic background as well as effects such as light exposure, time spent in the microfluidics systems, etc. are controlled for.
- Figure EV1D shows that the growth rate is wildtype-like in a strain containing either the AID tag on Fzo1 or the TIR protein AND upon addition of both chemicals if either the TIR protein or only the AID-tag on Fzo1 are present. It also shows that the final genetic background (AID-tag and TIR) also grows like wildtype if the inducers are not added. This conclusively shows that neither the tags/constructs nor the chemicals per se affect growth rate.
- In Figure EV1C,D we show the mitochondrial morphology of the same controls. We have now labelled them more consistently between panels.
- In figure 1A we compare the Fzo1 protein levels of a strain with and without TIR. We show that in absence of TIR, adding either aTC or Auxin does not change Fzo1 levels and that the levels are comparable in the strain that is able to deplete Fzo1 directly before addition of 5-Ph-IAA (so after 2 h of induction of TIR through addition of anhydrotetracycline)
- Additionally, in Figure EV2C we show that two hours after adding aTC, the entire proteome does not change significantly apart from a strong induction of TIR. We have made this clearer in the figure legend.
- Additionally, we have run a qPCR to carefully determine mtDNA levels of untreated wild-type cells, tetracycline treated wild-type cells and tetracycline induced TIR expressing cells to exclude effects of tetracycline as well as the expression of TIR on mtDNA. (also in response to 1.6.), see Figure EV1E.

In summary, we think we have controlled sufficiently for all confounding parameters and most importantly showed that addition of either aTC or Auxin as well as the FLAG-AID tag per se do not disturb mitochondria or cell growth. We do not see what a degron control on an unrelated protein will tell us. Depending on the nature of the protein, it may or may not have a phenotype that may or may not be related to morphology changes etc. Therefore, after discussion with the editor, we have not included such a strain.

2. 2. The Mitoloc preSu9 vs Cox4 import ratio is only a proxy of mitochondrial membrane potential (ΔΨm) and itself depends on mitochondrial mass, protein expression, matrix ATP, and import saturation. The authors need to calibrate ΔΨm with orthogonal dyes (TMRE/TMRM) and pharmacologic titrations (FCCP/antimycin/oligomycin) to generate a response curve; show that

Mitoloc tracks dye-based ΔΨm across the relevant range and corrects for mass/photobleaching. Report single-cell ΔΨm vs mass residuals.

We completely agree that the MitoLoc system is only a rough proxy for the actual membrane potential. That is why we make no quantitative claims on the absolute value or absolute difference between groups of cells. We also make very clear in Fig 3B what we are actually measuring.

We agree that it is a good idea to compare MitoLoc values to TMRM staining as the reviewer suggests. Please note though that also dye staining has its caveats and cannot be used in dynamic live cell experiments since the staining rapidly decreases within about 15 minutes after washing steps were performed. Additionally, TMRM is not compatible with the acidic pH 5 medium that is typically used for yeast but requires staining at neutral pH which affects yeasts cell viability quickly. We have therefore decided to use TMRM and compare it to Tom70-Neongreen as an MMP independent reference 8 h after Fzo1 depletion to minimize the effect of the time required for staining, washing and imaging. Fig EV 3H shows that eight hours after depletion, where the MMP sensor almost reaches Δ*fzo1* levels, the TMRM signal is close to detection limit, in agreement with the genetically encoded MMP sensor.

We had already plotted MMP vs mitochondrial concentration, this is now shown in Fig EV4G.

In agreement with the editor, we have not done elaborate pharmacological titration experiments because firstly, this was extensively done in the original MitoLoc paper by the Ralser lab where the authors performed titration experiments with different CCCP concentrations and timings, and compared CCCP treatments of rho^0 cells and Δ*fzo1* cells ((Vowinckel et al., 2015), which has been cited 120 times, i.e. has been used by many other groups); secondly, the value of the MMP is not the most critical claim of the manuscript.

2.3. To use Atp6-mNeon as a proxy for mtDNA is an assumption. Interpreting Atp6 intensity as "functional mtDNA" could be confounded by translation, turnover, or assembly. Please (i) report mtDNA copy number time courses (you have qPCR), nucleoid counts (DAPI/PicoGreen or TFAM/Abf2 tagging), and (ii) assess translation (e.g., 35S-labeling or puromycin proxies) and turnover (proteasome/AAA protease inhibition, mitophagy mutants -some data are alluded to- plus mRNA levels for mtDNA-encoded genes). This will support the "reduced synthesis" versus "increased degradation" conclusion.

We agree with all three reviewers that Atp6 is only a proxy for mtDNA (Jakubke et al., 2021; Roussou et al., 2024) and the correlation should be checked more carefully. We have now used the recently established HI-NESS system to follow nucleoids/mtDNA during depletion experiments. See detailed reply to 1.2. The Atp6 decline over time directly follows the nucleoid counts (Fig 5A, EV5B) and mtDNA decline (Fig 2A) with a small delay.

In Figure 2C we inhibit mitochondrial translation and show that in this case control and depleted cells have the same level of Cox2, at least suggesting that degradation is not the key mechanism controlling the levels of mtDNA encoded proteins. We cannot do proteasome inhibitor assays since the nature of the AID-TIR systems requires an active proteasome. Also, mtDNA-encoded mitochondrial inner membrane proteins such as Atp6 are not be accessible to the proteasome but are targeted by mitochondrial proteases which are not straightforward to target.

In figure EV5C,D we show that the mitochondrial concentration and Atp6 concentration are similar in an *atg32* deletion. This does not completely exclude a contribution of mitophagy to the observed phenotype, but does confirm that mitophagy is not the primary reason for cells becoming petite.

2. 4. The promoter-NeonGreen reporters argue against transcriptional down-regulation of nuclear OXPHOS. Please add mRNA (RT-qPCR/RNA-seq) for representative genes and a pulse-chase or degradation-pathway dependency (e.g., proteasome/mitophagy/autophagy mutants) to firmly assign active degradation. The authors need to normalize proteomics to mitochondrial mass (e.g., citrate synthase/porin) to separate organelle abundance from protein turnover.

In agreement with the editor, we think an RNA-seq experiment is outside the scope of this study. The mechanism coordinating nuclear and mitochondrial proteins is just a minor side aspect of our story and does not warrant additional major experiments.

Nevertheless, we have added a RT-qPCR experiment where we quantify the RNA of Qrc7 and Mrps5. The RNA levels of Mrps5 remained constant after Fzo1 depletion (and even in the *fzo1* deletion), see Fig EV2I, confirming the data from the fluorescent reporter. This supports the model that Mrps5 proteins are reduced by degradation rather than by reduced expression. However, to our surprise, the RNA levels of Qrc7 did decrease, indicating a more complex regulation on transcriptional or RNA-stability level, that is not captured by the promoter sequence alone. We have extended the text in our results section accordingly. This observation is very interesting and warrants further investigations, but this is outside the scope of the current manuscript.

As explained above, a proteasome inhibitor experiment is not possible in this set-up. Bulk mitophagy/autophagy seems unlikely to be the cause of the decrease of the nuclear-encoded OXPHOS proteins, since most other mitochondrial proteins do not decrease on average on population level in the first hours (additional figure S1), and the *atg32* deletion does not affect the phenotype (Fig EV5C,D).

2. 5. Using preSu9-mCardinal intensity as "mitochondrial concentration" is sensitive to expression, import competence, and morphology/segmentation. The authors should provide validation that this metric tracks 3D volume across fragmentation states (e.g., correlation with mito-GFP volumetrics; detergent-free CS activity; TOMM20/Por1 immunoblot per cell).

We agree that this is an important point and the co-authors discussed this point quite intensively. In the figure S3A,B we show (using confocal data) that there is a very strong correlation between the total fluorescence signal and the 3D volume reconstruction. However, the slope of the correlation is different between tubular and fragmented mitochondria (compare panels A and B) and see figure legend. Since we are dealing with diffraction-limited objects it is likely that the 3D reconstruction is sensitive to morphology, especially if mitochondria are “clumping”. We therefore think that the total fluorescence signal is actually a better estimate of mitochondrial mass per cell than the 3D volume reconstruction (especially for our data obtained with a conventional epifluorescence microscope).

The mean of the total mitochondrial fluorescence best matches the population average mitochondrial proteome (Fig S1). To consolidate this, we additionally constructed a strain with the outer membrane protein Tom70-Neongreen which is not decreasing in our proteomics dataset (Table S8) in addition to preSu-Cardinal. Fig EV3C shows that these two correlate well.

Notably, since the morphology is similarly altered in mothers and buds this is of minor impact for our main point – the unequal distribution between mother and buds.

2.6. The unequal mother-daughter distribution is compelling, but causality remains inferred. Test whether modulating inheritance machinery (actin cables/Myo2, Num1, Mmr1) or altering fission (Dnm1 inhibition) modifies segregation defects and rescues mtDNA/Atp6 decline. Complementation with Fzo1 re-expression at defined times would help order the phenotype cascade.

We agree that rescue experiments would be very useful. We had some preliminary data for tether experiments, for example with an artificial construct driven by an inducible promoter to tether mitochondria to the mother cell cortex using the PH-domain of Num1 connected to an OMM protein. However, the general problem is that the fragmented mitochondria clump together and it was not possible to find expression levels of this construct that resulted in an even distribution. We have not found a method to restore an equal distribution between mother and daughter cells.

fzo1/dmn1 double deletions have been done previously (Wisniewski et al., 2024), these mutants have an almost normal mitochondrial morphology but still become petite (~70 %), however they only have a slight reduction in mtDNA levels. Therefore, the reason why those cells become petite likely differs from that of cells lacking only Fzo1.

mmr1 deletion aggravates the *fzo1* phenotype (as already evidenced by a negative SGA interaction, (Böckler et al., 2017)), likely also because the distribution becomes even more heterogeneous.

We liked the idea of the Fzo1 re-expression and ran such an experiment which we analysed in detail and dedicated an entire new figure to these interesting results Fig 7, EV7. We removed the auxin after six hours of Fzo1 depletion and observed mitochondrial morphology, mitochondrial inheritance, and Atp6 concentrations in the following two generations. Notably, the mitochondrial morphology starts recovering rather quickly, regardless of the Atp6 concentration at the time of re-addition, indicating that morphology is (as expected) the primary, but reversible phenotype of Fzo1 depletion. As the morphology recovers, the distribution between mothers and buds gradually becomes more wildtype like. The Atp6 concentration, however, only recovers in some cells. Those cells with a critically low concentration do not recover, indicating that these cells had lost all functional mtDNA, i.e. are irreversibly petite.

2. 7. The model is useful but should include parameter sensitivity (segregation variance, synthesis slopes, initial nucleoid number) and prospective validation (e.g., predict rescue upon partial restoration of synthesis or inheritance, then test experimentally).

We have added figure EV8 with a detailed sensitivity analysis and explained this in more detail in the method section. We performed additional simulations and discuss parameter sensitivity. The simulation results are robust to changes in the initial nucleoid number. In addition to the original

16 nucleoids, we now show simulations with 12 and 20 nucleoids, which qualitatively lead to the same result (Fig EV8C). We also performed simulations in which we increased or decreased the width of the lognormal distribution setting the nucleoid segregation by a factor 1.5 (for both the WT and mutant-like distribution). As expected, these changes affect the temporal evolution of the nucleoid number: In particular, a lower variance slows down the decline in the nucleoid number observed in the mutant condition. Accordingly, increasing the variance leads to a faster loss in the mutant and also causes a slow decline in the WT condition (Fig 8D).

We also performed the simulation using a gamma distribution to fit the ratios of Atp6 of the daughter / mother and obtained very similar results (Fig EV8F).

Finally, also in response to reviewer 3, we now clarify that the mtDNA-dependent synthesis rate is a critical feature of the model. If instead we assume a constant relative synthesis rate for each cell, the distribution of nucleoids is not stable, even in the WT condition. In fact, our choice to include this mtDNA-dependent synthesis in the model was guided by strategy when building the model, which was to keep it as simple as possible while including sufficient detail to 1.) achieve nucleoid numbers that are stable in the WT condition on the time-scale of the simulation and 2.) capture the major differences between WT and mutant that we observed experimentally (Fig 8E).

In the revised manuscript, we now also include new experiments testing the reversibility of the phenotype if Fzo1 is re-expressed after 6 h hours of depletion. We find that the effect on mitochondrial morphology is reversible. However, cells with very low Atp6 expression do not recover, which is consistent with our conclusion that Fzo1 depletion leads to a petite phenotype due to stochastic loss of mtDNA, which is then irreversible (Fig 7, EV7).

Reviewer 3:

3.1. About the use of Atp6 as a good proxy for mtDNA content. This is assumed from l285 onwards, based on a previous publication. As the link is fairly central to part of the paper's arguments, and the system in this study is being perturbed in several different ways, a stronger argument or demonstration that this link remains intact (and unchanged, as it is used in comparisons) would seem important.

We have now added experiments using the HI-NESS system to directly quantify the number of nucleoids in live cells. See 1.2. for details.

3.2. About confounding variables and processes. The study does an admirable job of being transparent and attempting to control for the many different influences involved in the physical-genetic link. But some remain less clearly unpacked, including some I think could be quite important. For example, there is a lot of focus on mito concentration -- but given the phenotypes are changing the sizes of cells, do concentration changes come from volume changes, mito changes, or both? In "ruling out" mitophagy -- a potentially important (and intuitive) influence, the argument is not presented as directly as it could be and it's not completely clear that it can in fact be ruled out in this way. There are a couple of other instances which I've put in the smaller points below.

Thank you for acknowledging our efforts to show transparent and well-controlled experiments! We address each of the specific points below.

3.3. full genus name when it first appears

Done

3.4. I may be wrong here, but I thought the petite phenotype more classically arises from mtDNA deletion mutations, not loss? The way this is phrased implies that mtDNA loss is [always] the cause. Whether I'm wrong on that point or not, the petite phenotype should be described and referenced.

The term “petite” refers to any strain that is respiratory incompetent which leads to small colonies (not necessarily small cells! (Seel et al., 2023)) on media containing limiting amounts of fermentable carbon sources. This can be caused by mutations or gene loss on the mtDNA (these are called cytoplasmic petite), or chemically induced loss of the entire mtDNA (e.g. by EtBr), or mutations of nuclear genes required for respiration (these are termed nuclear petite; some nuclear petites show loss of mtDNA in addition to the mutation in the nuclear genome) (Contamine & Picard, 2000). We now cite additional papers in the introduction.

3.5. para starting l59 -- should mention for context that mitochondria in (healthy, wildtype) yeast are generally much more fused than in other organisms

Done.

3.6. Fig 1C -- very odd choice of y-axis range! either start at zero or ensure that the data fill as much vertical space of the plot as possible

True, this was probably some formatting relic. We have adapted the axis to fill the full space. Most of our axes start at 0, but that does not make so much sense here, since we consider the solidity in the control as “baseline”.

3.7. "wild-type like more tubular mitochondria" reads rather awkwardly. "more tubular mitochondria (as in the wild-type)"?

Thank you, sounds better.

3.8. l106 -- imaging artefacts? are mitos fragmenting because of photo stress? -- this is mentioned in l577-8 in the Methods, but the data from the growth rate and MMP comparison isn't given -- an SI figure would be helpful here. It would be reassuring to know that mito morphology wasn't changing in response to phototoxicity too.

In the methods we just briefly point out that we have done all our “due diligence” controls to check that we do not generate phototoxicity, something that we highlight in the cited review. We

do not explicitly have a figure for this, but figure EV1A shows that the solidity of the mitochondrial network in control cells stays the same over 9 hours, even though these cells are exposed to the same cultivation and imaging regime as the depleted cells. In EV1C we show that control cells containing TIR but no AID tag treated with both chemicals imaged over 9 hours also show the same solidity (~mitochondrial morphology) as untreated control (=AID-Fzo1, TIR). Also, the doubling times of cells grown in our imaging system (Fig EV1G) are very similar to the shake flask (Fig EV1F). All in all, we are very confident that our imaging settings did not impact our reported phenotypes and now also refer to Fig EV1 in the method section.

3.9. para l146 -- so this suggests mtDNA-encoded proteins have a very rapid turnover, O(hours) -- is this known/reasonable?

Reference (Christiano et al., 2014) suggests that respiratory chain proteins are shorter lived than the average yeast protein. However, based on Figure 2C we think the dynamics mostly speak for a dilution by growth.

3.10. section l189 -- it's hard to reason fully about these statistics of mitochondrial concentration given that the petite phenotype is fundamentally affecting overall cell volume. can we have details on the cell size distribution in parallel with these results? to put it another way -- how does mitochondrial *amount* per cell change?

This is a good point. We report mostly on mitochondrial "concentrations" because we think this is what the cell actually cares about (mitochondrial activity in relationship to cytosolic activity). We have included additional graphs on mitochondrial amount as well as size distributions (Fig S2). The size distribution of the population does not change much in the first hours. Note that the "petite" phenotype refers to small colonies on growth medium with limited supply of a fermentable carbon source, not to smaller size of single cells.

3.11. l199 the mean in Fig S3C certainly does change -- it increases, clearly relative both to control and to its initial value. rather than sweeping this under the carpet we should look in more detail to understand it (a consequence of the increased skew of the distribution)?

This relates somewhat to the previous point. The increase in average *concentration* is not due to an increased amount in the population, but due to the fact that it is the small buds that get a very high amount of the mitochondria which "exaggerates" the asymmetric/heterogenous distribution.

3.12. para line 206 -- this doesn't make it clear whether your MMP signal is integrated over all mitochondria in the cell, or normalised by mitochondrial content? this matters quite a lot for the interpretation if the distributions of mitochondrial content are changing. reading on, this is even more important for para line 222.

Reading further on, there is an equation on l612 that gives a definition, but it doesn't really clarify (apologies if I'm misunderstanding).

For each cell, we basically calculate the relative mitochondrial enrichment of the MMP sensitive vs the MMP insensitive pre-sequence.

So, MMP= (total intensity of mitochondrial pre-Cox4 Neongreen/ total intensity of mitochondrial pre-Su9 Cardinal) / (total cytosolic pre-Cox4 Neongreen/ total cytosolic pre-Su9 Cardinal).

We calculate this value for each cell, but we do not have the optical resolution to calculate it for individual mitochondrial fragments.

Both constructs are driven by the same strong promoter, so transcription of the fluorophore should never limit the uptake. Also, in Figure 3D we compare control and depleted cells with similar total mitochondrial concentration, so the difference must be due to a different import of the two fluorophores, see also Fig EV4G. The calculated "MMP" value is of course only a crude proxy for the actual membrane potential in millivolts and we do not want to make any claims on absolute values or quantitative differences. But essentially what we are interested in is "mitochondrial health/activity" and we think the system is good at reporting this. See also 2.2.

3.13. l230 -- a point of personal interest -- low mito concentrations are connected to low "function" (MMP) and give extended division times -- this is interestingly exactly the model needed to reproduce observations in HeLa cells (https://journals.plos.org/ploscompbiol/article?id=10.1371/journal.pcbi.1002416). That model went on to predict several aspects of downstream cellular behaviour -- it would be very interesting to see how compatible that picture (parameterised using HeLa observations) is with yeast!

Thank you for pointing out your interesting paper, which we will include in our discussion. Another recent preprint about fission yeast (Chacko et al., 2025) also fits into this picture. Since you were kind enough to disclose your identity, we would be happy to discuss this further with you in person if we can maybe follow-up on this.

3.14. l239 "less mitochondria" -- a bit tricky but I'd say "fewer mitochondria" or "less mitochondrial content"

We rephrased to " less mitochondrial content".

3.15. Section l234
So here (and in Fig 4) the focus is on overall distributions of mitochondrial concentration in different cells (mother-to-be, mother, bud; gen 1, gen >1). But we've just seen that one effect of fzo1 is to broader the distribution of mitochondrial concentration across cells. Can't we look in more depth at the implications of this heterogeneity? For example in Fig 4F (which is cool) we look at the distribution of all fzo1 mothers-to-be, mothers, and buds. But this loses information about the provenance. For example, do mothers-to-be with extremely low mito concentrations just push everything to the bud, while mothers-to-be with high mito concentrations distribute things more evenly?

It would seem very easy and very interesting to somehow subset the distribution of mothers-to-be by concentration and see how different subsets behave

This is a good point. When analyzing the data, we pretty much plotted everything against everything and then chose the graphs that we think will best guide the reader through the story-line. We made additional EV plots where we show the starting concentrations/amounts of the mother in relationship to the resulting split ratio at the end of the cycle (Fig EV4C).

3.16. l285 -- experimental design -- do we know that Atp6 will continue to be a good proxy for functional mtDNA in the face of the perturbations provided by Fzo1 depletion? Especially if there is impact on the expression of mitoribosomes, the relationship between mtDNA and Atp6 may look rather different in the mutant?

We used the HI-NESS system and DAPI staining (Fig 5A, EV5) and made a more direct link to mtDNA/ nucleoid numbers, see 1.2.

3.17. l290 -- ruled out mitophagy. This message could be much clearer. Comparing Fig S5C and Fig 3A side-by-side is a needlessly difficult task -- put Fig 3A into Fig S5. Then we see that when mitophagy is compromised, the distribution of mitochondrial concentration has a lower median and much lower upper quartile than in the mitophagy-equipped Fzo1 mutant? What is going on here? For a paper motivated by disentangling coupled mechanisms, this should be made clearer!

Thanks for pointing this out. We have included the control in the corresponding figure (EV5C,D). We see that both, the mean and the median mitochondrial concentration and the Atp6 concentration are very similar when Fzo1 is depleted in the *atg32* deletion compared to a Control background. The second evidence that speaks against large-scale mitophagy is the proteomics data: On population level the dynamics of the respiratory chain proteins (Fig 2D, EV2I) are very different from those of other (nuclear encoded) mitochondrial proteins (now shown in Fig S1). Most mitochondrial proteins in the proteomics experiment stay constant in the first few hours, consistent with the imaging data showing that the mean mitochondrial content of the population does not change initially (Fig EV3D). This again highlights that it is the unequal distribution which is the problem and not massive degradation of mitochondria.

3.18. With the Atp6 signal, how do we know that fluorescence from different cells is comparable? Buds will be smaller than mother cells for example, potentially leading to less occlusion of the fluorescent signal by other content in the cytoplasm

This is of course a general problem that anyone faces doing quantitative fluorescence microscopy. From the technical side, we have done the best we could by taking a reasonable amount of z-slices and by choosing fluorophores that are in a range with little cellular background fluorescence (e.g. Neongreen is much better than GFP). From a practical standpoint, we are always comparing to the control, which is subject to the same technical limitations as the depleted cells and the cell sizes are very similar. So, even if we are systematically overestimating the Atp6 concentration in the bud by a few %, the difference to the control would still be qualitatively true. We therefore do not think that any of our conclusions are affected by this.

3.19. l343 -- maintenance of mtDNA -- here the point about l285 (is the Atp6-mtDNA relationship the same in the Fzo1 mutant) is particularly important, as we're directly tying findings about the protein product to implications about the mtDNA

The drop in nucleoid counts directly precedes the drop in Atp6. See above.

3.20. l367 -- on a first read this description of the model feels like lots of choices have been made without being fully justified. Why a log-normal distribution (when the fit to the data looks rather flawed); why the choice of 5 groups for nucleoid number (why not 3? or 8?); the process used for parameter fitting is very unclear (after reading the methods I think some of these values are read directly from the data, but the shapes of the distributions remain unexplained). l705 -- presumably the ratio was drawn from a log-normal distribution and then the corresponding nucleoid numbers were rounded to integers? the ratio itself wasn't rounded? (also l367) How were the log-normal distributions fitted to experiments (Figs. S7A,B)? Just by eye?

We chose a log-normal distribution because it was the 'simplest' distribution that captures the broad tail on the right of the distribution. We now clarified in the manuscript that the distributions were fitted to the experimental data using the Matlab function 'Lognormal'. While the log-normal distribution fits the WT data well, we agree with the reviewer that it does not perfectly fit the mutant. As an alternative, we now repeated the simulation using a Gamma distribution, which led qualitatively to the same results. Additionally, our model now allows the Atp6 daughter/mother ration to become 0 or Inf in all simulations, based on the observed frequencies in our data. This small modification led to an even faster drop in mtDNA, approaching the observed experimental dynamics. As discussed above, the mtDNA-dependent relative synthesis rate is necessary to obtain stable nucleoid distributions in the WT simulation. As we now clarify in the manuscript, our choice of 5 bins was a practical compromise that led to stable WT simulations while still ensuring a sufficient number of cells in each bin for WT and mutant to determine the values experimentally.

Yes, the reviewer is right. The nucleoid numbers were rounded, not the ratio. We clarified this in the revised version of the manuscript.

3.21. l711 by random selection -- just at random? ("selection" could be confusing)

Thanks for the comment, we revised accordingly.

Overall, it feels like the model may be too complicated for what it needs to show. Either (a) the model should show qualitatively that unequal inheritance and reduced production leads to rapid loss -- which a much simpler model, probably just involving a couple of lines of algebra, could show. Or (b) the model should quantitatively reproduce the particular numerical observations from the experiments -- it's not totally clear that it does this (do the cell-cycle-based decay timescales in Fig 7 correspond to the hour-based decay timescales in other plots, for example). At the moment the model is at a (b) level of detail but it's only clear that it's reporting the (a) level of results.

Aim of the modeling was to test whether unequal inheritance and/or reduced production can explain the rapid loss of mtDNA qualitatively, i.e. on a time scale that roughly matches that observed experimentally. A truly quantitative model as envisioned by the reviewer in (b) would require that we take into account cell-to-cell heterogeneity in growth and cell cycle progression, similar to what has been done by (Chandler-Brown et al., 2017) to understand cell size regulation. On top of such a cell cycle model, one would require detailed parameters for mitochondrial synthesis, etc., which is far beyond the scope of this study.

We therefore opted for a much simpler 'toy' model that only incorporates key features of the process. Our strategy when designing the model was to keep it as simple as possible while including sufficient detail to 1.) achieve nucleoid numbers that are stable in the WT condition on the time-scale of the simulation and 2.) capture the major differences between WT and mutant that we observed experimentally. For example, 1.) required that mtDNA replication was dependent on copy number. In the revised version of the manuscript, we now discuss this in more detail.

3.22. A lot of the discussion repeats the results; depending on editorial preferences some of this text could probably be pared back to focus on the literature connections and context.

We have modified the discussion a little bit. But we feel that the complexity of the data warrants a summary of the key findings before discussing them in a bigger context.

3.23. Data availability -- it looks like much of the data required to reproduce the results is not going to be made available. Images and proteomic data are promised, but the data associated with mitochondrial concentration and other features are not mentioned. For FAIR purposes all the data (including statistics from analysis of the images) should be published.

We maybe didn't phrase this clearly. All data will be made available. Where technically feasible, this will be directly accessible in a repository, otherwise by request to the corresponding author.

Our university provides an OMERO (Allan et al., 2012) server, which is aligned and in communication with the national initiative NFDI4Bioimage (https://nfdi4bioimage.de), where we have deposited many TB of raw images as well as all the intermediate steps such as segmentation masks, and the csv files with all the extracted data for each cell (including background corrections etc). As of now, the OMERO server is unfortunately only available by requesting a personal guest login from our bioinformatics facility, which will be granted independent of the authors. The proteomics data and the model are already fully accessible on PRIDE and GitHub, respectively. The raw western blot images with corresponding ponceau staining are included as additional supplementary material. The plasmids for the HI-NESS and the modified MitoLoc will be deposited on Addgene.

3.24 l660 -- can an overview of the EM protocol be given, to avoid having to buy the Mayer 2024 article?

The cited paper is open access. But we have also included more details in our method section.

References included here:

Allan, C., Burel, J. M., Moore, J., Blackburn, C., Linkert, M., Loynton, S., Macdonald, D., Moore, W. J., Neves, C., Patterson, A., Porter, M., Tarkowska, A., Loranger, B., Avondo, J., Lagerstedt, I., Lianas, L., Leo, S., Hands, K., Hay, R. T.,…Swedlow, J. R. (2012). OMERO: flexible, model-driven data management for experimental biology. *Nat Methods*, *9*(3), 245-253. https://doi.org/10.1038/nmeth.1896

Böckler, S., Chelius, X., Hock, N., Klecker, T., Wolter, M., Weiss, M., Braun, R. J., & Westermann, B. (2017). Fusion, fission, and transport control asymmetric inheritance of mitochondria and protein aggregates. *J Cell Biol*, *216*(8), 2481-2498. https://doi.org/10.1083/jcb.201611197

Chacko, L. A., Nakaoka, H., Morris, R., Marshall, W., & Ananthanarayanan, V. (2025). Mitochondrial function regulates cell growth kinetics to actively maintain mitochondrial homeostasis. *bioRxiv*. https://doi.org/10.1101/2025.03.31.646474

Chandler-Brown, D., Schmoller, K. M., Winetraub, Y., & Skotheim, J. M. (2017). The Adder Phenomenon Emerges from Independent Control of Pre- and Post-Start Phases of the Budding Yeast Cell Cycle. *Current Biology*, *27*(18), 2774-2783.e2773. https://doi.org/https://doi.org/10.1016/j.cub.2017.08.015

Christiano, R., Nagaraj, N., Frohlich, F., & Walther, T. C. (2014). Global proteome turnover analyses of the Yeasts S. cerevisiae and S. pombe. *Cell Rep*, *9*(5), 1959-1965. https://doi.org/10.1016/j.celrep.2014.10.065

Contamine, V., & Picard, M. (2000). Maintenance and integrity of the mitochondrial genome: a plethora of nuclear genes in the budding yeast. *Microbiol Mol Biol Rev*, *64*(2), 281-315. https://doi.org/10.1128/mmbr.64.2.281-315.2000

Deng, J., Swift, L., Zaman, M., Shahhosseini, F., Sharma, A., Bureik, D., Padovani, F., Benedikt, A., Jaiswal, A., Brideau, C., Grewal, S., Schmoller, K. M., Colarusso, P., & Shutt, T. E. (2025). A novel genetic fluorescent reporter to visualize mitochondrial nucleoids. *bioRxiv*, 2023.2010.2023.563667. https://doi.org/10.1101/2023.10.23.563667

Di Bartolomeo, F., Malina, C., Campbell, K., Mormino, M., Fuchs, J., Vorontsov, E., Gustafsson, C. M., & Nielsen, J. (2020). Absolute yeast mitochondrial proteome quantification reveals trade-off between biosynthesis and energy generation during diauxic shift. *Proc Natl Acad Sci U S A*, *117*(13), 7524-7535. https://doi.org/10.1073/pnas.1918216117

Ebert, A. C., Hepowit, N. L., Martinez, T. A., Vollmer, H., Singkhek, H. L., Frazier, K. D., Kantejeva, S. A., Patel, M. R., & MacGurn, J. A. (2025). Sphingolipid metabolism drives mitochondria remodeling during aging and oxidative stress. *bioRxiv*. https://doi.org/10.1101/2025.02.26.640157

Jakubke, C., Roussou, R., Maiser, A., Schug, C., Thoma, F., Bunk, R., Horl, D., Leonhardt, H., Walter, P., Klecker, T., & Osman, C. (2021). Cristae-dependent quality control of the mitochondrial genome. *Sci Adv*, *7*(36), eabi8886. https://doi.org/10.1126/sciadv.abi8886

Khan, A. H., Gu, X., Patel, R. J., Chuphal, P., Viana, M. P., Brown, A. I., Zid, B. M., & Tsuboi, T. (2024). Mitochondrial protein heterogeneity stems from the stochastic nature of co-translational protein targeting in cell senescence. *Nature Communications*, *15*(1), 8274. https://doi.org/10.1038/s41467-024-52183-y

Martin, J., Mahlke, K., & Pfanner, N. (1991). Role of an energized inner membrane in mitochondrial protein import. Delta psi drives the movement of presequences. *J Biol Chem*, *266*(27), 18051-18057.

Osman, C., Noriega, T. R., Okreglak, V., Fung, J. C., & Walter, P. (2015). Integrity of the yeast mitochondrial genome, but not its distribution and inheritance, relies on mitochondrial fission and fusion. *Proc Natl Acad Sci U S A*, *112*(9), E947-956. https://doi.org/10.1073/pnas.1501737112

Perić, M., Dib, P. B., Dennerlein, S., Musa, M., Rudan, M., Lovrić, A., Nikolić, A., Šarić, A., Sobočanec, S., Mačak, Ž., Raimundo, N., & Kriško, A. (2016). Crosstalk between cellular compartments protects against proteotoxicity and extends lifespan. *Scientific Reports*, *6*(1), 28751. https://doi.org/10.1038/srep28751

Roussou, R., Metzler, D., Padovani, F., Thoma, F., Schwarz, R., Shraiman, B., Schmoller, K. M., & Osman, C. (2024). Real-time assessment of mitochondrial DNA heteroplasmy dynamics at the single-cell level. *The EMBO Journal*, *43*(22), 5340-5359-5359. https://doi.org/https://doi.org/10.1038/s44318-024-00183-5

Seel, A., Padovani, F., Mayer, M., Finster, A., Bureik, D., Thoma, F., Osman, C., Klecker, T., & Schmoller, K. M. (2023). Regulation with cell size ensures mitochondrial DNA homeostasis during cell growth. *Nat Struct Mol Biol*, *30*(10), 1549-1560. https://doi.org/10.1038/s41594-023-01091-8

Vowinckel, J., Hartl, J., Butler, R., & Ralser, M. (2015). MitoLoc: A method for the simultaneous quantification of mitochondrial network morphology and membrane potential in single cells. *Mitochondrion*, *24*, 77-86. https://doi.org/10.1016/j.mito.2015.07.001

Wisniewski, B. T., Casler, J. C., & Lackner, L. L. (2024). Significantly reduced, but balanced, rates of mitochondrial fission and fusion are sufficient to maintain the integrity of yeast mitochondrial DNA. *Molecular Biology of the Cell*, *35*(12), br25. https://doi.org/10.1091/mbc.E24-07-0306

Dear Prof. Ewald

Thank you for the submission of your revised manuscript to EMBO reports. We have now received the full set of referee reports that is copied below.

As you will see, all referees are very positive about the study and request only minor changes to clarify text and figures. Referee 1 suggests adding informative titles to the EV figures, which is something we actually also request in the figure legend. Each legend needs an informative title. The suggestion of adding a summary text for each figure panel is something we indeed do not have, but if the text is not too long, maybe just a short sentence describing what we are looking at, would be fine. Maybe best to discuss this further via e-mail or personal discussion.

From the editorial side, there are also a few things that we need before we can proceed with the official acceptance of your study.

- Please remove the main and EV figures from the manuscript. The main and EV figure legends should stay at the end. Please update the nomenclature of the EV Figure Legends (currently it says Extened view Figure but it shoule be Figure EV#).

- The manuscript sections should be in the following order: Title page - Abstract & Keywords - Introduction - Results - Discussion - Methods - Data Availability - Acknowledgments - Disclosure Statement & Competing Interests - References - Figure Legends - (Main Tables with legends if applicable) - Expanded View Figure Legends.

- Please add a 'Disclosure and competing interests statement'. For more information see https://link.springer.com/journal/44319/submission-guidelines#conflictsofinterest

- Please provide the corresponding author's email on the title page.

- Regarding the Author Contributions, we now use CRediT to specify the contributions of each author in the journal submission system. CRediT replaces the author contribution section, which therefore needs to be removed from the manuscript text. You can use the free text box in our system if you wish to provide more detailed descriptions.

- References: et al needs to be used after 10 author names; DOIs should only be used for preprints and datasets that have not been published yet.

- The funding information needs to be part of the Acknowledgments section, the separate 'Funding' section is not needed. The Helmholtz Association is missing as a separate funder in the online submission system. Note that the funding information in the manuscript and the system needs to be congruent.

- Appendix file: We need a table of content with page numbers for each item. The nomenclature of the items need correction to Appendix Figure S1, etc. Appendix Table S1, Appendix References etc. "Supplementary Material for" should be updated to "Appendix for XXX.
- Please update the table callouts in the Reagents and Tools table accordingly.

- The references in the Reagent and Tools table should be added to the main reference section, since the table is eventually part of the manuscript.

- Callouts for Appendix Figure S2, Appendix FIgure S3, Appendix Table S7, Appendix Table S8 are missing. Please add these in the text where appropriate.

- You mention "Supplementary information" on page 10, line 300. Given that we do not use the term "Supplementary" please specify where this information can be found using the exact item/nomenclature.

- Supplementary Figure 2: please add information on the number of samples and define the box plot.

- The movie legends should be removed from the manuscript; each should be provided in a text file and then zipped up together with its movie so that we have 5 zip folders uploaded separately; the correct nomenclature in all places should be Movie EV1, etc.

- Source Data: please provide the numerical data for Fig. 1B, 2A, C, E, F, H.

- The README. Text file for the EM source data needs a callout update. You refer to ext. fig. 2J, which I assume is Figure EV2J?

- Please provide the specific URL for PXD063340 in the data availability statement.

* Please address the following comments in the Figure Legends:
- Please provide the exact p values in the legends of figures 2C, 3D, 4C, D, I; EV3 I, EV4 E. The values can also be provided in the figure panel itself.
- Please define the box plots in terms of minima, maxima, centre, bounds of box and whiskers, and percentile in the legends of figures 3D, 4A, C, D, E, F, G, H, I; 5C, 6C, 7G, EV2 H, EV3 C, F, I; EV4 A-F; EV5 E, F; EV6 D, EV7 B, C; S1 B
- Please define the box plots in terms of minima, maxima, centre in the legend of figure 7C
- Please define the box plots in terms of minima, maxima, centre, bounds of box and whiskers in the legend of figure EV1 C
- Please note that information related to n is missing in the legends of figures 7G, EV1 E, EV2 B, C, G; EV2 H, EV5 F, S1 B
- Please note that the error bars are not defined in the legends of figures EV1 E, EV2 B, G; EV8 C, D, E, F
- Please note that the measure of center for the error bars needs to be defined in the legends of figures EV2 A, D, I

- Please show the individual datapoints in the addition to the mean and error bar in all quantification graphs.

- Please define the size of the scale bar only in the legends, not the figure.

- Citations to preprints must be labeled with the tag [PREPRINT] at the end of the reference and with preprint: in the text. E.g., the reference to Chacko et al, 2025 would look like this: (preprint: Chacko et al, 2025).
This applies to Chacko et al, 2025, Deng et al, 2025, Padovani et al, 2024, Ray et al, 2025, unless these have meanwhile been published in a journal.

As a standard procedure, we modify title and abstracts. Please find a few title suggestions below, as well as a slightly modified abstract.

- Finally, EMBO Reports papers are accompanied online by
A) a short (1-2 sentences) summary of the findings and their significance,
B) 2-3 bullet points highlighting key results and
C) a schematic summary figure that provides a sketch of the major findings (not a data image).
Please provide the summary figure as a separate file in PNG or JPG format at a size of 550x300-600 pixels (width x height). Please note that the size is rather small and that text needs to be readable at the final size. Please send us this information along with the revised manuscript.

We look forward to seeing a final version of your manuscript as soon as possible.

With kind regards,

Martina Rembold, PhD
Senior Editor
EMBO reports

================

Referee #1:

Since my previous review via Commons, the authors have addressed many of my main concerns (and, to my eyes, those of the other reviewers): notably Atp6 as an mtDNA proxy (now addressed by counting mtDNAs), unpacking some aspects of mitochondrial amount vs concentration, addressing possible mitophagy, and simplifying the mathematical model to an appropriate level of detail for the phenomenology it needs to describe. I think all these improvements have made the story tighter and more robust.

I have a few small comments below, mainly phrasing and styling issues. My only bigger comment is that there is now -- even more than before -- a tremendous amount of display item content for the reader to absorb. I counted 95 figures, many of which are themselves split into subpanels. This is in part a response to all the requested additional information from the review process -- but is still a lot! At the moment the split into SI (EV) figures and main text figures is quite sensible, but I wonder if the reader's hand can be held even more through this volume of information? Two immediate things that come to mind are:
- Bold first-sentence titles for EV figure captions (like the main text ones) so the main takehome of each is clear
- Similar first-sentence subtitles for each subpanel of main text figures, giving the scientific message before the technical and statistical details. Here we come up against journal style guides, but I think e.g. a short italic scientific message for each panel before the technical detail would help tell the story more easily in a figure-heavy paper.

IJ

l37 -- citation to human mtDNA reduction from impaired fusion?
l229 -- for concreteness, integrated into the *nuclear* genome (otherwise something astonishing has happened!)
l257 -- less -> fewer mitochondria
l265 unnecessary period before figure link
l443 -- ratio rounded to integers?? Ah -- the methods makes this clearer -- it is the final number of nucleoids that (reasonably) gets rounded. This phrasing makes it sounds like the fraction gets rounded!
I still think that the first part of the discussion spends a lot of time repeating the results -- a more compact summary (one paragraph rather than several) would make the reading smoother
l856 -- *The* aim of our simulation
l863 -- citation for Matlab. Is the code transferable to a free alternative e.g. Octave, or do researchers need to buy Matlab to reproduce the modelling?

Since my previous review, there's another detailed paper from the Osman group on mtDNA and proteome dynamics -- influenced by selection and physiology -- that it's worth engaging with https://journals.plos.org/plosgenetics/article?id=10.1371/journal.pgen.1011836

EV Fig 1E -- "no major impact" -- but one impact is nearly 20% and the other around 10%. That's at least one or two extra nucleoids per cell (given 16 normally). Isn't this a pretty dramatic impact? Given the scale of some of the other effect sizes in the results?

Styling point -- in several figures the y-axis label is very lengthy and single-line (like Fig 3A). Can you split it into two or more lines so we don't have to e.g. abbreviate "normalised"?

Fig 3 -- to my eyes it would help to have a visualisation of the variability itself as a response variable -- it's hard to visually compare the IQR over time with these shaded regions. Could we have an inset where simply the IQR itself is plotted over time? I wonder if this would also help for the elements of Fig 4, where much of the focus is on variability. The difficulty with representing this as boxplots is that people will be used to boxplots that reflect a combination of biological and technical variability -- so a wide box is instinctively intepreted as "noisy data". Plotting the IQR explicitly would highlight that it is the quantity of central scientific interest. But just a thought.

Fig 8 -- necessary and sufficient -- this is a strong claim. Certainly the model shows that it is sufficient. But to show it is necessary would require comparing all possible other explanations of delta-fzo1 phenotype, which seems an insurmountable task. Isn't "sufficient" sufficiently interesting?

Styling point -- in some of the figures, especially the extended views, there are grey traces remaining where different panels have been cut and pasted together (EV Fig 5F for example) -- can these be removed?

Referee #2:

The authors have extensinvely responded all my previous criticisms and those of the othe other reviewers. In my opinion, this manuscript is suitable for publication in EMBO Reports.

Referee #3:

This manuscript by Dengler et al is a thorough and carefully-executed study of the mechanisms that lead to the defects seen upon loss of mitochondrial fusion in a fzo1-depleted yeast strain. In this revised version, the authors have satisfactorily addressed all my concerns, and added an elegant recovery experiment (Fig. 7) to better elucidate the nature of the observed phenotypes. Through analysis of tens of thousands of single cells across their cell cycle and across generations, the authors uncover two contributing mechanisms that together are sufficient and necessary to explain the loss of mitochondria and mtDNA upon fzo1-depletion: the uneven inheritance of these components to daughter cells, as well as the loss of a compensatory mechanism that should upregulate mtDNA expression and biogenesis upon mt dysfunction. These findings are of interest for our understanding of disorders that involve defects in mitochondrial fusion. I congratulate the authors for an interesting and high-quality study.

==================

Suggested title(s) and abstract:

Loss of mitochondrial fusion results in imbalanced inheritance of mitochondria and progressive mtDNA loss

In the absence of mitochondrial fusion unequal mitochondria segregation drives mtDNA loss

Loss of mitochondrial fusion induces asymmetric inheritance of mitochondria and loss of mtDNA

Asymmetric inheritance of mitochondria underlies mtDNA loss in fusion-deficient cells

Mitochondrial biogenesis and inheritance must be tightly coordinated with cell division to maintain mitochondrial function and cell survival. The dynamics of the mitochondrial network, including fusion and fission, are essential for mitochondrial inheritance and quality control. In budding yeast, simultaneous inhibition of both processes compromises mitochondrial DNA (mtDNA) integrity, increasing the frequency of petite cells. Loss of fusion alone completely eliminates mtDNA. Although this has been known for decades, why mtDNA is lost remained unclear. Here, we examine the effects of impaired mitochondrial fusion by depleting the mitofusin Fzo1. By analyzing over thirty thousand single cells across their cell cycles, we show that Fzo1-depletion induces rapid mitochondrial fragmentation and loss of membrane potential, followed by progressive declines in mtDNA content and growth rate. During division, Fzo1-depleted daughters inherit disproportionately large mitochondrial amounts, leaving mothers with too little. This imbalance, combined with an inability to upregulate compensatory mtDNA synthesis, drives rapid mtDNA loss. Our results reveal how fusion defects cause mtDNA loss and mitochondrial dysfunction, which might have implications for diseases linked to impaired fusion.

Referee #1:

Since my previous review via Commons, the authors have addressed many of my main concerns (and, to my eyes, those of the other reviewers): notably Atp6 as an mtDNA proxy (now addressed by counting mtDNAs), unpacking some aspects of mitochondrial amount vs concentration, addressing possible mitophagy, and simplifying the mathematical model to an appropriate level of detail for the phenomenology it needs to describe. I think all these improvements have made the story tighter and more robust.

I have a few small comments below, mainly phrasing and styling issues. My only bigger comment is that there is now -- even more than before -- a tremendous amount of display item content for the reader to absorb. I counted 95 figures, many of which are themselves split into subpanels. This is in part a response to all the requested additional information from the review process -- but is still a lot! At the moment the split into SI (EV) figures and main text figures is quite sensible, but I wonder if the reader's hand can be held even more through this volume of information? Two immediate things that come to mind are:
- Bold first-sentence titles for EV figure captions (like the main text ones) so the main takehome of each is clear
- Similar first-sentence subtitles for each subpanel of main text figures, giving the scientific message before the technical and statistical details. Here we come up against journal style guides, but I think e.g. a short italic scientific message for each panel before the technical detail would help tell the story more easily in a figure-heavy paper.

IJ

l37 -- citation to human mtDNA reduction from impaired fusion?
l229 -- for concreteness, integrated into the *nuclear* genome (otherwise something astonishing has happened!)
l257 -- less -> fewer mitochondria
l265 unnecessary period before figure link
l443 -- ratio rounded to integers?? Ah -- the methods makes this clearer -- it is the final number of nucleoids that (reasonably) gets rounded. This phrasing makes it sounds like the fraction gets rounded!
I still think that the first part of the discussion spends a lot of time repeating the results -- a more compact summary (one paragraph rather than several) would make the reading smoother
l856 -- *The* aim of our simulation
l863 -- citation for Matlab. Is the code transferable to a free alternative e.g. Octave, or do researchers need to buy Matlab to reproduce the modelling?

Since my previous review, there's another detailed paper from the Osman group on mtDNA and proteome dynamics -- influenced by selection and physiology -- that it's worth engaging with https://journals.plos.org/plosgenetics/article?id=10.1371/journal.pgen.1011836

EV Fig 1E -- "no major impact" -- but one impact is nearly 20% and the other around 10%. That's at least one or two extra nucleoids per cell (given 16 normally). Isn't this a pretty dramatic impact? Given the scale of some of the other effect sizes in the

results?

Styling point -- in several figures the y-axis label is very lengthy and single-line (like Fig 3A). Can you split it into two or more lines so we don't have to e.g. abbreviate "normalised"?

Fig 3 -- to my eyes it would help to have a visualisation of the variability itself as a response variable -- it's hard to visually compare the IQR over time with these shaded regions. Could we have an inset where simply the IQR itself is plotted over time? I wonder if this would also help for the elements of Fig 4, where much of the focus is on variability. The difficulty with representing this as boxplots is that people will be used to boxplots that reflect a combination of biological and technical variability -- so a wide box is instinctively intepreted as "noisy data". Plotting the IQR explicitly would highlight that it is the quantity of central scientific interest. But just a thought.

Fig 8 -- necessary and sufficient -- this is a strong claim. Certainly the model shows that it is sufficient. But to show it is necessary would require comparing all possible other explanations of delta-fzo1 phenotype, which seems an insurmountable task. Isn't "sufficient" sufficiently interesting?

Styling point -- in some of the figures, especially the extended views, there are grey traces remaining where different panels have been cut and pasted together (EV Fig 5F for example) -- can these be removed?

Referee #2:

The authors have extensinvely responded all my previous criticisms and those of the othe other reviewers. In my opinion, this manuscript is suitable for publication in EMBO Reports.

Referee #3:

This manuscript by Dengler et al is a thorough and carefully-executed study of the mechanisms that lead to the defects seen upon loss of mitochondrial fusion in a fzo1-depleted yeast strain. In this revised version, the authors have satisfactorily addressed all my concerns, and added an elegant recovery experiment (Fig. 7) to better elucidate the nature of the observed phenotypes. Through analysis of tens of thousands of single cells across their cell cycle and across generations, the authors uncover two contributing mechanisms that together are sufficient and necessary to explain the loss of mitochondria and mtDNA upon fzo1-depletion: the uneven inheritance of these components to daughter cells, as well as the loss of a compensatory mechanism that should upregulate mtDNA expression and biogenesis upon mt dysfunction. These findings are of interest for our understanding of disorders that involve defects in mitochondrial fusion. I congratulate the authors for an interesting and high-quality study.

***

Rev_Com_number: RC-2025-03153
New_manu_number: EMBOR-2025-62929V2
Corr_author: Ewald
Title: Unbalanced mitochondrial segregation triggers loss of mtDNA in the absence of mitochondrial fusion

Response to 2nd round reviews for

In the absence of mitochondrial fusion unequal segregation of mitochondria drives mtDNA loss

By Dengler et al

Referee #1:
Since my previous review via Commons, the authors have addressed many of my main concerns (and, to my eyes, those of the other reviewers): notably Atp6 as an mtDNA proxy (now addressed by counting mtDNAs), unpacking some aspects of mitochondrial amount vs concentration, addressing possible mitophagy, and simplifying the mathematical model to an appropriate level of detail for the phenomenology it needs to describe. I think all these improvements have made the story tighter and more robust.

We appreciate the positive feedback.

I have a few small comments below, mainly phrasing and styling issues. My only bigger comment is that there is now -- even more than before -- a tremendous amount of display item content for the reader to absorb. I counted 95 figures, many of which are themselves split into subpanels. This is in part a response to all the requested additional information from the review process -- but is still a lot! At the moment the split into SI (EV) figures and main text figures is quite sensible, but I wonder if the reader's hand can be held even more through this volume of information? Two immediate things that come to mind are:

- Bold first-sentence titles for EV figure captions (like the main text ones) so the main take home of each is clear

Done.

- Similar first-sentence subtitles for each subpanel of main text figures, giving the scientific message before the technical and statistical details. Here we come up against journal style guides, but I think e.g. a short italic scientific message for each panel before the technical detail would help tell the story more easily in a figure-heavy paper.

This is indeed not journal style. But we have gone through all figure legends and tried to edit for clarity.

l37 -- citation to human mtDNA reduction from impaired fusion?

Fixed, this got lost somewhere during editing.

l229 -- for concreteness, integrated into the *nuclear* genome (otherwise something astonishing has happened!)

We changed this.

l257 -- less -> fewer mitochondria

Done

l265 unnecessary period before figure link

Done

l443 -- ratio rounded to integers?? Ah -- the methods makes this clearer -- it is the final number of nucleoids that (reasonably) gets rounded. This phrasing makes it sounds like the fraction gets rounded!

This phrasing was indeed confusing. Since the rounding is only a technical detail, we removed this completely, since it is explained step by step in the methods.

I still think that the first part of the discussion spends a lot of time repeating the results -- a more compact summary (one paragraph rather than several) would make the reading smoother

We agree that the discussion is rather long. However, we think it is useful to have a summary of the key biological findings without the technical details described in the results.

l856 -- *The* aim of our simulation

Done.

l863 -- citation for Matlab. Is the code transferable to a free alternative e.g. Octave, or do researchers need to buy Matlab to reproduce the modelling?

We used Matlab since this is our standard tool for simulations. However, the .m files on Github can be opened within Github or as text files. So these can be easily converted to other programming languages.

Since my previous review, there's another detailed paper from the Osman group on mtDNA and proteome dynamics -- influenced by selection and physiology -- that it's worth engaging with https://journals.plos.org/plosgenetics/article?id=10.1371/journal.pgen.1011836

Thoma et al is a beautiful paper that we are well aware of, we note that two of our co-authors are also co-authors on this. However, the main point of this publication is heteroplasmia and mitochondrial quality control. We do not see how to integrate this into our discussion without an entirely new paragraph devoted to this.

EV Fig 1E -- "no major impact" -- but one impact is nearly 20% and the other around 10%. That's at least one or two extra nucleoids per cell (given 16 normally). Isn't this a pretty dramatic impact? Given the scale of some of the other effect sizes in the results?

We have replaced the bar plot with individual data points and error bars. It now becomes very apparent that 10-20% difference is well within the noise. Additionally, the grey control (Tir + aTC+5-PhIAA), which is essentially the combination of both of the orange treatments, is very close to 1.

Regarding effect size, we note that this is the control mainly for Figure 2A, where the effect size is going from 100% to 0% mtDNA content, so much bigger than the noise in the control. Seemingly smaller effect sizes such as the MMP measurement are based on dozens to hundreds of individual data points.

Styling point -- in several figures the y-axis label is very lengthy and single-line (like Fig 3A). Can you split it into two or more lines so we don't have to e.g. abbreviate "normalised"?
We tried to avoid splitting the axis labelling wherever possible since we find this harder to read.

Fig 3 -- to my eyes it would help to have a visualisation of the variability itself as a response variable -- it's hard to visually compare the IQR over time with these shaded regions. Could we have an inset where simply the IQR itself is plotted over time?
I wonder if this would also help for the elements of Fig 4, where much of the focus is on variability. The difficulty with representing this as boxplots is that people will be used to boxplots that reflect a combination of biological and technical variability -- so a wide box is instinctively intepreted as "noisy data". Plotting the IQR explicitly would highlight that it is the quantity of central scientific interest. But just a thought.

As the reviewer comments, the figures are already very busy. We have thus included a plot of only the interquartile range (delta between 75th and 25th percentile for each time point) for Figure 3A in Appendix Figure 2A rather than including it in the main figure. For Figure 4 we think it is important to not only show the variability, but to highlight that the distribution across the population of cells is so wide that the lower end reaches 0, we would thus like to keep the figure as is.

Fig 8 -- necessary and sufficient -- this is a strong claim. Certainly the model shows that it is sufficient. But to show it is necessary would require comparing all possible other explanations of delta-fzo1 phenotype, which seems an insurmountable task. Isn't "sufficient" sufficiently interesting?

The reviewer is correct, we have removed the necessary.

Styling point -- in some of the figures, especially the extended views, there are grey traces remaining where different panels have been cut and pasted together (EV Fig 5F for example) -- can these be removed?
We have addressed this with the editor, it doesn't seem to be present in the original files.

Referee #2:

The authors have extensinvely responded all my previous criticisms and those of the othe other reviewers. In my opinion, this manuscript is suitable for publication in EMBO Reports.
We thank the reviewer for his/her support.

Referee #3:

This manuscript by Dengler et al is a thorough and carefully-executed study of the mechanisms

that lead to the defects seen upon loss of mitochondrial fusion in a fzo1-depleted yeast strain. In this revised version, the authors have satisfactorily addressed all my concerns, and added an elegant recovery experiment (Fig. 7) to better elucidate the nature of the observed phenotypes. Through analysis of tens of thousands of single cells across their cell cycle and across generations, the authors uncover two contributing mechanisms that together are sufficient and necessary to explain the loss of mitochondria and mtDNA upon fzo1-depletion: the uneven inheritance of these components to daughter cells, as well as the loss of a compensatory mechanism that should upregulate mtDNA expression and biogenesis upon mt dysfunction. These findings are of interest for our understanding of disorders that involve defects in mitochondrial fusion. I congratulate the authors for an interesting and high-quality study.

We thank the reviewer for his support and the compliment!

Prof. Jennifer Ewald
University of Tuebingen
Institute of Cell Biology
Tuebingen 72076
Germany

Dear Jennifer,

I am very pleased to accept your manuscript for publication in the next available issue of EMBO reports. Thank you for your contribution to our journal.

Your manuscript will be processed for publication by EMBO Press. It will be copy edited and you will receive page proofs prior to publication. Please note that you will be contacted by Springer Nature Author Services to complete licensing and payment information.

You may qualify for financial assistance for your publication charges - either via a Springer Nature fully open access agreement or an EMBO initiative. Check your eligibility: https://link.springer.com/journal/44319/how-to-publish-with-us

Should you be planning a Press Release on your article, please get in contact with embo_production@springernature.com as early as possible in order to coordinate publication and release dates.

If you have any questions, please do not hesitate to contact the Editorial Office. Thank you for your contribution to EMBO Reports.

Kind regards,

Martina

Martina Rembold, PhD
Senior Editor
EMBO reports

>>> Please note that it is EMBO Reports policy for the transcript of the editorial process (containing referee reports and your response letter) to be published as an online supplement to each paper. If you do NOT want this, you will need to inform the Editorial Office via email immediately. More information is available here: https://link.springer.com/partners/embo-press/editorial-policies#Peer%20review

EMBO Press Author Checklist

Journal Submitted to: Jennifer C. Ewald	
Journal Submitted to: EMBO Reports	
Manuscript Number: EMBOR-2025-62929V1-T	

USEFUL LINKS FOR COMPLETING THIS FORM
The EMBO Journal - Author Guidelines
EMBO Reports - Author Guidelines
Molecular Systems Biology - Author Guidelines
EMBO Molecular Medicine - Author Guidelines

Reporting Checklist for Life Science Articles (updated January
This checklist is adapted from Materials Design Analysis Reporting (MDAR) Checklist for Authors. MDAR establishes a minimum set of requirements in transparent reporting in the life sciences (see Statement of Task: 10.31222/osf.io/9sm4x). Please follow the journal's guidelines in preparing your
Please note that a copy of this checklist will be published alongside your article.

Abridged guidelines for figures
1. Data
The data shown in figures should satisfy the following conditions:
- the data were obtained and processed according to the field's best practice and are presented to reflect the results of the experiments in an accurate and unbiased manner.
- ideally, figure panels should include only measurements that are directly comparable to each other and obtained with the same assay.
- plots include clearly labeled error bars for independent experiments and sample sizes. Unless justified, error bars should not be shown for technical
- if n<5, the individual data points from each experiment should be plotted. Any statistical test employed should be justified.
- Source Data should be included to report the data underlying figures according to the guidelines set out in the authorship guidelines on Data

2. Captions
Each figure caption should contain the following information, for each panel where they are relevant:
- a specification of the experimental system investigated (eg cell line, species name).
- the assay(s) and method(s) used to carry out the reported observations and measurements.
- an explicit mention of the biological and chemical entity(ies) that are being measured.
- an explicit mention of the biological and chemical entity(ies) that are altered/varied/perturbed in a controlled manner.
- the exact sample size (n) for each experimental group/condition, given as a number, not a range;
- a description of the sample collection allowing the reader to understand whether the samples represent technical or biological replicates (including how many animals, litters, cultures, etc.).
- a statement of how many times the experiment shown was independently replicated in the laboratory.
- definitions of statistical methods and measures:
  - common tests, such as t-test (please specify whether paired vs. unpaired), simple χ2 tests, Wilcoxon and Mann-Whitney tests, can be unambiguously identified by name only, but more complex techniques should be described in the methods section;
  - are tests one-sided or two-sided?
  - are there adjustments for multiple comparisons?
  - exact statistical test results, e.g., P values = x but not P values < x;
  - definition of 'center values' as median or average;
  - definition of error bars as s.d. or s.e.m.

Please complete ALL of the questions below.
Select "Not Applicable" only when the requested information is not relevant for your study.

Materials

Newly Created Materials	Information included in the manuscript?	In which section is the information available? (Reagents and Tools Table, Materials and Methods, Figures, Data Availability Section)
New materials and reagents need to be available; do any restrictions apply?	Yes	No Restrictions Apply. All strains and plasmids are listed in the Appendix and can be obtained from the corresponding author.

Antibodies	Information included in the manuscript?	In which section is the information available? (Reagents and Tools Table, Materials and Methods, Figures, Data Availability Section)
For **antibodies** provide the following information:<br>- Commercial antibodies: RRID (if possible) or supplier name, catalogue number and or/clone number<br>- Non-commercial: RRID or citation	Yes	The informaiton is included both in the Method section and in the Reagents and Tools Table.

DNA and RNA sequences	Information included in the manuscript?	In which section is the information available? (Reagents and Tools Table, Materials and Methods, Figures, Data Availability Section)
**Short novel DNA or RNA including primers, probes**: provide the sequences.	Yes	Sequences of all primers are listed in a Supplementary Table

Cell materials	Information included in the manuscript?	In which section is the information available? (Reagents and Tools Table, Materials and Methods, Figures, Data Availability Section)
**Cell lines:** Provide species information, strain. Provide accession number in repository **OR** supplier name, catalog number, clone number, and/**OR** RRID.	Not Applicable	
**Primary cultures:** Provide species, strain, sex of origin, genetic modification status.	Not Applicable	
Report if the cell lines were recently **authenticated** (e.g., by STR profiling) and tested for mycoplasma contamination.	Not Applicable	

Experimental animals	Information included in the manuscript?	In which section is the information available? (Reagents and Tools Table, Materials and Methods, Figures, Data Availability Section)
**Laboratory animals or Model organisms:** Provide species, strain, sex, age, genetic modification status. Provide accession number in repository **OR** supplier name, catalog number, clone number, **OR** RRID.	Not Applicable	
**Animal observed in or captured from the field:** Provide species, sex, and age where possible.	Not Applicable	
Please detail **housing and husbandry conditions**.	Not Applicable	

Plants and microbes	Information included in the manuscript?	In which section is the information available? (Reagents and Tools Table, Materials and Methods, Figures, Data Availability Section)
**Plants:** provide species and strain, ecotype and cultivar where relevant, unique accession number if available, and source (including location for collected wild specimens).	Not Applicable	
**Microbes:** provide species and strain, unique accession number if available, and source.	Yes	We created various S.cerevisiae strains. These are listed with full genotype in a supplementary table.

Human research participants	Information included in the manuscript?	In which section is the information available? (Reagents and Tools Table, Materials and Methods, Figures, Data Availability Section)
If collected and within the bounds of privacy constraints report on age, sex and gender or ethnicity for all study participants.	Not Applicable	

Core facilities	Information included in the manuscript?	In which section is the information available? (Reagents and Tools Table, Materials and Methods, Figures, Data Availability Section)
If your work benefited from core facilities, was their service mentioned in the acknowledgments section?	Not Applicable	

Design

Study protocol	Information included in the manuscript?	In which section is the information available? (Reagents and Tools Table, Materials and Methods, Figures, Data Availability Section)
If study protocol has been **pre-registered, provide DOI in the manuscript**. For clinical trials, provide the trial registration number **OR** cite DOI.	Not Applicable	
Report the **clinical trial registration number** (at ClinicalTrials.gov or equivalent), where applicable.	Not Applicable	

Laboratory protocol	Information included in the manuscript?	In which section is the information available? (Reagents and Tools Table, Materials and Methods, Figures, Data Availability Section)
Provide DOI OR other citation details if **external detailed step-by-step protocols** are available.	Not Applicable	

Experimental study design and statistics	Information included in the manuscript?	In which section is the information available? (Reagents and Tools Table, Materials and Methods, Figures, Data Availability Section)
Include a statement about **sample size** estimate even if no statistical methods were used.	Yes	We include detailed information in the Figure Legends.
Were any steps taken to minimize the effects of subjective bias when allocating animals/samples to treatment (e.g. **randomization procedure**)? If yes, have they been described?	Not Applicable	
Include a statement about **blinding** even if no blinding was done.	Not Applicable	
Describe **inclusion/exclusion criteria** if samples or animals were excluded from the analysis. Were the criteria pre-established?<br><br>If sample or data points were omitted from analysis, report if this was due to attrition or intentional exclusion and provide justification.	Not Applicable	
For every figure, are **statistical tests** justified as appropriate? Do the data meet the assumptions of the tests (e.g., normal distribution)? Describe any methods used to assess it. Is there an estimate of variation within each group of data? Is the variance similar between the groups that are being statistically compared?	Yes	Details of statistical test including assumptions/test parameters are included in the figure legends.

Sample definition and in-laboratory replication	Information included in the manuscript?	In which section is the information available? (Reagents and Tools Table, Materials and Methods, Figures, Data Availability Section)
In the figure legends: state number of times the experiment was **replicated** in laboratory.	Yes	Is stated in the figure legends
In the figure legends: define whether data describe **technical or biological replicates**.	Yes	Is stated in the figure legends

Ethics

Ethics	Information included in the manuscript?	In which section is the information available? (Reagents and Tools Table, Materials and Methods, Figures, Data Availability Section)
Studies involving **human participants**: State details of **authority granting ethics approval** (IRB or equivalent committee(s), provide reference number for approval.	Not Applicable	
Studies involving **human participants**: Include a statement confirming that **informed consent** was obtained from all subjects and that the experiments conformed to the principles set out in the WMA Declaration of Helsinki and the Department of Health and Human Services Belmont Report.	Not Applicable	
Studies involving **human participants:** For publication of **patient photos**, include a statement confirming that consent to publish was obtained.	Not Applicable	
Studies involving experimental **animals**: State details of **authority granting ethics approval** (IRB or equivalent committee(s), provide reference number for approval. Include a statement of compliance with ethical regulations.	Not Applicable	
Studies involving **specimen and field samples:** State if relevant **permits** obtained, provide details of authority approving study; if none were required, explain why.	Not Applicable	

Dual Use Research of Concern (DURC)	Information included in the manuscript?	In which section is the information available? (Reagents and Tools Table, Materials and Methods, Figures, Data Availability Section)
Could your study fall under dual use research restrictions? Please check biosecurity documents and list of **select agents and toxins** (CDC): https://www.selectagents.gov/sat/list.htm	Not Applicable	
If you used a select agent, is the security level of the lab appropriate and reported in the manuscript?	Not Applicable	
If a study is subject to dual use research of concern regulations, is the name of the **authority granting approval and reference number** for the regulatory approval provided in the manuscript?	Not Applicable	

Reporting

The MDAR framework recommends adoption of discipline-specific guidelines, established and endorsed through community initiatives. Journals have their own policy about requiring specific guidelines and recommendations to complement MDAR.

Adherence to community standards	Information included in the manuscript?	In which section is the information available? (Reagents and Tools Table, Materials and Methods, Figures, Data Availability Section)
State if relevant guidelines or checklists (e.g., **ICMJE, MIBBI, ARRIVE, PRISMA**) have been followed or provided.	Not Applicable	
For **tumor marker prognostic studies,** we recommend that you follow the **REMARK** reporting guidelines (see link list at top right). See author guidelines, under 'Reporting Guidelines'. Please confirm you have followed these guidelines.	Not Applicable	
For **phase II and III randomized controlled trials**, please refer to the **CONSORT** flow diagram (see link list at top right) and submit the CONSORT checklist (see link list at top right) with your submission. See author guidelines, under 'Reporting Guidelines'. Please confirm you have submitted this list.	Not Applicable	

Data Availability

Data availability	Information included in the manuscript?	In which section is the information available? (Reagents and Tools Table, Materials and Methods, Figures, Data Availability Section)
Have **primary datasets** been deposited according to the journal's guidelines (see 'Data Deposition' section) and the respective accession numbers provided in the Data Availability Section?	Yes	See Data Availability Section
Were **human clinical and genomic datasets** deposited in a public access-controlled repository in accordance to ethical obligations to the patients and to the applicable consent agreement?	Not Applicable	
Are **computational models** that are central and integral to a study available without restrictions in a machine-readable form? Were the relevant accession numbers or links provided?	Yes	See Data Availability Section
If publicly available data were reused, provide the respective **data citations in the reference list**.	Not Applicable	
